# Genetic Code Expansion: Recent Developments and Emerging Applications

**DOI:** 10.1021/acs.chemrev.4c00216

**Published:** 2024-12-31

**Authors:** Yujia Huang, Pan Zhang, Haoyu Wang, Yan Chen, Tao Liu, Xiaozhou Luo

**Affiliations:** † State Key Laboratory of Natural and Biomimetic Drugs, Department of Molecular and Cellular Pharmacology, School of Pharmaceutical Sciences, Chemical Biology Center, Peking University, Beijing 100191, China; ‡ Shenzhen Key Laboratory for the Intelligent Microbial Manufacturing of Medicines, Key Laboratory of Quantitative Synthetic Biology, Center for Synthetic Biochemistry, Shenzhen Institute of Synthetic Biology, Shenzhen Institute of Advanced Technology, Chinese Academy of Sciences, Shenzhen 518055, P.R. China; § University of Chinese Academy of Sciences, Beijing 100049, P. R. China

## Abstract

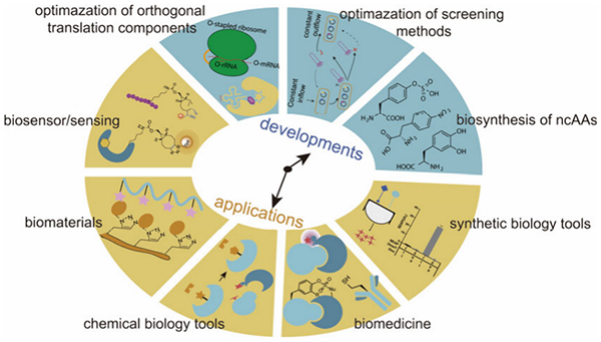

The concept of genetic code expansion (GCE) has revolutionized the field of chemical and synthetic biology, enabling the site-specific incorporation of noncanonical amino acids (ncAAs) into proteins, thus opening new avenues in research and applications across biology and medicine. In this review, we cover the principles of GCE, including the optimization of the aminoacyl-tRNA synthetase (aaRS)/tRNA system and the advancements in translation system engineering. Notable developments include the refinement of aaRS/tRNA pairs, enhancements in screening methods, and the biosynthesis of noncanonical amino acids. The applications of GCE technology span from synthetic biology, where it facilitates gene expression regulation and protein engineering, to medicine, with promising approaches in drug development, vaccine production, and gene editing. The review concludes with a perspective on the future of GCE, underscoring its potential to further expand the toolkit of biology and medicine. Through this comprehensive review, we aim to provide a detailed overview of the current state of GCE technology, its challenges, opportunities, and the frontier it represents in the expansion of the genetic code for novel biological research and therapeutic applications.

## 1 Introduction

Genetic code expansion (GCE) technology significantly advances molecular biology, synthetic biology, and biomedicine by enabling the site-specific incorporation of noncanonical amino acids (ncAAs) into proteins within cells and even sometimes within organisms. This is achieved through the orthogonal pairing of an engineered aminoacyl-tRNA synthetase (aaRS) with a suppressor tRNA, ensuring they function independently of the host’s translational machinery. GCE technology expands the chemical repertoire of proteins, enhancing our understanding of their structure and function and facilitating various innovative applications.^1−3^ These applications include innovative therapeutics,^4^ novel biomaterials,^5^ enhanced drug delivery systems,^6^ and new vaccine strategies,^7^ impacting fields from synthetic biology to medicine. Recent advancements have reinforced GCE technology’s role as a pivotal tool in biotechnological innovation, offering unique opportunities for designing novel proteins with wide-ranging applications.^8^ This paradigm shift heralds a new era of scientific discovery and applications in synthetic biology and biotechnology.

Proteins, as the fundamental building blocks of life, perform a wide range of functions. In nature, the biosynthesis of proteins generally follows the central dogma. Genes are transcribed into mRNAs, and mRNAs are translated to proteins by ribosomes. The GCE technology requires a unique codon and an orthogonal aaRS/tRNA pair.^8^ The unique codons are an essential part of GCE technology. Nowadays, the most widely used codon is amber stop codon, but other methods such as quadruplet codons and sense codon reassignment are also used to create more unassigned codons. Orthogonal aaRS/tRNA pair used for the recognition and acylation of ncAA is prerequisite for the incorporation of the ncAAs. In this process, ncAAs are recognized by their corresponding aaRSs and aaRSs also recognize their cognate tRNAs, after which charging them with the corresponding ncAAs. This means the aaRS/tRNA pair as an additional component has no interaction with the endogenous ones. The ncAA-loaded tRNAs match to the blank codons and successfully incorporate the ncAAs into the polypeptide chain at the desired sites during ribosomal translation. Up to now, there are more than 200 ncAAs^9−11^ that have been site-specifically incorporated in proteins by GCE technology.

In this review, we focus on the developments and advancements of GCE technology mainly over the past five years, covering the optimization of this technology and its applications in fields such as synthetic biology, biomedical science, and biological mechanistic studies. GCE technology, which arises from the intersection of chemistry and biology, encompasses both small molecules like ncAAs with varying structures and biological macromolecules such as aaRSs and tRNAs that are involved in protein translation and various metabolic pathways. Unlike other techniques that introduce ncAAs at specific sites into proteins such as auxotrophy-based methods or solid phase peptide synthesis (SPPS), the distinctiveness of GCE lies in its ability to incorporate ncAAs site-specifically and its application in living cells. Therefore, our discussion will be limited to ncAAs used in GCE and those introduced at specific sites in living cells through nonstandard codon tables, excluding works involving the synthesis of aminoacyl-tRNA with ncAAs in vitro via methods such as ribozymes, as well as the site-specific incorporation of ncAAs through means such as auxotrophic strains. Although unnatural base pairs also play a significant role in the site-specific insertion of ncAAs, given the relative independence and criticality of this area, and the recent reviews^12^ that have extensively explored it, this review will not delve into this aspect further.

## 2 The Introduction of GCE Technology

### 2.1 The Technical Principle of GCE Technology

In the realm of biology, DNA and RNA are fundamental for storing and transmitting hereditary information, while proteins, which make up most of the cell’s dry mass, perform a wide range of essential functions. These natural proteins are constructed from a repertoire of 20 canonical amino acids, each uniquely encoded by one or more specific codons, which together constitute the genetic code. Among the 64 codons available, 61 are dedicated to representing the 20 canonical amino acids, while the remaining three, UAA, UAG, and UGA, act as unequivocal termination signals, orchestrating the cessation of protein synthesis.

The inherent diversity of amino acids, such as polarity, charge, size, and hydrophobicity, endows these molecular building blocks with unique properties. The arrangement of amino acids in a peptide chain profoundly influences the protein’s structure and function. Consequently, the precise translation of codons holds paramount importance in ensuring the accurate synthesis of proteins. Errors in codon recognition or codon mutations can potentially result in changes to protein function, which may ultimately lead to the onset of diseases.

In the pursuit of protein engineering, scientists utilize the sophisticated strategy of codon manipulation to intricately influence amino acid sequences.^13^ However, it is crucial to acknowledge that codon replacement enables the substitution of amino acids constrained to the 20 standard amino acids. This highlights the need for alternative strategies that can overcome these limitations to fully harness the potential of protein engineering.

To overcome these limitations and fully unlock the potential of amino acid diversity, natural proteins employ various strategies, including post-translational modifications (PTMs) such as methylation, phosphorylation, glycosylation, and hydroxylation as well as interactions with coenzymatic factors. While these strategies broaden the functional capabilities of proteins, they fall short of fully exploiting the vast diversity of amino acids and address the evolving challenges in protein-related applications. Consequently, researchers have embarked on various approaches to incorporate ncAAs into proteins.

As early as the 1970s, scientists achieved the synthesis of transfer RNA (tRNA) linked to ncAAs under in vitro conditions and successfully introduced these ncAAs into proteins using cell-free systems.^14^ This method even facilitated the generation of peptide probes for investigating biological processes within cells.^15^ However, challenges arose due to the potential repeated usage of the same codon within a single gene, hindering site-specific ncAA incorporation.

In 1989, Noren et al.^16^ introduced a method wherein codons at desired ncAA incorporation sites were replaced with the stop codon UAG, accompanied by corresponding modifications to the anticodon on tRNA. This approach cleverly bypassed the dependence on the anticodon region of tRNA while mitigating the issue of competition between ncAAs and endogenous amino acids for the same codon. Consequently, this method offered a universal biosynthetic approach for introducing ncAAs into proteins. Following its successful application in cell-free systems, other scientists achieved site-specific ncAA incorporation within living cells. For example, in 1995, Nowak et al.^17^ achieved ncAA incorporation in *Xenopus oocytes* by injecting both synthesized mRNA containing a premature termination codon (PTC) and the corresponding suppressor tRNA containing synthetically acylated ncAA into the cells.

The precise encoding of amino acids is vital for the normal growth and functioning of cells. When aminoacyl-tRNAs are synthesized in vitro, it is easy to ensure the exclusive pairing between amino acids and the corresponding tRNA. However, when it comes to aminoacylating tRNAs within live cells, additional complexities emerge. Beyond introducing stop codons at specific positions within the target gene, it is crucial to ensure that the newly introduced tRNAs and ncAAs remain unrecognized by endogenous aaRSs. Simultaneously, the introduced aaRS should not recognize endogenous tRNAs or other amino acids apart from the intended ncAA.

In response to this challenge, scientists embarked on the development of orthogonal aaRS/tRNA pairs from various species. In 2000, Wang et al.^18^ discovered that the tyrosyl-tRNA synthetase (TyrRS) and the corresponding tRNA^Tyr^ from the archaeon *Methanocaldococcus jannaschii*, when introduced into *Escherichia coli* (*E. coli*), exhibited orthogonality. Building upon this discovery, they subsequently evolved an aaRS capable of recognizing *O*-methyl-l-tyrosine (OMeY), known as *Mj*OMeYRS. This groundbreaking development marked the inaugural implementation of GCE within *E. coli*,^19^ setting the stage for the genetic encoding of ncAAs and marking the dawn of a new chapter in this field.

### 2.2 aaRS/tRNA System

The cellular machinery responsible for encoding ncAAs comprises three main components: aaRS/tRNA pairs, mRNAs transcribed from the target gene with one or more blank codons, and ncAAs themselves. Among these, the aaRS/tRNA pairs are the cornerstone of GCE technology.

To ensure orthogonality within cellular contexts, scientists often source aaRS/tRNA pairs from organisms that are evolutionarily distant from the target system. Typically, aaRS/tRNA pairs from archaea and eukaryotes exhibit better orthogonality in prokaryotic systems, except for the archaea-derived pyrrolysine system (PylRS/tRNA pair), which is orthogonal in both eukaryotic and prokaryotic systems. Conversely, aaRS/tRNA pairs derived from bacteria are generally more suitable for eukaryotic contexts. For example, the archaeal *Mj*TyrRS/*Mj*tRNA^Tyr^ pair^19^ demonstrates orthogonality when introduced into *E. coli*. Similarly, in *Saccharomyces cerevisiae*, the bacterial *Ec*TyrRS/*Ec*tRNA^Tyr^ pair exhibits biological orthogonality.^20^

While it is plausible for the *Ec*TyrRS/*Ec*tRNA^Tyr^ pair to exhibit orthogonality in mammalian cells, significant challenges arise due to the profound differences in tRNA transcription and processing between mammalian cells and *E. coli*. For instance, *E. coli* tRNA is transcribed from an upstream promoter, whereas mammalian tRNA is transcribed from an internal promoter. Moreover, mammalian tRNA requires additional post-transcriptional modifications at the 3′-end for functionality. Consequently, scientists initially utilized *Bs*tRNA^Tyr^ from *Bacillus stearothermophilus*, which possesses an internal promoter, for GCE in mammalian cells.^21^ It was not until 2007 that Wang et al.^22^ successfully applied *Ec*TyrRS/*Ec*tRNA^Tyr^ in mammalian cells by introducing an additional promoter upstream of *Ec*tRNA^Tyr^. This additional promoter can be recognized by RNA polymerase III in mammalian cells, which is responsible for the transcription of mammalian tRNA genes.

The initial development of TyrRS/tRNA^Tyr^ pairs as orthogonal aaRS/tRNA pairs for GCE marked a significant milestone. Shortly thereafter, researchers uncovered a natural occurrence in *Methanosarcina barkeri* where the *Mb*PylRS/*Mb*tRNA^Pyl^ pair effectively decoded the UAG stop codon to pyrrolysine.^23,24^ The PylRS/tRNA^Pyl^ pair, owing to their archaeal origin and intrinsic ability to decode stop codon, demonstrates remarkable biological orthogonality, functioning effectively in both prokaryotic and eukaryotic organisms. Moreover, PylRS exhibits a wide-ranging substrate specificity. Consequently, scientists have harnessed PylRS/tRNA^Pyl^ pairs from various species, including *Desulfitobacterium hafniense*,^25^*Methanosarcina mazei*,^26^*Methanomethylophilus alvus*,^27^*Methanogenic archaeon*,^28^*Methanococcoides burtonii*,^29^*Methanosarcina thermophila*,^30^ as well as *Methanosarcina flavescens*,^30^ to facilitate GCE. A comprehensive exploration of these advancements will be presented in subsequent sections.

While TyrRS/tRNA^Tyr^ and PylRS/tRNA^Pyl^ stand as the most extensively employed orthogonal aaRS/tRNA pairs for GCE, scientists have made successful strides in developing additional orthogonal aaRS/tRNA pairs. These encompass PheRS/tRNA^Phe^,^31−33^ GlnRS/tRNA^Gln^,^34,35^ AspRS/tRNA^Asp^,^36^ LeuRS/tRNA^Leu^,^37,38^ GluRS/tRNA^Glu^,^39^ LysRS/tRNA^Lys^,^40^ TrpRS/tRNA^Trp^,^41,42^ HisRS/tRNA^His^,^43^ ProRS/tRNA^Pro^,^44^ among others. Nevertheless, it is noteworthy that only a subset of these aaRS/tRNA pairs has demonstrated effectiveness in facilitating site-specific incorporation of ncAAs. Furthermore, in specific biological contexts, the generation of Seryl-tRNA involves a multistep enzymatic pathway. In such scenarios, SepRS/tRNA^Sep^, employed to produce the intermediate phosphoseryl-tRNA (Sep-tRNA), has also found applications in the realm of GCE.^45^

### 2.3 GCE Technology in Various Model Systems

The GCE technology has witnessed extensive utilization in various biological model systems, including *E. coli*,^46^*S. cerevisiae*,^22^ and mammalian cells,^22^ over a substantial period. Nevertheless, researchers have consistently dedicated their endeavors to expanding the spectrum of organisms amenable to the applications of the GCE technology, enhancing its versatility and application breadth.

For example, GCE has been successfully extended to prokaryotic organisms beyond *E. coli*, encompassing *Shigella flexneri*,^47^*Salmonella*,^47−49^*Candida albicans*,^50^*Pseudomonas aeruginosa*,^51^*Mycobacterium tuberculosis*,^52^*Streptomyces venezuelae*,^53^*Pseudomonas putida*^54^ and *Bacillus subtilis*.^55^ This evolution marks GCE as an essential tool for probing protein–protein interactions in bacterial contexts. Moreover, the applications of GCE technology in pathogenic bacteria enables the development of strains that can only survive in environments with ncAA, paving the way for the creation of live bacterial vaccines.

In addition, by applied GCE technology in specific species, researchers can also increase the yield of proteins produced using GCE technology. In 2021, González et al.^56^ pioneered the implementation of GCE technology in a commercially available *Vibrio natriegens* strain (Vmax X2), renowned for its exceptional capacity to produce soluble proteins. When they introduced 5 UAG codons to the superfolder green fluorescent protein (sfGFP) gene and expressed sfGFP in Vmax X2 and different *E. coli* strains by GCE technology, the yields of ncAA-incorporated sfGFP expressed in Vmax X2 were at least 2-fold higher than those from traditional expression strains such as *E. coli* Top10 and 1.5-fold higher than *E. coli* BL21. Moreover, Vmax X2 cells exhibit significantly reduced levels of misincorporation at the UAG codon.

While prokaryotes, including *E. coli*, continue to serve as prominent choices for recombinant protein production, it is crucial to recognize the challenges posed by the expression of complex proteins and protein complexes, particularly those of eukaryotic origin, within these simple systems. The GCE technology has thus been extended to eukaryotic cells, including *S. cerevisiae* and HEK293, and even to *Xenopus laevis* oocytes.^57^ In 2016, Koehler et al.^58^ established an efficient and robust protein production platform known as “MultiBacTAG” by integrating the GCE elements into MultiBac insect cells. Furthermore, GCE has extended its reach to human stem cells, including hematopoietic stem cells (HSCs)^59^ and human induced pluripotent stem cells (hiPSC),^60^ facilitating the derivatization of the entire hematopoietic system with ncAAs and the introduction of expanded genetic codes into hiPSC-derived neurons and brain organoids.

Furthermore, scientists have expanded the application of the GCE technology from cultured cells and unicellular organisms to multicellular organisms. For example, the nematode *Caenorhabditis elegans*, known for its small genome and ease of laboratory maintenance, was one of the first multicellular organisms to benefit from this technique. Greiss et al.^61^ pioneered the expansion of the genetic code of *C. elegans* with *Mm*PylRS/*Mm*tRNA_CUA_ in 2011. Subsequently, Bianco et al.^62^ extended the genetic code of *Drosophila melanogaster*, a prime model for genetic analysis and mutagenesis screens. In 2013, Li et al.^63^ further incorporated photoclick ncAAs into *Arabidopsis thaliana*, a model organism widely utilized in plant biology research.

Considering its status as the preeminent model organism for investigating human physiology and disease, *Mus musculus* possesses a genome closely analogous to that of humans. Consequently, there has been a keen interest in applying GCE technology within the mouse model. In 2016, Ernst et al.^64^ expanded the genetic code in the brain of *M. musculus* with adeno-associated viral (AAV) vectors. Subsequently, by delivering linearized construct into nuclei of mouse zygotes, Chen et al.^65^ and Han et al.^66^ integrated orthogonal tRNA/aaRS pairs into the genome of *M. musculus*. Also, Shi et al.^67^ generated transgenic mice containing PylRS/tRNA pairs using CRISPR/Cas9 technique and restored dystrophin expression in mice by GCE technology.

The zebrafish (*Danio rerio*), another vertebrate model apart from *M. musculus* that is evolutionarily closer to human, has emerged as an ideal model for studying vertebrate development and genetically tractable diseases. In 2016 and 2017, Chen et al.^65^ and Liu et al.^68^ successfully expanded the genetic code of *D. rerio*, respectively.

The extension of the GCE technology beyond conventional model organisms provides researchers with a powerful tool for modulating protein functionality in living cells. This technique is particularly advantageous due to its flexibility and adaptability. And it enables precise incorporation of ncAAs, offering more control over protein engineering. The continued application and refinement of this technology are expected to open new and innovative avenues for biological research, enabling deeper and more precise insights.

## 3 The Recent Developments of GCE Technology

The recent advancements in GCE technology have opened new frontiers in protein engineering. The following sections will delve into the optimization of aaRS, tRNA, and other components of the translation system, as well as the optimization of screening methods and the biosynthesis of ncAAs.

### 3.1 Optimization of aaRSs

#### 3.1.1 Optimization Based on Thermally Stable aaRSs

**Figure 1:**
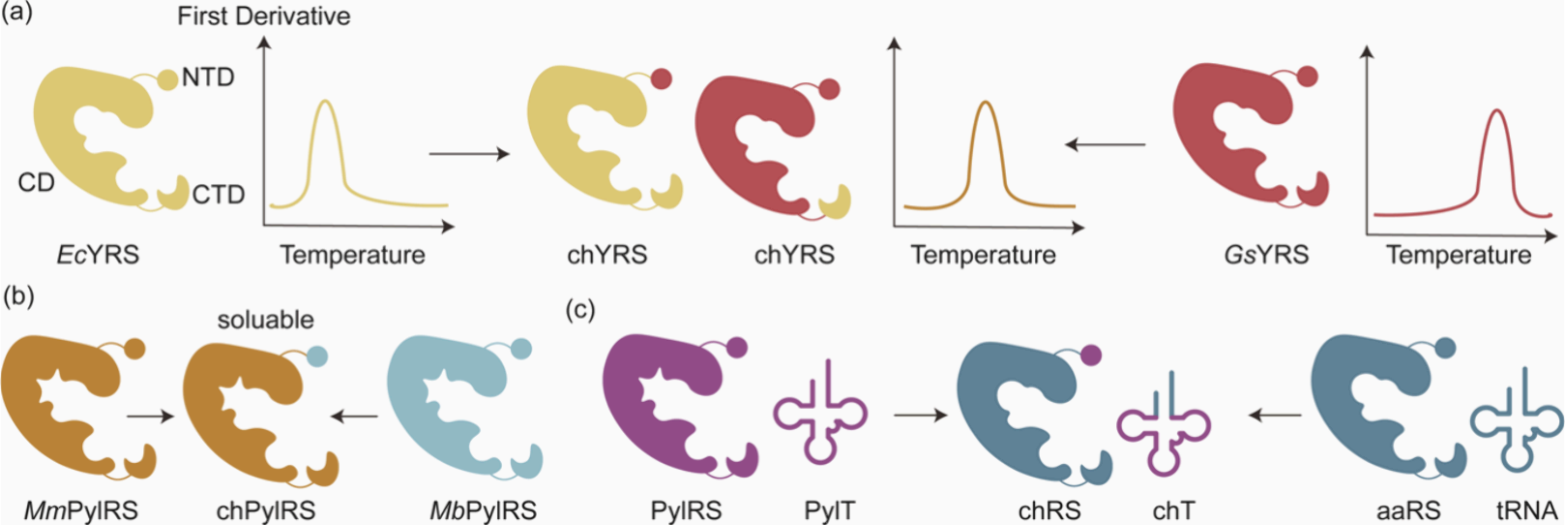
Chimeric aaRS/tRNA pairs. (a) Bacterial TyrRS chimeras created from *Gs*TyrRS and *Ec*TyrRS exhibit higher activity and intermediate thermostability. (b) Fusion of *Mb*PylRS N-terminal domain to the *Mm*PylRS C-terminal domain, creating a novel chimeric PylRS. (c) Transplanting the key orthogonal components from the pyrrolysine system (aaRS and tRNA) to those for canonical amino acid systems can create chimeric aaRS/tRNA pairs. Abbreviations: NTD, N-terminal domain; CD, catalytic domain; CTD, C-terminal domain; ch, chimeric; aaRS, aminoacyl-tRNA synthetase.

In recent years, researchers have made significant efforts in the development of new aaRS/tRNA pairs. Beyond traditional methods of screening and optimization, scientists have pursued innovative approaches. One notable strategy has been the exploration of extremophiles, particularly thermophiles, in search of aaRS/tRNA pairs that are inherently more stable and amenable to modification (Figure 1a).

In 2019, Xuewen Qin et al.^69^ identified a novel class of TyrRS/tRNA^Tyr^ pairs from the thermophilic bacterium *Geobacillus stearothermophilus*. Although these pairs were homologous to the *Ec*TyrRS, they exhibited enhanced thermal stability. This new pair demonstrated orthogonality in both mammalian cells and *S. cerevisiae*, making them suitable for GCE. They could charge various ncAAs such as *p*-azido-l-phenylalanine (pAzF) and *p*-borono-l-phenylalanine (pBoF), with comparable cellular efficiency, improved specificity, and lower background compared to their *E. coli* homologues. This thermally stable enzyme offers an alternative scaffold for synthetase library screening or evolution, enabling the genetic encoding of structurally more complex ncAAs in eukaryotic cells, such as those containing phospho- or sulfo- functional groups. Subsequently, in 2021, Katherine T. Grasso et al.^70^ developed chimeras between *Ec*TyrRS and its homologue from thermophilic bacteria, offering an optimal balance between thermal stability and activity. The results indicated that the chimeric bacterial TyrRS showed increased tolerance to destabilizing mutations in the active site.

Similarly, in 2020, Liming Hu et al.^30^ sought and characterized stable PylRS homologues from thermophiles. They discovered two moderately thermophilic *Methanoculleus marisnigri* strains and demonstrated that both PylRS were orthogonal and active in *E. coli* and mammalian cells, hence suitable for GCE. Bloom et al.^71^ previously showed that having higher melting temperatures (*T*_m_) is linked to increased protein stability, which promotes evolvability. Thus, increasing protein stability can enhance the ability of proteins to evolve new functions. Numerous studies have suggested that changes^72−75^ in enzyme specificity often decrease thermodynamic stability, especially mutations in the substrate binding pocket that alter specificity.^76^ Therefore, achieving higher *T*_m_ and enhanced expression levels is relevant as intrinsic structural robustness or stability is a crucial factor in engineering effective aaRS mutants. Indeed, Liming Hu et al. developed a series of mutants based on these PylRS exhibiting higher melting temperatures and enhanced expression levels in HEK293 cells.

#### 3.1.2 Chimeric aaRS/tRNA Pairs

In addition to fusing aaRSs with their more thermally stable versions, alternative chimeric approaches are also explored. For example, In 2017, Suzuki et al.^77^ designed a novel chimeric PylRS (chPylRS, Figure 1b), in which the *Mb*PylRS N-terminal domain (NTD, 1–149 residues) was fused to the *Mm*PylRS C-terminal domain (CTD, 185–454 residues). This chimera was selected because of its improved solubility compared to that of nonchimeric proteins. chPylRS and its derivatives have been applied to encoding different ncAAs such as *N*^ε^-(*tert*-butoxycarbonyl)-l-lysine (BocK), *N*^6^-(((trimethylsilyl)methoxy)carbonyl)-l-lysine (TMSK),^78,79^*N*_ε_-propargyl-l-lysine (PrK) and *N*_ε_-bicyclononyne-l-lysine (BCN).^79^

Scientists have also experimented with the fusion of aaRS and their corresponding tRNAs with entirely different aaRS/tRNA pairs to develop novel orthogonal aaRS/tRNA pairs (Figure 1c). In 2020, Wenlong Ding et al.^80^ transplanted the key orthogonal components from the PylRS/tRNA^Pyl^ to those for histidine, phenylalanine, and alanine systems, creating multiple chimeric aaRS/tRNA pairs. Their data indicated that these engineered chimeric systems were orthogonal and efficient, possessing a flexibility comparable to that of the pyrrolysine system. Notably, the chimeric phenylalanine system demonstrated the ability to effectively incorporate a set of phenylalanine, tyrosine, and tryptophan analogues in both *E. coli* and mammalian cells. Building upon this foundation, in 2021, Hongxia Zhao et al.^81^ conducted systematic directed evolution to design a chimeric PheRS/tRNA system with enhanced incorporation efficiency, achieving expression levels of different proteins such as GFP, adenylate kinase, and ubiquitin containing ncAAs such as 4-azido-Phe (AzF), 4-acetyl-Phe (AcF), 2-naphthyl-Ala (NapA), 6-methyl-Trp (6MW), or 7-methyl-Trp (7 MW) at single sites. With these novel aaRS/tRNA pairs, the expression level of target proteins surpassed or were similar to those of wild-type proteins. Additionally, it exhibited a significantly reduced rate of protein production in the absence of AzF.

#### 3.1.3 Transforming PylRS into Mutually Orthogonal Versions

An alternative approach of developing novel aaRS/tRNA pairs involves the modification of existing ones. For instance, in 2018, Julian C. W. Willis et al.^28^ demonstrated the activity and orthogonality of several ΔNPylRS/Pyl tRNA_CUA_ pairs (where PylRS lacks the N-terminal domain) in *E. coli*, effectively incorporating ncAAs. They created new PylRS/tRNA^Pyl^ pairs that were orthogonal to the *Mm*PylRS/*Mm*tRNA^Pyl^ pair. This study illustrated that transferring mutations related to ncAA specificity of *Mm*PylRS into the new PylRS could reprogramm their substrate specificity in a predictable manner. Furthermore, these different derived PylRS/tRNA^Pyl^ pairs could function within the same cell, decoding different codons and integrating various ncAAs.

Building upon this work, Daniel L. Dunkelmann et al.^82^ discovered that the sequences of these novel ΔNPylRS/tRNA pairs could be categorized into two distinct classes. Subsequently, they identified 18 mutually orthogonal ΔNPylRS/tRNA pairs and generated a set of 12 triple orthogonal pairs. The engineered triple orthogonal pairs derived from this study facilitate the integration of three different ncAAs into a single protein within a cell. Later, in 2023, Adam T. Beattie et al.^83^ identified two novel categories of PylRS/tRNA^Pyl^ pairs, distinct from the previously known types. Their findings also revealed that the majority of these PylRS enzymes and tRNA^Pyl^ exhibited activity and orthogonality in *E. coli*. Utilizing tRNA^Pyl^ engineering and directed evolution techniques, the team successfully created an extensive array of orthogonal pairs: 924 mutually orthogonal PylRS/tRNA^Pyl^ pairs, 1324 triple orthogonal pairs, 128 quadruple orthogonal pairs, and 8 quintuple orthogonal pairs.

### 3.2 Optimization of tRNAs

#### 3.2.1 Optimization of tRNA Transcription

In addition to the extensive exploration of developing aaRS/tRNA pairs, scientists have also dedicated significant efforts toward the refinement of tRNA transcription due to tRNA’s crucial role in GCE. The availability of tRNA is subject to various influencing factors, especially since the transcription and processing of tRNA can vary significantly across different species. One major challenge in tRNA expression is the divergence in transcription mechanisms among species. In eukaryotes, tRNAs are transcribed by RNA polymerase III, and their structural genes contain embedded A- and B-box promoter sequences. In contrast, bacterial and archaeal tRNAs lack these internal promoter sequences, necessitating alternative strategies for effective transcription in eukaryotic systems. For example, in bacterial systems, it is common to append the naturally absent terminal CCA sequence to the 3′-end of archaeal tRNAs to mimic bacterial tRNAs. Conversely, in eukaryotic systems, the 3′-CCA sequence is often removed from bacterial orthogonal tRNA genes to ensure proper processing and functionality.^84^ To address the absence of promoter sequences in orthogonal tRNAs from prokaryotes, various strategies have been employed. For example, extragenic RNA polymerase III promoters^22^ have been utilized to compensates for the lack of internal promoters in prokaryotic tRNAs by using external promoter sequences recognized by RNA polymerase III. The adoption of strong RNA polymerase II promoters featuring tandem tRNA repeats^85^ can also enhance tRNA expression. And the fusion of yeast tRNA^Arg^^86^ (as part of a dicistronic construct) upstream of the target tRNA is another strategy to facilitate tRNA expression in the desired context. Additionally, inducible GCE systems, such as T-Rex,^87^ offer another strategy to optimize tRNA transcription and minimize the risk of adverse effects on cells caused by the overproduction of unused tRNA.

#### 3.2.2 Increasing tRNA Copy Number

Robust promoters are essential to ensure the efficient expression of tRNA. Nevertheless, Schmied et al.^88^ have demonstrated that the amount of tRNA genes introduced is also fundamental in the readthrough of stop codons. While tRNA copy number does not pose a significant challenge in transient transfection scenarios, where it leads to substantial intracellular plasmid accumulation and nearly saturated transgene expression, it proves to be a formidable hurdle for alternative methodologies like viral delivery and the stable integration of the aaRS/tRNA system into mammalian cells. This challenge arises due to the limited cargo capacity of the viral vector and the genome’s susceptibility to recombination, which, in turn, imposes constraints on the utilization of more than one or two copies of tRNA per virus genome. This limitation significantly hinders the efficiency of high-level expression.

In 2016, Zheng et al.^89^ embraced the baculovirus vector for GCE in mammalian cells. The baculovirus vector boasts an ultra large cargo capacity exceeding 30 kb and is characterized by high genome stability. Remarkably, this vector can accommodate up to 20 tRNA expression cassettes per genome, leading to a substantial enhancement in ncAA incorporation efficiency even at low viral loads. Additionally, their research provided an estimate suggesting that achieving efficient ncAA incorporation may necessitate the presence of over a hundred tRNA expression cassettes per cell. Subsequently, Roy et al.^90^ developed two stable cell lines (containing wild type and mutated PylRS/tRNA pair, respectively) capable of efficient ncAA incorporation by employing linearized plasmids encoding pylRS and 18 tandem repeats of tRNA^Pyl^. The two resulting cell lines demonstrated the capacity to produce ncAA-containing IgG at levels ranging from 1.5 to 3.0 g/L.

#### 3.2.3 Optimization of Interactions between tRNA and Translation Components

In addition to tRNA copy numbers, the interactions between tRNA and other translation machineries can also influence the efficiency of ncAA incorporation. In the context of GCE technology, it is necessary to convert the natural codon of the orthogonal tRNA to the corresponding anticodon for the stop codon. For tRNAs that interact with aaRS through their anticodon region, this process can potentially hinder tRNA aminoacylation. Therefore, a strategy for optimizing tRNA involves altering sequences surrounding the anticodon region to enhance the suppression efficiency of orthogonal tRNAs. For example, through systematic evolution of the sequence surrounding the anticodon of tRNA^Sep^ and subsequent evolution of the anticodon recognition region of SepRS, Rogerson et al.^45^ successfully achieved an 18-fold improvement in pSer incorporation efficiency compared to previously reported pairs. Importantly, this evolution not only enhanced efficiency of tRNA^Sep^ but also improved its specificity, reducing the likelihood of mis-aminoacylation with glutamine by Gln *R*S due to alterations in the tRNA anticodon region.

After aminoacylation, the ncAA-tRNAs will form a complex with the elongation factor and GTP, facilitating their transportation to the ribosome to participate in protein translation. The binding affinity between an aminoacyl-tRNA and the elongation factor plays a crucial role in determining translation efficiency. If the binding is too weak, it may impede the formation of an efficient complex, while excessively tight binding can prevent the timely dissociation of the complex during the translation process. In 2009, Guo et al.^91^ devised a strategic approach involving the randomization of regions within *Mj*tRNA^Tyr^ responsible for its interactions with the elongation factor. This tRNA library ultimately led to the identification of tRNA variants that substantially augmented the production of proteins containing ncAAs. Similar efforts have been directed toward tRNA^Pyl^. In a study by Fan et al.,^92^ targeted mutations were introduced into the acceptor stem and T stem of tRNA^Pyl^. The resultant tRNA^Pyl^-opt variant exhibited an impressive 5-fold enhancement in its ability to simultaneously incorporate ncAAs into two distinct positions within the target protein.

In addition to mutagenesis and screening, phage and virus-assisted directed evolution techniques have arisen as valuable strategies for enhancing the evolution of orthogonal tRNA in both prokaryotic and eukaryotic systems, resulting in significant improvement of interactions between tRNA and the translation apparatus.^93−95^

#### 3.2.4 Developments of Chimeric tRNAs

In addition to targeting specific regions within tRNA molecules, another approach to optimize orthogonal tRNAs draws from the structural features of natural tRNAs. In 2007, Ambrogelly et al.^96^ undertook a pioneering endeavor by grafting the identity elements of tRNA^Pyl^ into the bovine mitochondrial tRNA^Ser^ (mt-tRNA^Ser^) scaffold, yielding chimeric tRNAs that displayed robust activity both in test tubes and in *E. coli*. Similarly, in 2017, Serfling et al.^97^ engaged in the transplantation of various combinations of tRNA recognition motifs for PylRS into the bovine mt-tRNA^Ser^. Compared to the original tRNA^Pyl^, the engineered tRNAs manifest elevated intracellular concentrations, and display partially distinct post-transcriptional modifications. Furthermore, Serfling et al.^97^ created another distinct set of *Mm*tRNA^Pyl^ by substituting nucleotides with those aligned with patterns observed in human tRNAs. Notably, these tRNAs exhibited intracellular concentrations 2–5-fold higher than that of tRNA^Pyl^.

Tharp et al.^98^ have also introduced an additional chimeric tRNA, constructed by incorporating motifs from both *Mj*tRNA^Tyr^ and *E. coli* initiator tRNA (itRNA). This novel hybrid tRNA maintains the initiation capability while losing the capacity to elongate polypeptide chains. Consequently, it can effectively initiate translation using ncAAs with the UAG codon. Subsequently, the *Mj*TyrRS/itRNA^Tyr^ pair was employed in conjunction with an orthogonal PylRS/tRNA pair. This combined system facilitated the synthesis of a protein that integrated two distinct ncAAs at the first and second positions. Furthermore, itRNA^Tyr^ underwent engineering to create a mutant capable of initiating translation with ncAAs responding to the UAU codon.^99^ Using this itRNA^Tyr^ with endogenous elongator tRNA^Tyr^, UAU codon exhibited dual functionality, selectively encoding ncAAs at the initiating position while predominantly incorporating tyrosine at elongating positions. While using two mutually orthogonal PylRS/tRNA pairs in one cell, the UAU codon can be reassigned along with UAG and UAA codons simultaneously, thereby enabling the production of proteins containing two or three distinct ncAAs.

#### 3.2.5 Post-Transcriptional Modifications of tRNAs

Besides all these factors mentioned earlier, the modification of tRNA nucleotides can significantly influence suppression efficiency and specificity. In 2018, Crnkovic et al.^100^ reported that the deletion of the cysteine desulfurase gene (*iscS*), which is involved in sulfur-containing post-transcriptional tRNA modifications by activating sulfur from free l-cysteine, resulted in an over 8-fold increase in the yield of Sep-containing proteins. Conversely, the overexpression of *E. coli* dimethylallyltransferase (MiaA) and pseudouridine synthase (TruB) improved the purity of the Sep-containing proteins, indicating enhanced tRNA specificity. This improvement is likely attributed to MiaA and TruB increasing the fraction of tRNA^Sep^ with i6A37 and Ψ55 modifications, respectively. TruB may also facilitate the folding of tRNA^Sep^. Other studies^101^ have also emphasized how tRNA modifications can impact its interaction with elongation factors. These findings underscore the significance of considering tRNA modifications when designing suppression tRNAs. Moreover, biological components and pathways related to tRNA modification can be leveraged to enhance GCE.

### 3.3 Optimization of Other Components of Translation System

In addition to optimizing the key components, such as aaRS and tRNA, scientists have also made numerous enhancements in various aspects of translation-related system. These improvements include advancing release factors, elongation factors, introducing new codons, developing orthogonal ribosomes, strategies to mitigate target mRNA degradation, adjusting context codons, minimizing interference between aaRS/tRNA pairs and other cellular components, as well as modefying and improving host strains or cells.

#### 3.3.1 Modification of Release Factors

One notable limitation of the GCE technology that utilizes stop codons is the potential competition between suppression tRNA and release factors (RFs), which can lead to premature termination of protein synthesis at the introduced stop codon, consequently diminishing the efficiency of ncAA incorporation. Because simply knocking out the *pfrA* gene (encoding RF1) from *E. coli* is lethal, Mukai et al.^102^ addressed this challenge by introducing a conditional *pfrA* mutant in 2010. They inserted an arabinose promoter together with the regulator gene *araC* into the *E. coli* genome upstream of *hemA-prfA-hemK* operon. In this way, the expression of RF1 can be induced by l-arabinose and repressed by d-glucose in the growth media. This genetic manipulation successfully reassigned the UAG codon as a sense codon.

Subsequently, in 2011, Johnson et al.^103^ eliminated RF1 from *E. coli* by a “fixing” RF2 strategy. The expression of RF2 in *E. coli* is regulated by an in-frame UGA codon and the RF2 A246T mutation in K-12 derived *E. coli* strains lowers the release activity of RF2. Thus, they removed the in-frame UAG and fixing the RF2 A246T mutation, generating the “fixed” *prfB*^*f*^ gene. This method enabled efficient incorporation of ncAAs at multiple UAG sites within the same gene.

Also, RF1 has been completely deleted in several *E. coli* strains,^102−106^ facilitating the incorporation of ncAAs at multiple sites. This approach was also extended to eukaryotic cells. In 2014, Schmied et al.^88^ engineered eRF1 mutants to enhance the incorporation of ncAAs in response to the UAG stop codon, without significantly increasing readthrough of UAA and UGA codons. In 2022, Shi et al.^107^ further optimized eRF1, substantially improving the encoding efficiency of three premature stop codon sites in one gene from 0.78% to 11.6%. This advancement allowed the genetic encoding of three distinct ncAAs into a single protein in mammalian cells.

#### 3.3.2 Modification of Elongation Factors

In addition to release factors, the elongation factor-Tu (EF-Tu) assumes a key role in the binding and translocation of aminoacyl-tRNA to the ribosome. In addition to the alterations to tRNA sequences with the aim of augmenting their interaction with EF-Tu, substantial endeavors have been focused on the engineering of EF-Tu itself to amplify the efficiency of incorporating ncAAs. For instance, in 2011, Park et al.^108^ devised a comprehensive library of EF-Tu variants in *E. coli*, leading to the creation of an engineered EF-Tu mutant denoted as EF-Sep, proficient in incorporating the negatively charged phosphoserine (pSer).^109^ This significant advance was further improved by Lee et al.,^110^ leading to the development of EF-Sep21, a variant that enables the production of pSer containing recombinant proteins with remarkable yield. Analogous strategies have been employed to engineer EF-Tu for the accommodation of *O*-phospho-tyrosine^111^ and selenocysteine^112^ as well.

#### 3.3.3 Expanding Codon Repertoire

Owing to the dual functionality of the commonly employed ncAA-encoding codon UAG, which also serves as a stop codon in most species, there exists the potential for inadvertent integration of ncAAs into proteins other than the intended target protein. Consequently, a straightforward approach is to eliminate the native UAG codon within the organism, reserving it exclusively for the encoding of ncAAs. In 2011, Isaacs et al.^113^ conducted an extensive conversion of all UAG codons to synonymous UAA codons in parallel across 32 distinct *E. coli* strains, employing the multiplex automated genome engineering (MAGE) technique. Likewise, the comprehensive replacement of UAG codons with UAA codons within the yeast Sc2.0 genome was subsequently achieved.^114^ More recently, Chen et al.^115^ accomplished multiplexed base editing to convert UAG into UAA codons in HEK293T cells using gRNA arrays, targeting at up to 33 out of 47 genomic sites in a single delivery, which demonstrated the feasibility of recoding mammalian cells. Lajoie et al.^106^ replaced all UAG codons with UAA in *E. coli*, concurrently removing RF1 in 2013. This genomically recoded organism (GRO), named as C321.ΔA, subsequently underwent evolutionary refinement into a modified strain capable of robust growth.^116^ C321.ΔA exhibited substantial but not complete resilience against a diverse panel of viruses^117^ through the aid of tmRNA.^118^ tmRNAs are special molecules that can help to add a tag to half-formed proteins and leading to protein degradation. The codon table of C321.ΔA is different from other viruses. With the help of tmRNA, protein translation from horizontally transmitted genes got stalled on the blank codon, tagged, and destroyed. This underscores the potential of novel genetic codes to strengthen viral resistance, showcasing the organism’s improved resilience through genetic innovation.

Expanding the scope of codon reprogramming from stop codons to sense codons through codon compression, exchange, and expansion could potentially enhance viral resistance. This involves using strategies like compressing multiple codons into fewer ones, swapping codons, and introducing new codons to encode ncAAs. Researchers have explored possibilities to leverage rare sense codons to encode ncAAs, such as AUA^119^ for isoleucine or AGG^120,121^ for arginine.

However, it is important to recognize that this strategy may induce extensive proteome-wide alterations.^122^ As a mitigation measure, substituting corresponding codons in essential genes with synonymous codons in the host cell might be necessary to minimize potential disruptions.^120^ In 2013, Lajoie et al.^123^ achieved the successful elimination of 13 rare codons (including 11 sense codons and 2 stop codons) within 42 highly expressed essential genes across 80 distinct *E. coli* strains, thereby demonstrating the feasibility of radically altering the gentic code. Building on the previous work, Ostrov et al.^124^ comprehensively reduced the total number of genetic codons in *E. coli* from 64 to 57 in 2016. This was accomplished through the removal of the UAG codon and the substitution of six sense codons with synonymous alternatives across all protein-coding genes. However, only 91% of tested essential genes retained functionality with limited fitness effect. Thirteen recoded essential genes became lethal when codon was synonymously replaced.

In the same year, Wang et al.^125^ conducted synonymous substitutions within the *E. coli* cell division operon by replicon excision for enhanced genome engineering through programmed recombination (REXER). Subsequently, this research team undertook a convergent total synthesis endeavor to replace the 4-megabase genome of *E. coli* strain MDS42 with a refactored synthetic genome, leading to the removal of two sense codons (TCA and TCG codon encoding Ser) and one stop codon (TAG).^126^ This new *E. coli* strain utilizes only 61 codons for protein synthesis, designated as “Syn61”. Robertson et al.^127^ later conducted further refinements to expedite the growth rate of Syn61. These enhancements encompassed the utilization of random parallel mutagenesis and an automated dynamic parallel selection strategy. Furthermore, the research team deleted redundant genes encoding tRNA^Ser^_UGA_, tRNA^Ser^_CGA_, and RF1, followed by subsequent rounds of mutagenesis and selection for faster growth. The resulting Syn61Δ3 strain exhibited a remarkable capacity for the synthesis of noncanonical polymers and macrocycles with ncAAs.^128^

Expanding upon this research, Zürcher et al.^129^ extended their study to reassign the TCA and TCG codons of Syn61Δ3 to alanine, histidine, leucine, or proline, thus yielding 16 novel genetic code combinations. Through the development of an orthogonal resistance gene, O-SpecR, wherein TCG encoded alanine and TCA encoded histidine, this artificial genetic code–decoder pair became indispensable. As a consequence, this genetically engineered strain not only achieved full resistance against phages but also established a significant restriction on the transfer of genetic material between organisms employing divergent genetic codes. Likewise, Nyerges et al.^130^ conducted a screening process with an anticodon-swapped tRNA^Leu^ library, through which they successfully identified tRNAs encoding leucine via the TCA and TCG (together TCR) codons. Because elements capable of decoding TCR codons as serine has been deleted in Syn61Δ3, the introduction of these tRNA^Leu^_YGA_ into Syn61Δ3 conferred resistance against viral infections by misleading phage protein translation. Additionally, plasmids carrying the engineered tRNAs and associated antibiotic resistance genes were effectively sequestered, preventing their escape. These endeavors collectively established a genetic-code-based, mutually orthogonal firewall within the horizontal gene transfer (HGT) system.

An alternative approach involves the utilization of tRNA mutants with extended anticodon loops, featuring 8-base loops instead of the typical 7-base loops, thereby creating apparent quadruplet anticodons capable of inducing +1 frameshifts. This approach significantly expands the repertoire of available codons for encoding ncAAs. In theory, a quadruplet codon table could introduce up to 256 unique codons, further extending the genetic code. In 2004, Anderson et al.^40^ engineered a LysRS/tRNA pair derived from *Pyrococcus horikoshii* that selectively incorporates the ncAA l-homoglutamine in response to the AGGA codon in *E. coli*. Subsequently, Neumann et al.^131^ and Chatterjee et al.^132^ achieved AGGA and UAGA decoding, respectively, using the *Mj*TyrRS/tRNA pair, while Wan et al.^133^ and Niu et al.^134^ decoded UAGA and AGGA utilizing the PylRS/tRNA pair. Additionally, Chatterjee et al.^44^ developed a *Ph*ProRS/tRNA pair for AGGG and CUAG codons. By combining one amber codon and one quadruplet codon or employing multiple quadruplet codons simultaneously with mutually orthogonal aaRS/tRNA pairs, researchers achieved the decoding of two,^28,135,136^ three,^82^ or even four distinct ncAAs^137^ concurrently. In a noteworthy development in 2021, Xi et al.^138^ extended the quadruplet codon decoding system to an animal model, the nematode worm *C. elegans*.

Despite the promising potential, the overall efficiency of quadruplet codon decoding remains relatively modest. This challenge arises because tRNAs designed for quadruplet codon decoding typically feature expanded anticodon loops, which can weaken their recognition by aaRSs. To address this issue, modifications to the anticodon-binding region of the corresponding aaRSs have been explored,^131,135^ offering a partial solution. Additionally, enhancing suppression efficiency involves eliminating the competing recognition of triplet codon tRNAs for quadruplet codons.^139^ However, another crucial factor cannot be overlooked: the need for proper interactions between ribosomes and quadruplet tRNAs (qtRNA). By assessing structure-guided libraries in the decoding center, orthogonal ribosomes that efficiently translate quadruplet codons have been developed, which will be further explained in the next section.

Besides, application of unnatural base pairs also represents another approach for expanding the genetic code and achieving site-specific incorporation of ncAAs, which has been elaborately detailed elsewhere.^12,140^

#### 3.3.4 Engineering of Ribosomes

**Figure 2:**
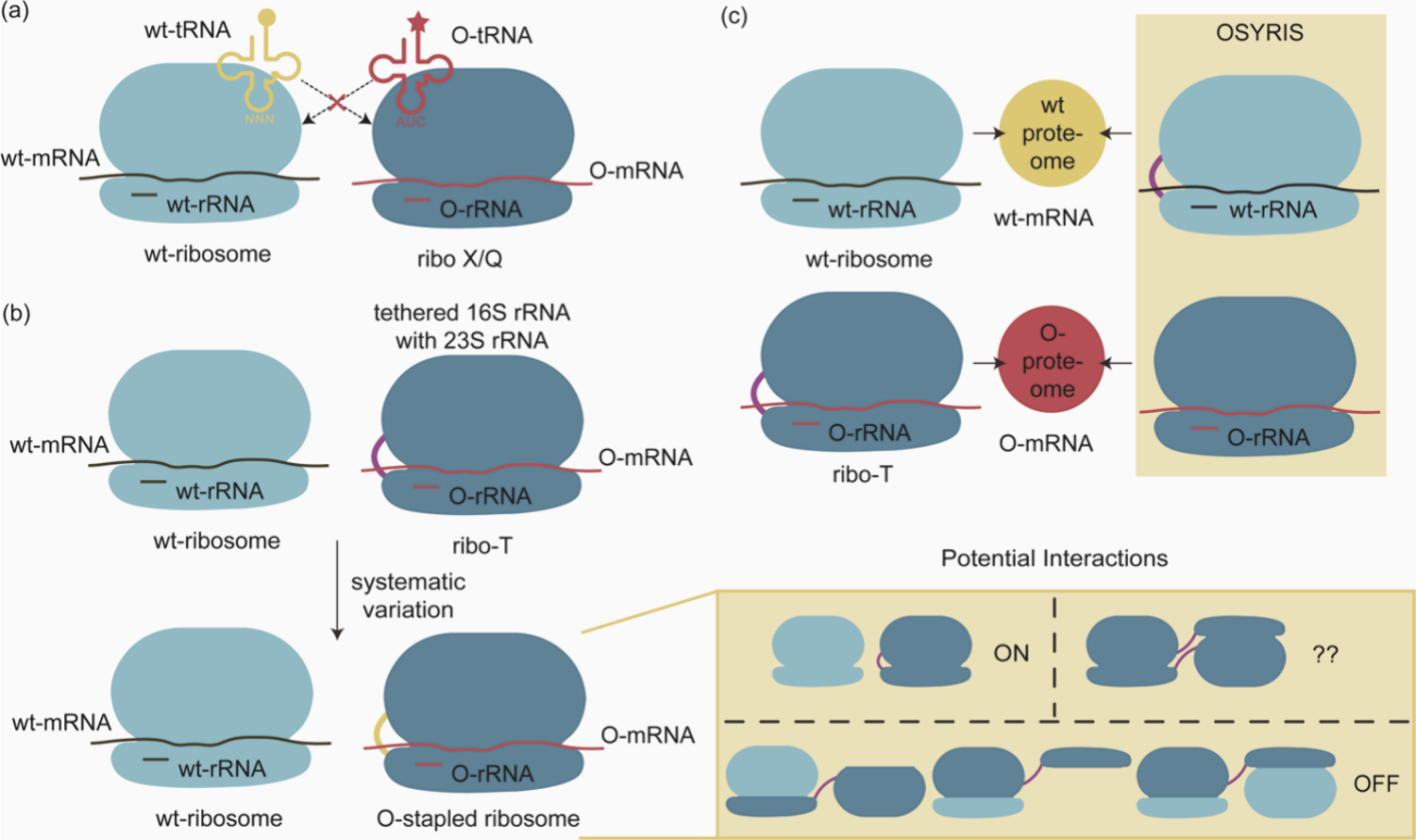
Engineering of ribosomes. (a) O-Ribosome and O-mRNA pairs such as ribo-X and ribo-Q consist of an mRNA containing a ribosome-binding site that does not direct translation by the endogenous ribosome, coupled with an orthogonal ribosome capable of efficiently and specifically translating the orthogonal mRNA. (b) In ribo-T and O-stapled ribosome, short RNA linkers integrate sequences from both small and large subunit rRNA. (c) In OSYRIS system, orthogonal proteome was translated by ribosomes composed of the dissociable orthogonal 30S subunit and the wild-type 50S subunit. Meanwhile, the endogenous cellular proteome was translated by ribosomes with tethered subunits. Abbreviations: O-tRNA, orthogonal tRNA; O-mRNA, orthogonal mRNA; O-proteome, orthogonal proteome; OSYRIS, orthogonal translation system based on ribosomes with isolated subunits.

In 2005, Rackham et al.^141^ introduced the concept of orthogonal ribosome (O-ribosome) and orthogonal mRNA (O-mRNA) pairs. These pairs consist of an mRNA containing a ribosome-binding site that does not direct translation by the endogenous ribosome, coupled with an orthogonal ribosome capable of efficiently and specifically translating the orthogonal mRNA. Importantly, these orthogonal ribosomes do not appreciably translate cellular mRNAs. Subsequently, Wang et al.^142^ advanced the field by evolving orthogonal ribosomes, resulting in ribo-X (Figure 2a). Notably, ribo-X exhibited reduced recognition of RF1 and increased efficiency in orthogonal tRNA-dependent amber suppression. Building upon this foundational research, Neumann et al.^131^ furthered the development of orthogonal ribosomes for enhanced quadruplet decoding in 2010. Recognizing that natural ribosomes are inherently inefficient and nonadaptive for quadruplet decoding, which could lead to proteome-wide misreading, they undertook the creation of saturation mutagenesis libraries within the 16S rRNA (rRNA) of ribo-X. This effort yielded four O-ribosome mutants collectively referred to as ribo-Q. These ribo-Q mutants, incorporating two or three specific mutations, exhibited the capability to efficiently decode a range of quadruplet codons, approaching the efficiency observed for triplet decoding. Ribosome-Q has subsequently been applied to incorporate more than ten distinct ncAAs^28,136^ into proteins.

In addition to the significant role of 16S rRNA, the 23S rRNA in the large ribosomal subunit assumes critical functions in mediating interactions with tRNAs, elongation factors, peptide bond formation within the peptidyl-transferase center (PTC), and the facilitation of the folding and release of the nascent polypeptide chain through the exit tunnel. However, the inherent interactions between ribosomal subunits have historically limited the scope of orthogonal ribosome engineering endeavors. In 2015, Orelle et al.^143^ addressed this formidable challenge by pioneering a revolutionary approach, a new hybrid rRNA structure that seamlessly integrates sequences from both small and large subunit rRNA. This complex integration was achieved through the careful use of short RNA linkers, leading to the development of ribosomes with linked subunits that are effectively inseparable. These engineered ribosomes, termed Ribo-T (Figure 2b), have demonstrated their functional competence by efficient protein synthesis.

However, it is worth noting that researchers observed slower growth rates in Ribo-T cells compared to those with wild-type ribosomes, primarily attributed to delayed Ribo-T assembly.^144,145^ In response to this limitation, Carlson et al.^146^ adopted an evolutionary approach to select new tether designs capable of supporting faster cell growth and increased protein expression. Furthermore, they successfully evolved new orthogonal Ribo-T/mRNA pairs that operate in parallel with, yet independently of, natural ribosomes and mRNAs, substantially enhancing the efficiency of orthogonal protein expression.

Kim et al.^147^ subsequently introduced a versatile method guided by computational design principles for the directed evolution of sequence-distant sites within macromolecular machines, which they named Evolink (evolution and linkage) in 2022. This innovative approach comprises a three-step process involving PCR, ligation, and a subsequent PCR step, facilitating the physical assembly of formerly separated regions of a plasmid into a continuous next-generation sequencing (NGS) read. The design of the tethered ribosome library was guided by both NGS results and computational RNA structural modeling. By employing the Evolink method, the researchers successfully engineered a novel tethered ribosome featuring previously unexplored tether RNA sequences, designated as Ribo-T v3. This advanced ribosome variant demonstrated a remarkable 58% increase in activity in orthogonal protein translation and successfully improved the incorporation efficiency of the noncanonical l-α-amino acid ((*R*)-2-amino-3-(7-(diethylamino)-2-oxo-2*H*-chromene-3-carboxamido)propanoic acid, DECP) with the help of flexizyme charging DECP onto tRNA^fMet^_CAU_. Furthermore, it exhibited an extraordinary 97% improvement in cell doubling times when compared to a previously developed tethered ribosome.

In 2020, Aleksashin et al.^148^ introduced a novel system characterized by two functionally independent translation machineries by reversing the roles of Ribo-T and the endogenous dissociable ribosomes. This innovative configuration features one translation apparatus comprising dissociable yet fully segregated ribosomes to translate orthogonal mRNAs. These ribosomes are composed of the dissociable orthogonal 30S (o-30S) subunit and the wild-type (wt) 50S subunit, functioning as a fully orthogonal translation machine. Meanwhile, the endogenous cellular proteome was translated by ribosomes with tethered subunits. This groundbreaking system, aptly named OSYRIS (orthogonal translation system based on ribosomes with isolated subunits), attains complete orthogonality due to the physically unlinked o-30S and 50S ribosomal subunits being compelled to interact with each other and function as fully orthogonal ribosomes (Figure 2c). Researchers have also identified rRNA mutations that could overcome ribosome stalling at stop codon. This configuration offers remarkable versatility, allowing not only the o-30S subunit but also the free 50S subunit to be engineered to acquire new functionalities without causing disruptions in the expression of the cellular proteome, which might lead to incorporation of ncAAs that the endogenous ribosome discriminated against (such as d- and β-amino acids) in the future.

In a manner reminiscent of the Ribo-T approach, Fried et al.^149^ effectively employed rational design to craft a synthetic rRNA. This engineered RNA was purposefully constructed to establish covalent connections between the large and small ribosomal subunits using an RNA staple. This engineering endeavor yielded artificial ribosomes with tethered subunits precisely directed toward an orthogonal mRNA. This design not only facilitated the deliberate introduction of mutations into the large subunit but also prevented unwanted interactions with native large or small ribosomal subunits. Consequently, this strategic approach mitigated any potential adverse effects on cellular growth. Subsequently, Schmied et al.^150^ further advanced this concept by developing optimized orthogonal stapled ribosomes (referred to as O-stapled ribosomes, Figure 2b). These ribosomes demonstrated remarkable efficiency in polymerizing a sequence of monomers that the natural ribosome is inherently unable to translate, such as proline-rich sequences in the absence of elongation factor P (EF-P). Moreover, the researchers employed cryo-electron microscopy to visualize the structural aspects of these O-stapled ribosomes, shedding light on the mechanism by which the staple linkage governs the association between ribosomal subunits.

Recently, Liu et al.^151^ applied phage-assisted continuous evolution (PACE) to orthogonal ribosome in 2021, developing the oRibo-PACE methodology. They developed a PACE-compatible orthogonal translation system and 16S rRNAs from *E. coli*, *Pseudomonas aeruginosa*, and *Vibrio cholerae* evolved toward enhanced protein synthesis rates. After continuous directed evolution of orthogonal ribosomes, the resulting orthogonal rRNAs (o-rRNAs) showed augmented translation efficiencies over starting *E. coli* rRNA and successfully incorporated l-azidohomoalanine (AHA) into proteins.

Furthermore, Dunkelmann et al.^137^ transformed the design process of 5′ UTR sequences for O-mRNA by implementing a thermodynamic model of initiation with a simulated annealing optimization algorithm. This approach led to the identification of sequences capable of achieving a remarkable 40-fold increase in protein production while concurrently realized up to a 50-fold enhancement in orthogonality when compared to previous O-mRNAs. They successfully established a 68-codon, 24-amino-acid genetic code, enabling the proficient incorporation of four distinct ncAAs in response to four distinct quadruplet codons.

#### 3.3.5 Engineering of the NMD Pathway

Eukaryotic cells possess a mRNA surveillance mechanism known as nonsense-mediated mRNA decay (NMD), which is essential for identifying mRNAs containing premature stop codons and subsequently directing them toward rapid degradation. The utilization of the amber stop codon for encoding ncAAs exposes the mRNA to NMD, resulting in a consequential reduction in protein yield. In 2008, Wang et al.^152^ executed a groundbreaking development by generating an NMD-deficient yeast strain through the deletion of its *Upf1* gene, a well-established essential component in the NMD pathway. This innovative strain notably exhibited a substantial enhancement in the efficiency of ncAA incorporation when compared to the wild-type yeast, thereby enabling the incorporation of ncAAs into proteins at significantly elevated yields, reaching tens of milligrams per liter. Building upon this success, a similar strategy was employed by Parrish et al.^153^ in 2012 in the context of *C. elegans*. Through the utilization of RNA interference (RNAi), they effectively knocked down the NMD component smg-1 in *C. elegans*, resulting in a noteworthy increase in the efficiency of ncAA incorporation within the nematode, further demonstrating the utility of NMD inactivation to enhance ncAA incorporation.

#### 3.3.6 Engineering of Surrounding Sequence Context

Due to different sequence context surrounding UAG codons, considerable variations in the rates of ncAA incorporation are frequently observed in both bacterial and mammalian cells. While the precise influence of cis-acting sequence elements on amber suppression and ncAA incorporation rates within an expanded genetic code remains largely elusive, extensive research endeavors have been undertaken to elucidate this aspect. For instance, Bartoschek et al.^154^ developed the identification of permissive amber sites for suppression (iPASS) tool to predict relative ncAA incorporation efficiencies based on the surrounding sequence context. They optimized ncAA incorporation efficiencies by implementing synonymous codon substitutions flanking the amber stop codon, guided by iPASS and achieved 1.4 to 5.3-fold of incorporation efficiency increase in HEK293T cells. In 2022, Chen et al.^155^ adopted another context engineering strategy by leveraging recoding signals embedded within mRNA to significantly enhance the decoding efficiency of quadruplet UAGN and AGGN codons. The expression level of proteins encoding ncAAs with UAGN and AGGN codons has reached 48% and 98% of the wild-type protein, respectively.

#### 3.3.7 Advancements in GCE Technology through Phase Separation

**Figure 3:**
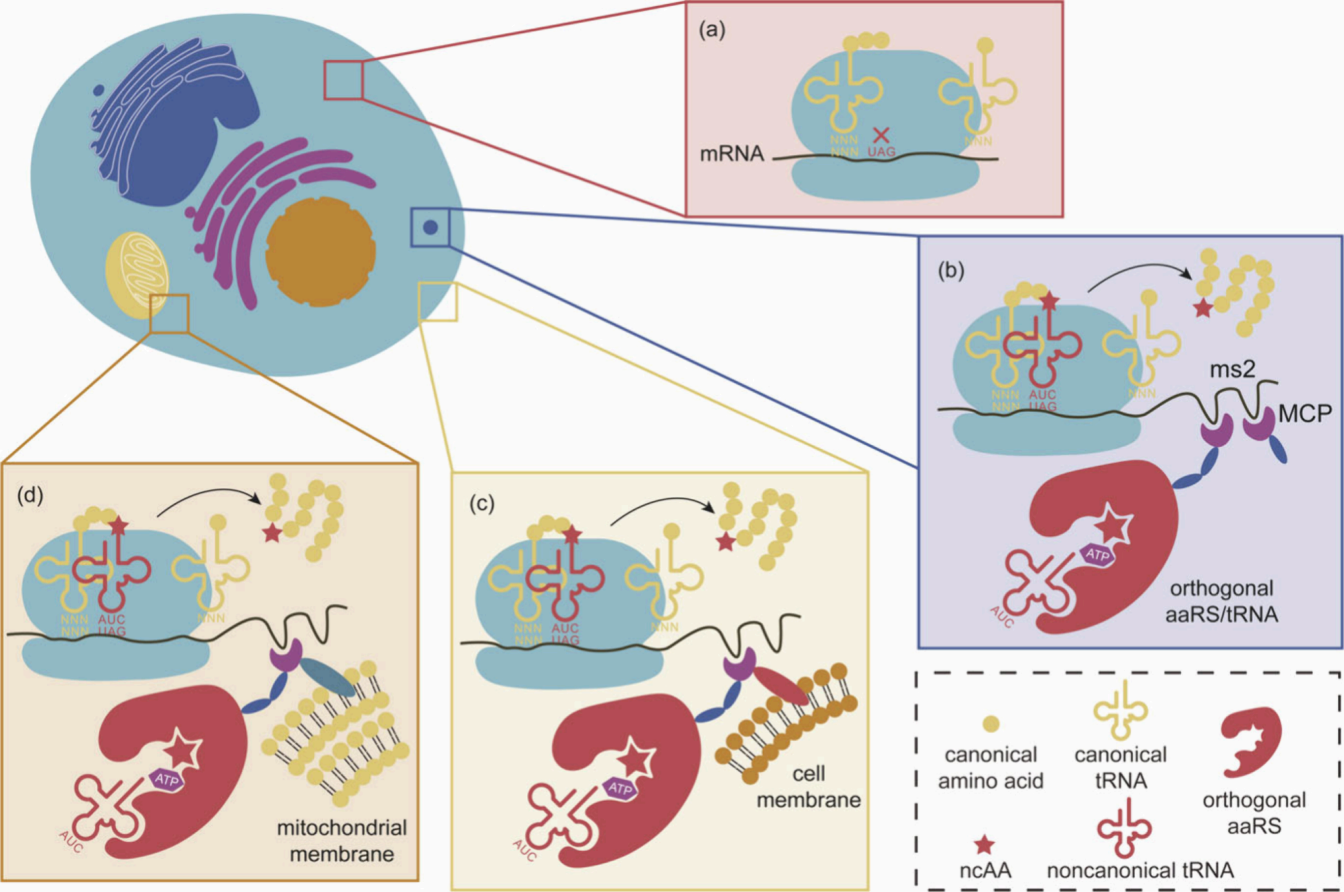
Applications of phase separation in GCE. (a) Traditional GCE technology. (b) Designer membraneless organelles enable codon reassignment of selected mRNAs in eukaryotic cytoplasm. (c) Spatial separation of orthogonal eukaryotic translation on cell membrane. (d) Spatial separation of orthogonal eukaryotic translation on mitochondrial membrane. Abbreviations: MCP, major capsid protein.

Recent progress has unveiled the significance of phase separation as a prevailing mechanism facilitating the localized concentration of biomacromolecules, including proteins and RNAs. Consequently, researchers have leveraged phase separation to realize completely orthogonal GCE within eukaryotic systems. In 2019, Reinkemeier et al.^156^ pioneered the development of an orthogonal translation system through the spatial enrichment of key components of the GCE machinery within an orthogonally translating (OT) synthetic designer organelle, leading to site-specific ncAA incorporation only into targeted protein mRNAs (Figure 3).

This OT synthetic designer organelle consists of three integral components: (1) an mRNA encoding the protein of interest (POI), fused to two ms2 RNA stem loops, which possess specific binding affinity to the phage-derived major capsid protein (MCP), (2) an intermediary molecule containing MCP and an additional domain capable of forming substantial aggregates with engineered aaRSs fused to specific domains, and (3) aaRS/tRNA pairs capable of initiating phase separation upon interaction with the intermediary molecule. This orchestrated interplay brings the mRNA into proximity with the aaRS/tRNA pair, thereby minimizing the inadvertent incorporation of ncAAs into other mRNAs. Consequently, they achieved precise site-specific incorporation of ncAAs exclusively into the protein of interest, while other cytoplasmic mRNAs containing the same stop codon experienced diminished translation efficiency, thereby accomplishing mRNA selectivity.

Following this, in 2021, Reinkemeier et al.^157^ harnessed 2D phase separation methodologies to engineer orthogonal aaRSs, resulting in the establishment of numerous film-like biochemical microenvironments across various membrane surfaces. This groundbreaking approach facilitated the development of multiple OT film-like organelles, each capable of supporting distinct spatially orthogonal aaRS/tRNA pairs within the same cellular cytoplasm. Consequently, they could reutilize the same stop codon to introduce a diverse array of ncAAs into different proteins in vivo, thereby conferring upon the cell three spatially and functionally distinct translational programs.

#### 3.3.8 Engineering of Host Strain Pathways

As previously mentioned, researchers have eradicated the ncAA-encoding codon from the host genome or utilized more resilient host species capable of proficiently expressing proteins, exemplified by *V. natriegens*(56) to bolster GCE efficiency. Furthermore, it is imperative to acknowledge that GCE efficiency within mammalian cells can be influenced by a multitude of factors. Stress conditions within mammalian cells have the potential to diminish GCE efficiency by triggering the activation of the protein kinase R (PKR)-dependent eIF2α phosphorylation pathway. The phosphorylated eIF2α subsequently acts as an inhibitor of eIF2B, culminating in the inhibition of translation initiation and a global reduction in translation rates. In response to this challenge, Sushkin et al.^158^ devised several strategies to remodel the cellular stress response. One approach involves increasing the activity of the eIF2B complex by adding catalytic subunits, thereby enhancing the guanine nucleotide exchange factor (GEF) activity and increasing the abundance of eIF2•GTP complexes. Another strategy focuses on reducing PKR autophosphorylation and subsequent eIF2α phosphorylation by introducing the N-terminal PKR fragment, which ultimately leads to increased reporter protein expression. Additionally, a third strategy aims to mutate eIF2α at the S51A site, erasing the phosphorylation site and allowing it to outcompete endogenous eIF2α in potential phosphorylation reactions, thus lowering the concentration of phosphorylated eIF2α. These strategies have been shown to significantly increase GCE efficiency in mammalian cells.

### 3.4 Optimization of Screening Methods for Selection of Orthogonal aaRS/tRNA Pairs

GCE requires the use of orthogonal aaRSs that specifically recognize corresponding ncAAs. This necessitates screening libraries for suitable aaRS and tRNA pairs. Typically, this screening involves molecular docking to analyze the crystal structures of aaRS and the target ncAA, followed by mutating multiple sites within the aaRS’s amino acid binding pocket to create a mutation library. This library is then subjected to thorough selection or screening processes. Notably, this method is labor intensive and time-consuming. Researchers have utilized diverse approaches to streamline the processes of library construction and screening. For instance, Amiram et al.^159^ established a genomically integrated aaRS library through multiplex automated genome engineering (MAGE), allowing for an increase in genetic diversity by simply expanding the number of MAGE cycles. Recently, Jason Chin’s lab^160^ has also developed a novel technique, tRNA display, facilitating the immediate, efficient, and scalable selection of orthogonal synthetases, which are adept at acylating their specific orthogonal tRNAs with noncanonical monomers (ncMs) in *E. coli*, regardless of the ncMs’ suitability as ribosomal substrates. Additionally, fluorescence-activated cell sorting (FACS) has been implemented in aaRS screening,^161−166^ which has reduced the need for extensive manual validation.

**Figure 4:**
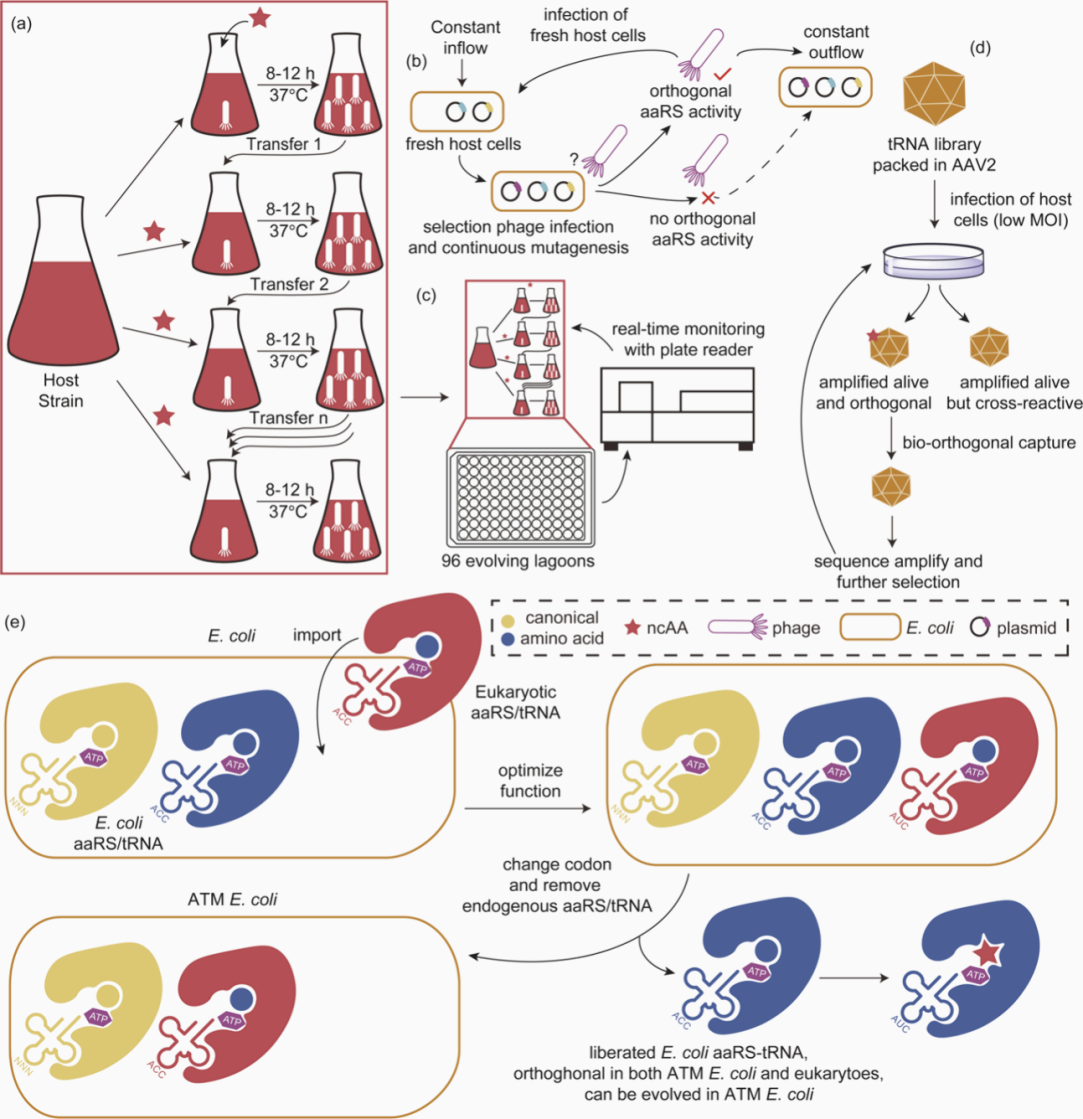
Optimization of screening methods. (a) Principle of phage-assisted noncontinuous evolution (PANCE). (b) Principle of phage-assisted continuous evolution (PACE). (c) Principle of phage- and robotics-assisted near-continuous evolution (PRANCE). (d) Principle of virus-assisted directed evolution of tRNAs (VADER). (e) In ATM *E. coli* strain, the native *Ec*TrpRS/tRNA pair was replaced with an *E. coli*, optimized counterpart from *S. cerevisiae*. Abbreviations: AAV, adeno-associated virus; ncAA, noncanonical amino acid; ATM, altered translational machinery.

Nevertheless, the screening procedures continue to be rather labor-intensive. Thus, scientists have invented series of easier screening strategies. In the following section, we will introduce several innovative screening methods for aaRS and tRNA pairs. First, we will explore the phage-assisted noncontinuous evolution (PANCE, Figure 4a), PACE (Figure 4b), and phage- and robotics-assisted near-continuous evolution (PRANCE, Figure 4c) techniques, which optimize laboratory evolution processes, significantly enhancing the catalytic efficiency and substrate specificity of aaRSs. Next, we will describe the virus-assisted directed evolution of tRNAs (VADER, Figure 4d) technique, which achieves virus-assisted directed evolution of tRNAs in mammalian cells, overcoming the limitations of traditional methods in these environments. Finally, we will discuss the screening and directed evolution of EcaaRS/tRNA pairs in *E. coli*, enabling precise incorporation of ncAAs in both bacterial and mammalian cells.

#### 3.4.1 PANCE, PACE, and PRANCE

The laboratory evolution of aaRS with modified amino acid specificity usually involves three to five rounds of both positive and negative selections. Due to this limited number of selection rounds applied to focused libraries, there’s often a recurring emergence of suboptimal characteristics in the lab-evolved aaRSs. These properties may include an approximate reduction in activity (*k*_cat_/*K*_M_) and selectivity for the target ncAA.^167^ In 2017, Suzuki et al.^77^ introduced an innovative laboratory evolution technique named PANCE. This streamlined method facilitates rapid in vivo directed evolution through serial flask transfers using standard laboratory equipment. Importantly, PANCE eliminates the prerequisite for prior structural knowledge in protein evolution. In this approach, *M. mazei* tRNA^Pyl^ and a phage essential gene containing the UAG stop codon, *gIII*, were incorporated into an accessory plasmid (AP) within the host *E. coli* and the *gIII* gene of selection phage (SP) was replaced with the target aaRS gene for evolution. Subsequently, serial transfer of the SP across fresh *E. coli* host cells allows for the continuous evolution of the aaRS across successive generations within the *E. coli* population. This iterative process resulted in a remarkable increase in catalytic efficiency of PylRS, with improvements of up to 10-fold, coupled with enhanced substrate specificity. PANCE has subsequently found application in the evolution of other aaRSs, including *Ma*PylRS.^168^

Similarly, Bryson et al.^169^ introduced a previously developed technique known as PACE to expedite the rapid generation of highly active and selective orthogonal aaRSs over hundreds of evolutionary generations. In contrast to the PANCE approach, the PACE experimental setup necessitates the utilization of continuous flow machinery, entailing additional efforts for establishment. However, PACE effectively integrates positive and negative selections concurrently, thereby obviating the requirement for repetitive plasmid isolation and retransformation. This streamlining of the laboratory evolution process for orthogonal aaRSs spans numerous generational iterations, encompassing mutation, selection, and replication, ultimately reducing the duration of experiments.^170^ Two distinct strategies were devised to establish a functional link between aaRS activity and phage infectivity through amber suppression of premature stop codons in either the T7 RNAP or the *gIII* gene. By harnessing the power of PACE, scientists achieved a remarkable enhancement in the catalytic efficiency of PylRS, with improvements of up to 45-fold compared to the parent enzyme. Furthermore, they successfully evolved a promiscuous mutant of *Mj*TyrRS into a variant exhibiting over 23-fold higher selectivity for its substrate ncAA.

Subsequently, DeBenedictis et al.^95^ introduced PACE for quadruplet-decoding tRNA evolution, referred to as qtRNA-PACE. This approach was devised to facilitate the discovery and characterization of amino acid-specific qtRNAs, which led to a substantial enhancement in quadruplet codon translation efficiencies, achieving up to 80-fold improvement. These results were particularly noteworthy as qtRNA-PACE demonstrated comparable performance to traditional amber suppression in cellular translation assays. Moreover, it enabled the processive translation of six adjacent quadruplet codons. Furthermore, qtRNA-PACE yielded a set of four mutually orthogonal qtRNA–quadruplet codon pairs, which could function collectively. This research could potentially be the foundation for the novel synthesis of quadruplet codon/qtRNA pairs corresponding to each of the 20 canonical amino acids.

In 2021, DeBenedictis et al.^93^ further introduced a robotic system known as PRANCE, which combines a high-throughput plate-based methodology with PACE. PRANCE houses evolving bacteriophage populations in 96-well plates, managed by a high-throughput liquid-handling system. Controlled via Python scripting, it mimics the flow rates of host bacteria seen in continuous-flow bioreactors, ensuring precise and efficient evolution processes. PRANCE’s exceptional scalability, its capacity to handle multiple parallel populations, historical data retention in the convenient 96-well plate format, real-time monitoring of molecular fitness parameters, and its precise temporal and chemical environmental control establish it as a choice when compared to alternative screening methods. PRANCE has been employed to concurrently evolve qtRNAs, exposing a complete set of 20 distinct paralogues to evolutionary processes within a single experiment. This achievement circumvented the need for multiple individual PACE experiments.

#### 3.4.2 VADER

In a significant milestone, Jewel et al.^94^ accomplished VADER in mammalian cells during the year 2022. It addresses a critical gap in the field since no suitable selection systems were available for achieving directed evolution of aaRS/tRNA pairs in mammalian cellular environments. Traditional directed evolution strategies for tRNA screening face inherent challenges when applied to mammalian cells for two primary reasons.^15^ First, to maintain a clear genotype–phenotype connection, it is imperative to restrict the integration of mutant tRNA genes within a single cell to no more than one. However, it has been empirically demonstrated that achieving detectable ncAA incorporation efficiency necessitates a substantial number of tRNA genes, exceeding 100, per cell. This creates a practical limitation. Second, these strategies are inherently characterized by a low mutagenic frequency, rendering them ill-suited for the evolution of tRNAs, given their relatively small size, typically less than 100 base pairs.

Jewel et al. addressed these formidable challenges by establishing a sophisticated system that intricately linked the activity of suppressor tRNA to the replication of adeno-associated virus 2 (AAV2) within mammalian cells. In this carefully designed framework, mammalian cells were subjected to infection with AAV2 vectors harboring the tRNA library, administered at a low multiplicity of infection (MOI). Complementary plasmids encoding the TAG mutant of Cap, along with other requisite genetic components necessary for AAV replication and the cognate aaRS, were introduced in trans through transfection. The active and orthogonal tRNA mutants facilitated the production of packaged progeny AAV2 that incorporated the desired ncAA into their capsid proteins. These engineered viruses were subsequently isolated employing chemoselective biotin conjugation, followed by streptavidin-mediated pulldown. This pioneering approach yielded successful improvements in mutants of *Mm*tRNA^Pyl^ and *Ec*tRNA^Tyr^.

#### 3.4.3 Screening of *Ec*aaRS/tRNA Pairs in *E. coli*

Owing to the requisite for orthogonality, over recent decades, the genetic incorporation of novel ncAAs into eukaryotic organisms has predominantly relied upon the unique PylRS/tRNA pairs, given their compatibility with engineering within the *E. coli* selection system, subsequently enabling their application in eukaryotic contexts. In 2017, Italia et al.^42^ introduced a method to screen *Ec*TrpRS/tRNA pairs in *E. coli* while preserving the native tryptophan incorporation process. This was achieved through genetic engineering of an *E. coli* strain named altered translational machinery (ATM), where the native *Ec*TrpRS/tRNA pair was replaced with an *E. coli*, optimized counterpart from *S. cerevisiae*. Later, in 2018, Italia et al.^171^ created a strain named ATMY with same strategy: the native *Ec*TyrRS/tRNA pair was replaced with the archaea-derived *Mj*TyRS/tRNA pair. Subsequently, they reintroduced the amber variant of the *Ec*TyrRS/tRNA pair, successfully enabling site-specific incorporation of 3-iodo-l-tyrosine (3iY) and 3-azido-l-tyrosine.

Building on this work, Italia et al.^171^ conducted similar experiments and screened mutants of *Ec*TyrRS/tRNA pairs within the ATMY strain. They successfully identified *Ec*TyrRS variants capable of recognizing ncAAs such as *p*-boronophenylalanine (pBoF)^171^ and *O*-sulfotyrosine^172^ in mammalian cells. In 2022, Grasso et al.^173^ further improved this system through the directed evolution of tRNA_CUA_^*Ec*Tyr^, eliminating its cross-reactivity with the endogenous glutaminyl-tRNA synthetase (Gln *R*S). Additionally, they developed a straightforward survival-based dynamic range (WiDR) antibiotic selection method capable of distinguishing between highly and weakly active *Ec*TyrRS mutants. This approach enhanced the performance of the initially underperforming first-generation *Ec*TyrRS and led to the creation of new mutants. These mutants efficiently incorporate various novel ncAAs, such as *p*-benzoylphenylalanine (pBPA), 2-amino-3-(4-(2-azidoacetamido)phenyl)propanoic acid (pAAF) and *p*-carboxymethylphenylalanine (pCMF).

In 2017, Italia et al.^42^ employed a similar strategy to screen *Ec*TrpRS/tRNA pairs within *E. coli*. They introduced the *S. cerevisiae*-derived *Sc*TrpRS/tRNA pair into the *E. coli* host to substitute the endogenous *Ec*TrpRS/tRNA pair. Within this specialized *E. coli* strain, polyspecific *Ec*TrpRS/tRNA pairs compatible with various 5-substituted tryptophan derivatives such as 5-hydroxytryptophan (5HTP), (*S*)-2-amino-3-(5-methoxy-1*H*-indol-3-yl)propanoic acid (5MTP), (*S*)-2-amino-3-(5-azido-1*H*-indol-3-yl)propanoic acid (5AzW), and (*S*)-2-amino-3-(5-amino-1*H*-indol-3-yl)propanoic acid (5AmW) were developed from a library of *Ec*TrpRS variants. This evolved *Ec*TrpRS/tRNA pair was subsequently introduced into mammalian cells, where its orthogonality was confirmed, allowing for the site-specific incorporation of tryptophan derivatives within mammalian cellular contexts.

### 3.5 Biosynthesis of ncAAs

GCE has achieved a significant milestone by enabling the incorporation of several hundred ncAAs into proteins to date. Unlike endogenously synthesized natural amino acids, the incorporation of ncAAs in GCE predominantly relies on their external addition during protein synthesis, originating from chemical synthesis. The synthesis process, fraught with inherent technical complexities, notably inflates production costs. Thus, biosynthesis of ncAAs emerges as a promising alternative.

In nature, some ncAAs such as selenocysteine and pyrrolysine have their own pathways for incorporation into the proteome. Selenocysteine’s biosynthesis, unlike conventional amino acids, involves a complex multistep enzymatic process, reliant on specialized aaRS for its tRNA linkage, with bacterial systems utilizing SerRS and selenocysteine synthase (SelA),^174^ while eukaryotic and archaeal systems add layers of complexity with SerRS, *O*-phosphoseryl-tRNA kinase (PSTK), and phosphoseryl-tRNA:selenocysteinyl-tRNA synthase (SepSecS).^175^

**Figure 5:**
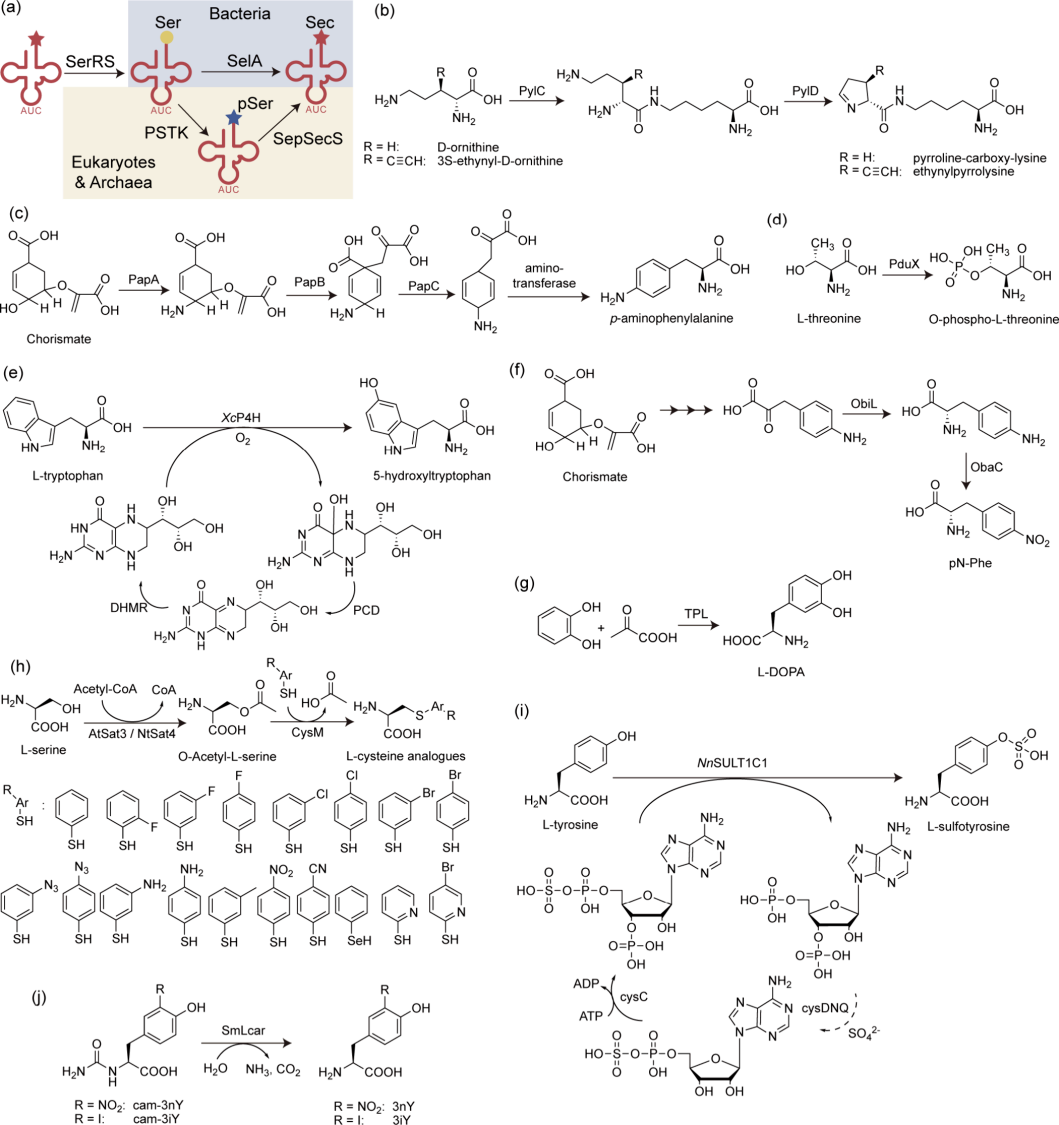
Biosynthesis of ncAAs. (a) Biosynthesis of selenocysteine in bacteria, eukaryotes, and archaea. (b) Biosynthesis of pyrrolysine and derivatives. (c) Biosynthesis of pAF. (d) Biosynthesis of pThr. (e) Biosynthesis of 5-HTP. (f) Biosynthesis of pN-Phe. (g) Biosynthesis of DOPA. (h) Biosynthesis of ncAAs based on cysteine biosynthetic pathway. (i) Biosynthesis of sTyr. (j) Biosynthesis of 3nY and 3iY. Abbreviations: PSTK, *O*-phosphoseryl-tRNA kinase; *Xc*, *Xanthomonas campestris*; P4H, phenylalanine 4-hydroxylase; DHMR, dihydromonapterin reductase; PCD, pterin-4α-carbinolamine dehydratase; TPL, tyrosine phenol-lyase; 3nY, 3-nitro-l-tyrosine; 3iY, 3-iodo-l-tyrosine; *Nn*, *Nipponia nippon*.

Meanwhile, the pyrrolysine biosynthetic pathway transform two lysine molecules into a single pyrrolysine through the *pylTSBCD* gene cluster in archaea (Figure 5a). Notably, the heterologous introduction of this cluster into *E. coli* by David G. Longstaff et al.^176^ and subsequent work by Yuda Chen et al.^177^ underscored challenges, including cytotoxic effects. Addressing this, in 2021, Joanne M. L. Ho et al.^178^ implemented alternating phage assisted non-continuous evolution (Alt-PANCE) on this biosynthetic pathway, which resulted in a substantial 32-fold increase in protein production incorporating pyrrolysine.

In addition to the naturally existed biosynthetic pathways for these two ncAAs, significant advancements have been made in the development of novel biosynthetic routes capable of de novo synthesis of other ncAAs which can be incorporated into protein. A landmark achievement in this field was realized in 2003 by Ryan A. Mehl et al.,^179^ who pioneered the use of de novo biosynthesized ncAAs in GCE (Figure 5c). Their innovative approach involved leveraging enzymes PapA, PapB, and PapC from *Streptomyces venezuelae*, in conjunction with *E. coli* transaminase. Utilizing chorismate, a key biosynthetic intermediate in the synthesis of aromatic amino acids in *E. coli*, they successfully synthesized *p*-aminophenylalanine (pAF).

In 2017, Michael Shaofei Zhang et al.^180^ successfully engineered a comprehensive biosynthetic pathway for the integration of phosphothreonine (pThr) into proteins (Figure 5d). Prior attempts at elevating intracellular pThr levels, even with the supplementation of 1 mM pThr in the culture medium, faced limitations due to the molecule’s negative charge, which hinders its efficient uptake by cells. The team drew inspiration from the metabolic processes of *Salmonella enterica*, in which a kinase named PduX catalyzes the conversion of l-threonine to pThr. Remarkably, this biosynthetic strategy led to a 40-fold increase in intracellular phosphothreonine (pThr) concentration compared to the levels attained through exogenous feeding of 1 mM pThr, achieving the synthesis of pThr at millimolar concentrations within the bacterial system.

In another study in 2020, Yuda Chen et al.^181^ engineered a self-sustaining biological system capable of utilizing 5-HTP as an additional amino acid, effectively expanding the genetic code to include this 21st amino acid (Figure 5e). Known primarily as a precursor to serotonin in humans, 5-HTP’s integration into *E. coli* proteins was achieved after the team conducted extensive testing of hydroxylases from various organisms, leading to the successful synthesis and integration of 5-HTP into proteins within *E. coli*. Continuing this trajectory of innovation, Neil D. Butler et al.^182^ achieved the biosynthesis of *p*-nitrophenylalanine (pN-Phe) in *E. coli* in 2023 (Figure 5f). Their methodology began with the metabolic synthesis of the amine precursor pAF utilizing the natural gene cluster *papABC*, followed by the nonheme diiron *N*-monooxygenase enzyme ObiL, derived from *Pseudomonas fluorescens*. This novel biosynthetic route facilitated the accumulation of pN-Phe to near-millimolar concentrations within the *E. coli* cells, thereby enabling its incorporation into proteins via GCE.

Semisynthetic pathways for the production of ncAAs have also been developed. Utilizing the natural biosynthetic pathway of pyrrolysine as a template, several semisynthetic analogues of pyrrolysine have been synthesized (Figure 5b). A notable achievement in this area was made in 2011 by Weijia Ou et al.,^183^ who successfully biosynthesized pyrroline-carboxy-lysine (Pcl) by supplementing the culture medium with d-ornithine. Pcl has the unique ability to be recognized and integrated into proteins by PylRS. In a similar vein, in 2015, Michael Ehrlich et al.^184^ introduced 3*S*-ethynyl-d-ornithine into the culture medium, leading to the production of ethynylpyrrolysine (ePyl). ePyl is also amenable to site-specific incorporation into proteins through the PylRS/tRNA system. In 2018, Sanggil Kim et al.^185^ endeavored to synthesize l-dihydroxyphenylalanine (DOPA), a naturally occurring compound in the catecholamine biosynthesis of animals and plants (Figure 5g). To achieve this, tyrosine phenol-lyases (TPLs) were selected as the enzymatic catalyst for the synthesis of DOPA from catechol. By supplying high concentrations of catechol in the culture medium, they accomplished efficient synthesis of DOPA and its site-specific incorporation into proteins.

Beyond biosynthetic pathways designed for the synthesis of specific or a few ncAAs, researchers have developed more universal approaches to synthesize a broader array of amino acids. In a foundational study conducted in 2003, Thomas H. P. Maier^186^ utilized the cysteine biosynthetic pathway in *E. coli* to semsynthesize a variety of ncAAs. By providing exogenous sulfur donors, the *E. coli* endogenous *O*-acetylserine sulfhydrylase can transform acetylserine and sulfate into different ncAAs, thanks to its broad substrate specificity. This pathway was also utilized by Schipp et al.^187^ to semisynthesize l-azidohomoalanine (Aha) in *E. coli* and insert Aha into target proteins through selective pressure incorporation (SPI). Additionally, building on Maier’s work, Matthias P. Exner et al.^188^ achieved the synthesis of S-allyl-l-cysteine (Sac) in *E. coli* through the addition of allyl mercaptan, leveraging a specially engineered PylRS for protein incorporation. And later, Yong Wang et al.,^11^ in 2021, biosynthesized nearly 50 distinct ncAAs in *E. coli* (Figure 5h). This was achieved by administering a range of economically feasible, readily available, or easily synthesized aromatic thiol precursors. The spectrum of ncAAs synthesized included molecules featuring biologically orthogonal reaction groups, such as phenylamine, aromatic ketone, and aromatic azides. This methodology markedly lowered the production costs and synthetic complexities traditionally associated with generating recombinant proteins containing ncAAs in standard biology laboratory. Representing a cost-effective, efficient, and broadly applicable approach, this strategy significantly advances the capabilities for synthesizing and genetically incorporating diverse ncAAs into recombinant proteins.

Researchers have successfully biosynthesized and site-specific incorporated ncAAs in both *E. coli* and eukaryotic cells. In a notable study conducted in 2022, Yuda Chen et al.^189^ elucidated mechanisms for both the biosynthesis of sulfotyrosine (sTyr) and its precise integration into proteins within both bacterial and mammalian cell systems (Figure 5i). They harnessed a key enzyme, tyrosine sulfotransferase SULT1C1 from *Nipponia nippon*, identified through a sequence similarity network (SSN), which is essential for the sTyr biosynthetic pathway. Cells using this pathway for producing proteins with site-specific sulfation showed a higher yield compared to those relying on externally added sTyr at concentrations between 3 and 27 mM.

Advancing beyond the mere adoption of biosynthetic pathways from diverse species, the scientific community is increasingly applying directed evolution to enhance and tailor these pathways. A notable instance of this approach was demonstrated in 2022 by Rudy Rubini et al.,^190^ who linked the biosynthesis and site-specific incorporation of ncAAs to the growth dynamics of *E. coli*. Through continuous passage, they efficiently selected more effective variants of SmLcar, a crucial carbamoylase from a large enzyme library. This enzyme is key in catalyzing the hydrolysis of *N*-carbamoyl-amino acids, enhancing the production of the ncAA precursor, *N*-carbamoyl-l-3nY (cam-3nY), and facilitating the biosynthesis and accurate integration of 3nY into proteins via GCE (Figure 5j).

### 3.6 Conclusion

In conclusion, the advancements in GCE technology have demonstrated significant progress in the incorporation of ncAAs into proteins. These developments span multiple strategies, including the engineering of aaRS/tRNA pairs, optimizing interactions between tRNA and translation components, and employing innovative laboratory evolution techniques such as PACE, PANCE, and VADER. Additionally, the biosynthesis of ncAAs has emerged as a promising alternative to chemical synthesis, addressing the inherent technical complexities and high production costs associated with the latter. Future research will likely continue to refine these techniques, overcoming existing limitations and further enhancing the efficiency and specificity of ncAA incorporation.

## 4 The Emerging Applications of GCE Technology

### 4.1 Synthetic Biology

#### 4.1.1 Regulation of Gene Expression

##### 4.1.1.1 Direct Regulation

**Figure 6:**
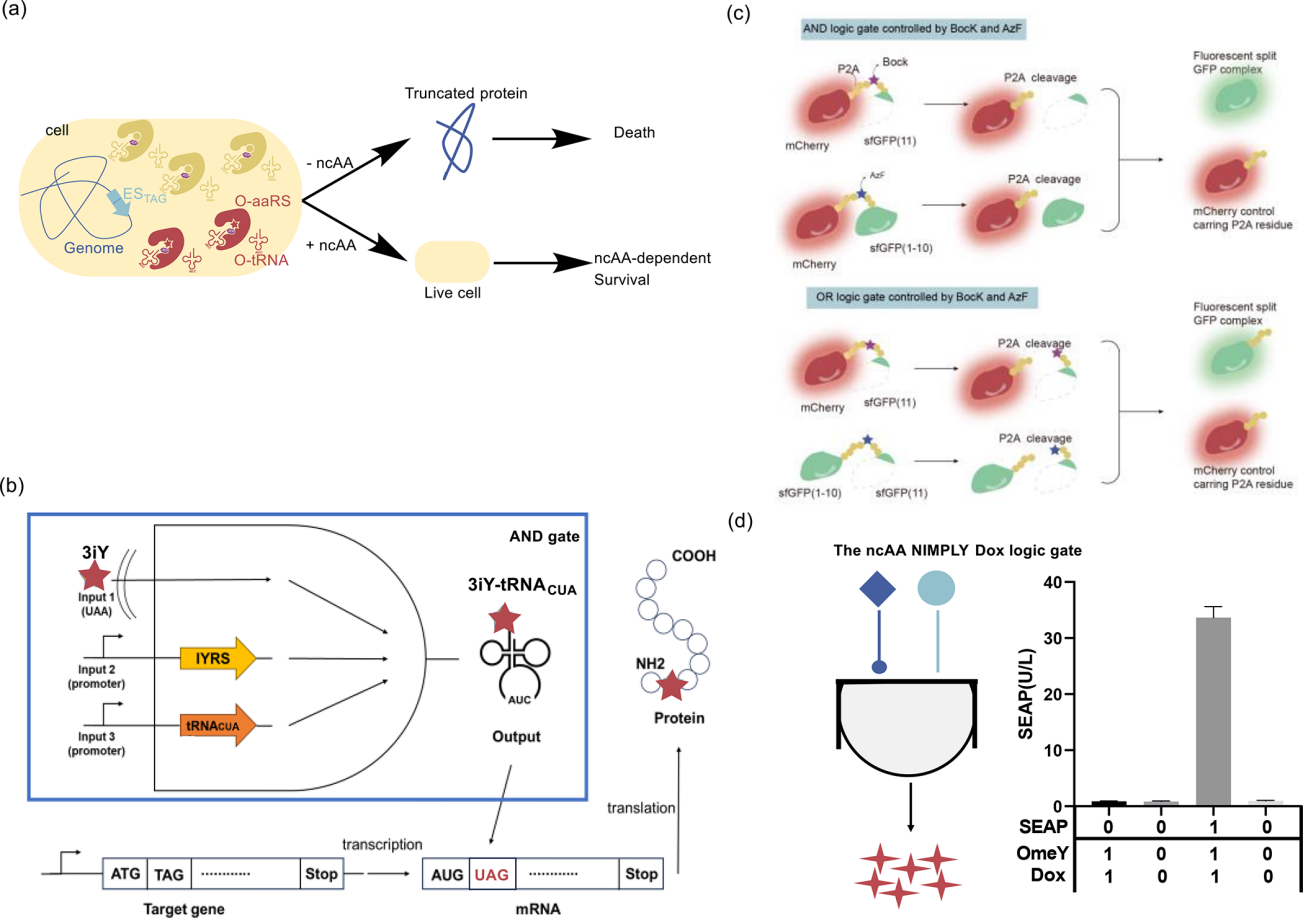
Regulation of gene expression. (a) Direct regulation, ncAA-dependent synthetic auxotroph. (b) AND gate that integrates 3 inputs (ncAA plus 2 promoters that respectively drive RS gene and tRNA) and controls ncAA-tRNA formation as an output. (c) AND/OR logic gate controlled by BocK and AzF. The transfection efficiency of vector expressing split GFP and mCherry into mammalian cell was demonstrated by mCherry fluorescence, which maintained consistent intensity across all four conditions: no ncAA, BocK only, AzF only, or both BocK and AzF. The logic operation output was indicated by GFP fluorescence, mCherry fluorescence indicated transfection efficiency. (d) The ncAA NIMPLY Dox logic gate. Designer cells engineered with the transcription-translation combination switch were subjected to various treatments with OmeY and/or Dox, a secreted embryonic alkaline phosphatase (SEAP) was selected as the POI for convenient detection.

Precise manipulation of protein function has opened up new avenues for regulating cellular activities, including both expression and activity control. While traditional approaches typically manipulate promoter elements to regulate protein expression at the transcriptional level,^191^ GCE technology can offer both transcriptional and translational way to precisely control protein expression.^192^ Direct regulation involves a single molecule directly affecting the function or expression of another molecule. It is characterized by simplicity and specificity, with a relatively fast response speed. By introducing “blank codons” into target genes and using GCE tools, one can directly regulate the expression of full-length protein expression through the addition or removal of ncAAs. This approach paves the way not only for exploring and controlling protein functions but also for pioneering therapeutic interventions. For example, a tight regulatory system for gene expression is typically manipulated by non-natural and bioorthogonal agents, thus ncAA-mediated direct regulation can be applied for the effective development of synthetic gene circuits. For example, Kato^193^ introduced a universal strategy for creating precise protein translation switches by integrating site-specific ncAA systems with positive feedback derepression circuits, significantly reducing unintended translation in the absence of 3iY. Utilizing this feedback mechanism in *E. coli*, they achieved an impressive ON/OFF ratio of 1400, which is 300 times higher than its predecessor system and comparable to the efficiency of the renowned *araBAD* promoter and *araC* regulator-based expression system. On the other hand, by inserting amber stop codons into genes crucial for cell growth, particularly those involved in central carbon metabolic pathways, direct regulation of these essential genes can be effectively achieved through the presence or absence of ncAAs. Volkwein et al.^194^ developed a versatile toolbox utilizing amber suppression with *N*^ε^-acetyl-l-lysine (AcK) for precise protein level adjustment by employing the sensitive LuxCDABE luminescence system and inserting a UAG stop codon into the *luxC* gene. Through altering the sequence near the amber codon to identified motifs that eliminate background signal, insertion of two genetic codons of proline before the amber codon was identified to significantly reduce background luminescence, and supplying AcK into the growth medium led to a luminescence boost of more than 6-fold. A modified *E. coli* strain with the Pro-Pro amber motif in *lacZ* gene were then created and only grows on lactose when AcK was concomitantly supplied, verifying the AcK-dependent translational regulation of essential genes with a diproline amber context. Additionally, Tian et al.^195^ developed a new approach called GCE-based cell growth and biosynthesis balance engineering (GCE-CGBBE). This method involves the precise regulation of gene expression in a way that balances the cell growth and the metabolic flux, which impacts the production of certain compounds or metabolites. Specifically, In *E. coli* Δ321AM,^106,116^ the *p*-acetyl-l-phenylalanine (pAcF) concentration acted as a regulator of glycolytic flux, controlling the expression of phosphofructokinase through the insertion of an amber stop codon into the corresponding *pfkA* gene, whose expression could be thus regulated by titrating pAcF in the medium, thus GCE-CGBBE was used to balance glycolysis and *N*-acetylglucosamine production. Similarly, GCE-CGBBE was also extended to *Bacillus subtilis* to balance the biosynthesis of *N*-acetylneuraminic acid and *O*-methyl-l-tyrosine (OMeY)-dependent growth by controlling the expression of essential genes. Moreover, the regulation of OMeY-dependent essential gene expression ensures biocontainment of these three engineered *B. subtilis* strains to prevent unintended proliferation in natural environments. Thus, direct regulation by the availability of ncAAs is also applied for ncAA-dependent synthetic auxotrophs (Figure 6a). Also, Zhao et al.^81^ presented engineered chimeric phenylalanine systems that enable ncAA incorporation into proteins both at single and multiple sites, with comparable efficiency to canonical amino acids (cAAs) and high fidelity. Based on the refined chimeric Phe systems, they generated a range of *E. coli* synthetic auxotrophs that rely solely on externally provided 4-azido-phenylalanine (AzF) for growth by introducing in-frame amber codons into the essential genes (*dnaN*, *adk*, *tyrS*, and *pgsA*), and these synthetic auxotrophic cells were further demonstrated to exhibit robust AzF-dependent growth in living mice. Moreover, the development of ncAA-dependent synthetic auxotrophs has emerged as a powerful strategy for facilitating the creation of safe, live-attenuated vaccines besides ensuring the biocontainment of GROs.^196,197^ The Guo group^198^ pioneered an amber suppression-based genetic switch to manipulate synthesis of HIV-1 essential protein. By comparing of efficiency and fidelity of three aaRS-tRNA pairs and corresponding ncAAs (pAzF, pAcF and 4-iodophenylalanine, IodoF) at different incorporation sites, they successfully achieved precise control of HIV-1 replication by supplying or withdrawing AzF as an on/off switch, which enhances vaccine safety and allows for temporal control of HIV-1 viability, mimicking prime-boost vaccination strategies.^199^ To address the risk of viruses mutating to escape biocontainment, multiple amber codons can be introduced into essential viral genes or regions with lower mutation rates. Alternatively, the Guo group^200^ further developed a strategy using a quadruplet codon, which prevents virus replication through nucleotide substitution, as it causes a + 1 frameshift. This approach involves constructing a HIV-1 mutant that can be activated by decoding a specific quadruplet codon with a ncAA, offering potential for both research and vaccine development.

GCE also offers a promising solution for genome-relative applications requiring stringently directed regulation, such as genome editing and maintenance of synthetic genome stability. For example, Suzuki et al.^201^ developed a heritable, tightly controlled Cas9-mediated mammalian genome editing system, responding to the nonphysiological lysine derivative *t*-butyloxycarbonyl lysine (BocK) incorporation such that Cas9 (Cas9Boc) was expressed in a full-length, active form in the presence of BocK. Recently, due to the lack of a straightforward and tightly controlled regulation of the synthetic chromosome rearrangement and modification by *loxP*-mediated evolution (SCRaMbLE) process, Zhang et al.^202^ introduced a novel approach for precise regulation of SCRaMbLE recombination in a ncAA dose-dependent manner via GCE, named as GCE-SCRaMbLE, which accurately controls Cre recombinase activity by the site-specific incorporation of OMeY into Cre, the resulting active Cre recombinase variants (Cre_UAG5_ and Cre_UAG14_) offered GCE-SCRaMbLE two distinct advantages: minimal background activity and flexible adjustability of *loxP*-mediated recombination via OmeY supplement. Deep sequencing analysis confirmed no SCRaMbLE events on synthetic chromosomes without OMeY, demonstrating that SCRaMbLE recombination frequency can be precisely modulated by altering Cre expression through growth conditions (with or without OMeY) at the translational level, thus demonstrating Cre recombinase abundance play a key role on the rearrangement outcomes of SCRaMbLE. In brief, directed regulation combined with GCE have demonstrated impressive control over gene expression and metabolic pathways, with applications extending to biocontainment, vaccine development, and synthetic auxotroph creation. GCE also enables tightly controlled genome editing and synthetic genome stability maintenance, exemplified by advancements in Cas9-mediated editing and SCRaMbLE recombination regulation.

##### 4.1.1.2 Logic Gate Regulation

Genetic circuits, composed of various biological components, play important roles in precisely controlling and reprogramming cellular functions. These circuits include genetic switches, inverters, oscillators, and logic gates, etc.,^203−205^ enabling the control of gene expression in a manner akin to electronic logic gates (AND, OR, NOT, etc.). In contrast to directed regulation, logic gate regulation combines multiple input signals to achieve complex signal integration and output. It offers higher flexibility but is more complex, and the response speed may be slower. These gates can response to specific stimuli (such as the presence of certain chemicals) to trigger specific outcomes (e.g., gene expression).^206^ The integration of GCE and ncAAs into these circuits introduces a higher level of specificity and versatility, enabling the development of more sophisticated and finely tuned regulatory networks. This approach allows for the precise modulation of biological processes, enhancing the potential for innovative synthetic biology applications. The use of GCE and ncAAs in constructing logic gates underscores the importance of these technologies in creating intricate cellular control mechanisms that can respond dynamically to a wide range of environmental cues, thereby broadening the scope of programmable gene expression and cellular behavior.

In genetic circuits, the ideal genetic components should be orthogonal to each other and operate independently of existing cellular processes. With an emphasis on achieving orthogonality and independence in these genetic parts, Minaba et al.^203^ engineered innovative AND gate and translational switches based on the incorporation of the ncAA 3iY via GCE in *E. coli* system (Figure 6b). This AND gate that integrates 3 inputs (ncAA plus 2 promoters that respectively drive RS gene and tRNA) and controls ncAA-tRNA formation as an output. Hence the lack of either an input (3iY, RS, or tRNA) turned off the translation of target genes. They applied this gate integrating both transcriptional and translational switches (including ncAA 3iY, inducible promoter *P*_*tac*_ or constitutive promoter *P*_*tyrRS*_ drives RS, inducible promoter *P*_*T7*_ or constitutive promoter *P*_*Ipp*_ drives tRNA, and gene encoding EGFP or ColE3e with zero to three amber codons inserted next to the start codon which is driven by *P*_*T7*_ promoter) to construct a gene expression platform capable of producing significant protein quantities upon induction conditions, while maintaining zero-leakage expression during repression conditions. Building on this concept, Liu et al.^192^ introduced another scalable design for small-molecule control of gene transcription in *E. coli*, leveraging ncAAs and *cis*-regulatory leader-peptide elements including *trp* operon and *tna* operon. This approach enables the use of ncAAs as molecular switches to either attenuate or activate gene transcription. By incorporating blank codons (codon_BL_s) into leader-peptide sequences, which are specifically decoded by tRNAs charged with ncAAs, the system can toggle transcriptional states ON or OFF, allowing for precise gene transcription control and implementation of logic gates within gene regulatory networks. To demonstrate this feature, they constructed NOR (based on the *trp* operon) and OR (based on the *tna* operon) gates using two distinct ncAA inputs (pAzF and pAcF) in the presence of excess tryptophan, facilitated by the specific recognition of a shared tRNA_CUA_ by AzFRS^207^ and AcFRS^208^ enzymes. Specifically, NOR gate: When neither AzF nor AcF are present, the ribosome stalls at codon_BL_, forming the 2:3 structure, allowing transcription to continue. If either AcF, AzF, or both are present, the ribosome does not stall at codon_BL_, leading to the formation of the terminator structure 3:4, halting transcription, OR gate:without AcF and AzF, the ribosome stalls at codon_BL_ and does not reach the Rho termination factor binding site (*rut*), causing transcription to terminate. When either AcF, AzF, or both are present, the ribosome does not stall at codon_BL_ but stalls over the *rut* site, allowing transcription to continue.

Besides *E. coli* cell logic gates, mammalian cell logic gates offer immense potential for diverse applications. However, most of the currently available gates are regulated by drug-like molecules that possess inherent biological activities. To establish truly independent circuits and synthetic regulatory pathways, Mills et al.^209^ harnessed GCE and engineered logic gates that are controlled by two biologically inert ncAAs (BocK and AzF) (Figure 6c). They quantified quadruplet-decoding efficiency of 11 Pyl tRNA variants previously tested in *E. coli* capable of decoding CUAG or AGGA in HEK293 cells. Leveraging a quadruplet-decoding orthogonal pair PylRS/tRNA_UCCU(Ev2)_ in combination with an amber-decoding pair TyrRS*/tRNA_CUA_, they successfully engineered AND and OR logic gates controlled by BocK and AzF, thereby expanding the potential of GCE and the capabilities of mammalian cell logic circuits. Recently, Chen et al.^210^ developed a therapeutic system based on a logic gate enabled by GCE, known as the ncAAs-triggered therapeutic switch (NATS) in mammalian cells and diabetic model mice. This innovative approach allows for rapid therapeutic protein expression when exposed to *O*-methyl-tyrosine (OmeY). The NATS system allows for fast and controllable protein expression at the translational level. The system’s adaptability was further evidenced by successfully implementing a transcription-translation combination switch with availability of different OmeY dose and/or Dox treatments. By incorporating an ectopic amber codon into a Dox-regulated transcriptional factor tTA, this approach was designed to facilitate stringent control over the expression of the protein of interest (POI) using a NIMPLY logic gate pattern. Indeed, the combined NIMPLY system exhibited virtually no background expression (Figure 6d).

#### 4.1.2 Protein Engineering

Protein engineering has traditionally centered on three key strategies to improve enzyme stability and catalytic efficiency: rational design, directed evolution, and semirational design.^211,212^ Although these methods are effective, their dependence on the 20 cAAs limits their application scope. In contrast, the incorporation of ncAAs through GCE has marked a significant advancement, offering a novel approach to introduce unique functional groups into proteins. With access to hundreds of structurally diverse ncAAs, this innovation significantly broadens the possibilities in enzyme design and engineering. The use of ncAAs has not only enhanced protein properties and integrated new catalytic mechanisms into proteins, but also made switchable regulation of protein activity, overcoming the limitations of the natural genetic code.

##### 4.1.2.1 Enhancing Catalytic Properties

**Figure 7:**
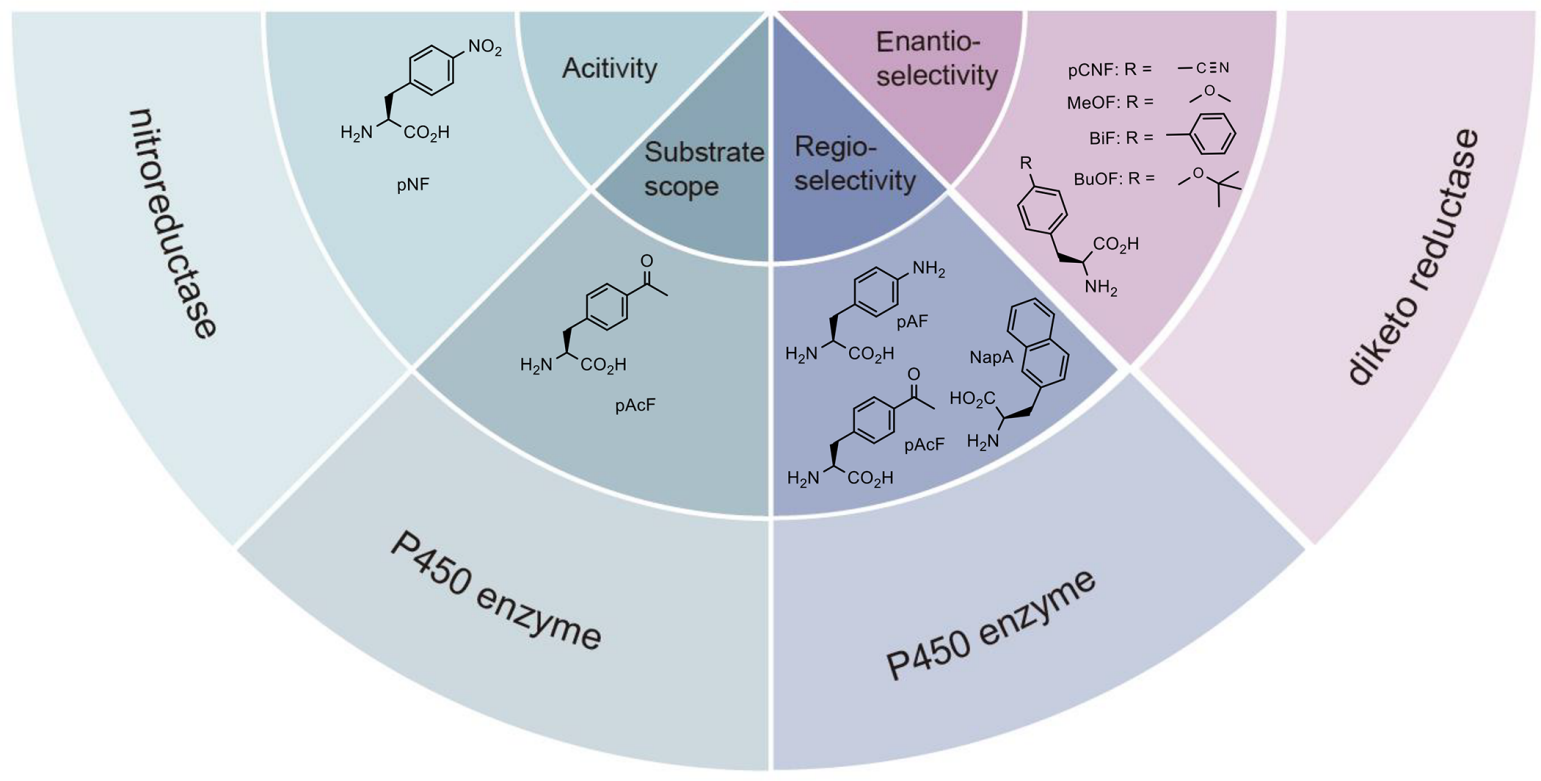
Enhancing native catalytic properties of natural enzymes, including enzyme activity, substrate scope, regioselectivity, and enantioselectivity.

The critical factor behind the significant speed-up of reactions by enzymes lies in how well the active center’s structure matches the transition state of the reaction it catalyzes. Therefore, directed evolution studies have demonstrated that alterations in the overall protein structure, even distant from active center, can impact catalysis by inducing changes in the structure of active center.^213^ NcAAs offer a means to precisely adjust spatial and electronic interactions within active sites in manners that cAAs would struggle to achieve (Figure 7). The first example reported by Jackson et al.^214^ is about attempt to improve catalytic activity of prodrug activator nitroreductase from *E. coli* by substituting the active site Phe124 site with different cAAs and ncAAs, they finally found a greater than 30-fold increase in catalytic efficiency (*k*_cat_/*K*_M_) using *p*-nitrophenylalanine (pNF) compared to the native active site, and a more than 2.3-fold enhancement over the most effective cAA lysine. Additionally, by implementing a semirationally designed substrate binding strategy to change the substrate specificity of a biosynthetic P450 enzyme (PikC), Pan et al.^215^ incorporated various ncAAs at position His238 to reshape the desosamine sugar binding pocket of PikC, which identifies its natural macrolide substrates with a 12- or 14-membered ring macrolactone attached to a deoxyamino sugar.^216,217^ The incorporation of ncAAs imparts unnatural activities to several PikC variants, enabling them to react with the unglycosylated macrolactone precursors 10-deoxymethonolide and narbonolide, an outcome unachievable with the original enzyme, and PikC_H238pAcF_ which features a pAcF residue at position His238 gives the highest hydroxylation activity. They further engineered PikC_H238pAcF_ to improve binding affinities for the two substrate precursors and obtained the resulting PikC_H238pAcF/E85Q_ with increased binding affinities. The pikromycin biosynthetic pathway with the natural glycosylation-oxidation order was sequentially reversed by PikC_H238pAcF/E85Q_ for first hydroxylation reaction and the versatile glycosyltransferase BSGT-1^218^ for subsequently glucosylating hydroxylated macrolactones. On the other hand, altering the regioselectivity and enantioselectivity of enzymes through engineering represents a promising biotechnological application.^219,220^ Incorporating ncAAs known to alter activity also results in concurrent shifts in regioselectivity and enantioselectivity. For example, Kolev et al.^221^ explored the effect of the ncAA incorporation at 11 positions within the active site of a bacterial P450 (CYP102A1) on the regioselectivity for the hydroxylation of (*S*)-ibuprofen methyl ester and (+)-nootkatone, and discovered that substituting Leu75 in with *p*-aminophenylalanine (pAF) significantly enhanced regioselectivity toward the oxidation at position C2′ of (*S*)-ibuprofen methyl ester. Additionally, the introduction of pAcF at Ala82 position resulted in much higher regioselectivity (62%) for oxidation at position C13′ of (+)-nootkatone than that of the wide-type enzyme (4%). Similarly, variants featuring pAcF substituting Ala78 and 3-(2-naphthyl)-alanine (NapA), replacing Ala328 showed significantly enhanced regioselective hydroxylation at the C1′ (88%) and C2′ (95%) positions of (*S*)-ibuprofen methyl ester, markedly better than the WT’s 62% and 38% hydroxylation at these sites, respectively. Trp222 in diketo reductase (DKR), an enzyme that converts various ketones into chiral alcohols with varying enantiotope selectivity, is situated at the hydrophobic interface between dimers at the C-terminus, yet it does not directly engage in the interaction between the enzyme and its substrates.^222−224^ Ma et al.^225^ demonstrated Trp222 serves as a gatekeeper role, controlling the substrate’s entry to the DKR active site. To further explore how the size of residue 222 affects the enantio-preference, they introduced four ncAAs (*para*-cyanophenylalanine, pCNF; *para*-methoxyphenylalanine, MeOF; *para*-phenylphenylalanine, BiF; *O*-*tert*-butyltyrosine, BuOF) with varying molecular volume of side chains at this key site using site-specific incorporation via GCE. Then these resulting proteins harboring different ncAAs reduced the ketone substrate 2-chloro-1-phenylethanone, resulting in various enantiomeric combinations of the alcohol product. The wild-type DKR produced a relatively low enantiomeric excess (ee) value, while the variants (DKR_W222pMeOF_, DKR_W222BiF_, and DKR_W222pButOF_) with larger size of ncAA residues showed significantly higher ee values, maintaining the *R* enantiomer. For DKR_W222pCNF_, which has the smallest ncAA pCNF at position 222, there was a switch in selectivity favoring *S* enantiomer.

##### 4.1.2.2 NcAAs Serve as Unnatural Catalytic Residues

###### 4.1.2.2.1 Genetic Encoding of Nucleophilic Catalysis

**Figure 8:**
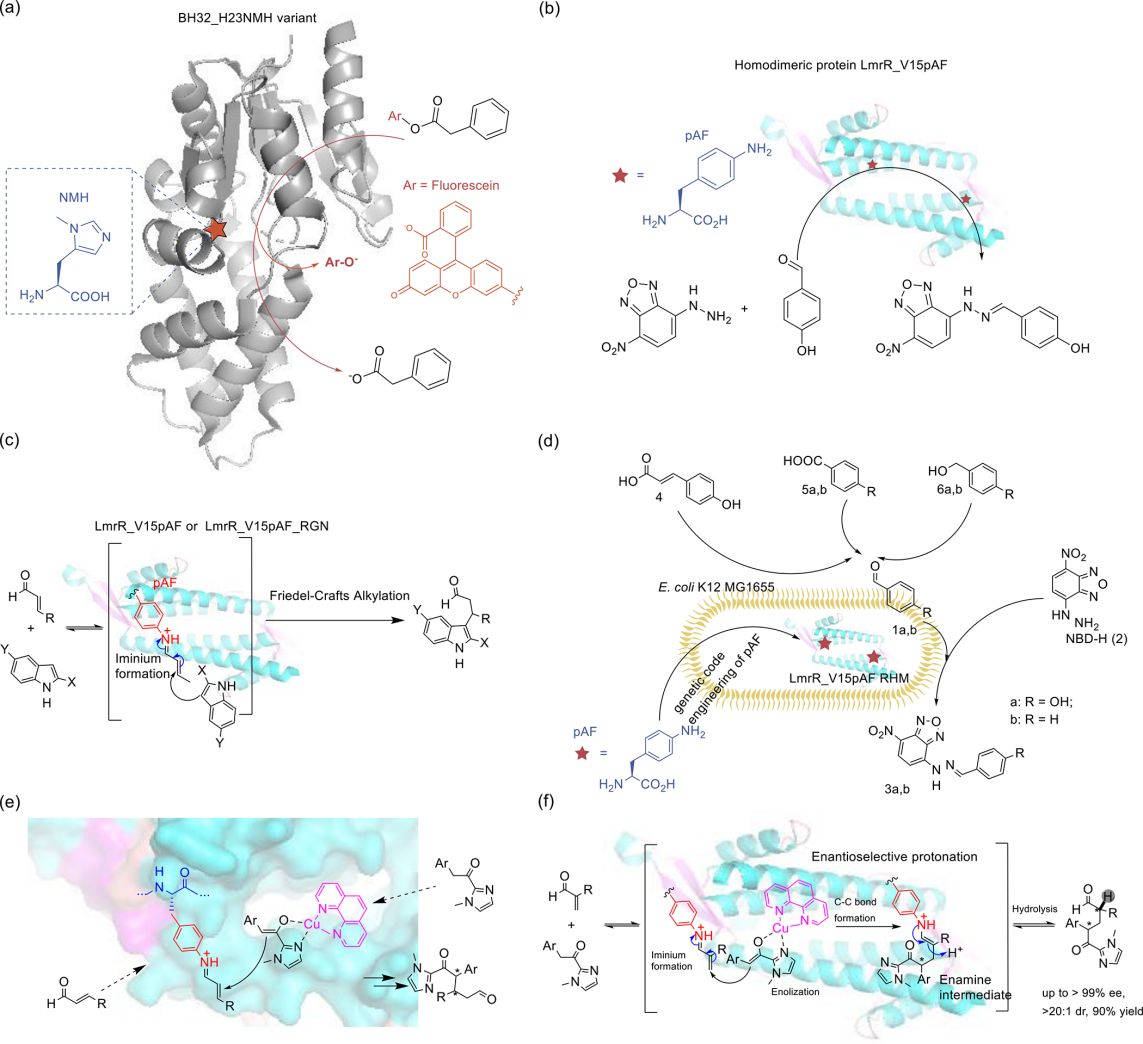
Genetic encoding of nucleophilic catalysis. (a) Reaction scheme for the hydrolysis of fluorescein 2-phenylacetate catalyzed by BH32 (His23Me-His), the reaction’s progress was tracked by observing a rise in absorbance at 500 nm, which indicated the formation of fluorescein. (b–f) LmrR-based designer enzymes. LmrR containing pAF catalyzes chromogenic hydrazone formation between 4-hydrazino-7-nitro-2,1,3-benzoxadiazole and 4-hydroxybenzaldehyde, the reaction’s progress was tracked by the following product in absorbance at 472 nm (b). Unlocking iminium catalysis in artificial LmrR variants to create a Friedel–Crafts alkylase (c). In vivo biocatalytic cascades featuring an unnatural hydrazone formation reaction catalyzed by LmrR_V15pAF_RMH (d). An asymmetric Michael addition reaction catalyzed by synergistic combination of two catalytic sites, the pAF residue functions for activation of the enal and Cu(II)-phen for the generation and delivery of the enolate nucleophile (e). A tandem Michael addition/enantioselective protonation reaction catalyzed by LmrR_V15pAF employing two abiological catalytic sites in a synergistic fashion: a genetically encoded pAF and a Lewis acid Cu(II) complex (f).

Developing designer enzymes capable of catalyzing non-natural chemical transformations presents a formidable challenge. Traditionally, efforts in this direction have primarily focused on utilizing cAAs during the initial design phase. Natural enzymes often achieve remarkable acceleration rates by precisely positioning side chains within a protein binding pocket, thereby enhancing their inherent catalytic abilities. This level of fine-tuning is also applicable to catalytic ncAAs, the incorporation of ncAAs featuring distinct and reactive side chains holds the potential to greatly broaden the catalytic capabilities of designer enzymes. For example, *N*_*δ*_-methylhistidine (NMH) can be regarded as a genetically encodable substitute for the commonly used nucleophilic catalyst dimethylaminopyridine.^226^ Burke et al.^227^ designed a novel hydrolytic enzyme based on a computationally designed hydrolase BH32 by employing NMH as a noncanonical catalytic nucleophile to replace His23 nucleophile via GCE. Followed by directed evolution, the resulting enzyme (BH32_H23NMH_L10P_A19H_S22M_E46N_P63G_C125G) obtained capability of accelerating fluoresceinyl ester hydrolysis with an extraordinary efficiency of over 9000-fold improvement compared to free NMH in solution (Figure 8a). On the other hand, anilines are widely recognized as nucleophilic catalysts for the formation of hydrazones (X = NH) and oximes (X = O).^228,229^ Drienovská et al.^230^ chose *p*-aminophenylalanine (pAF) harboring an aniline side chain as a potentially innovative catalytic nucleophile. By incorporating pAF residue at position V15 into the hydrophobic pore at dimer interface of the multidrug resistance regulator from *Lactococcus lactis* (LmrR),^231^ the catalytic activity of the aniline side chain in hydrazone and oxime formation reactions was enhanced, outperforming wild-type LmrR, aniline and more reactive aniline derivatives. The success of the artificial enzyme LmrR_V15pAF stems from both the promiscuous ability of binding pocket to bind substrates and the strategic placement of pAF residue containing the aniline side chain, which acts as a catalytic residue. Mayer et al.^232^ further engineered the designer enzyme LmrR_V15pAF through successive rounds of directed evolution for employing an aniline side chain to catalyze the model hydrazone formation reaction, and multiple mutations were obtained within the versatile binding pocket where the ncAA resides in the original catalyst. When these mutations were combined, they dramatically increased the turnover frequency (*k*_cat_) of the evolved designer enzyme (LmrR_V15pAF_RMH) by nearly 100-fold, demonstrating improving the intrinsic catalytic performance of an artificial side chain can be achieved through the discovery of advantageous mutations within a protein framework (Figure 8b). Additionally, the original enzyme LmrR_pAF was used by Gower et al.^233^ for other purpose to design the vinylogous Friedel–Crafts alkylation reaction between α,β-unsaturated aldehydes and indoles. pAF also serves as a catalytic residue, activating enal substrates by forming intermediate iminium ion species and facilitating conjugate addition. Through directed evolution, an improved mutant LmrR_V15pAF_RGN was identified and displayed enhanced yields and enantioselectivities across a range of aliphatic enals and substituted indoles (Figure 8c). Importantly, in contrast to previously reported Cu(II)-based artificial enzymes used for Friedel–Crafts alkylation reactions,^234^ the reaction catalyzed by LmrR_V15pAF_RGN is metal-free and eliminates the requirement of a chelating group for substrates. Further comparative analysis of the Michaelis–Menten kinetics of LmrR_V15pAF, LmrR_V15pAF_RGN, as well as the previous mutant LmrR_V15pAF_RMH^232^ revealed the LmrR_pAF evolves to acquire new activities at the cost of existing ones, indicative of divergent functional evolution that specializes catalytic reactivity. Therefore, the potential of designer enzymes incorporating pAF as catalytic residues acts as promising starting points for directed evolution endeavors. Additionally, as LmrR_V15pAF is a genetically encoded artificial enzyme, it can be synthesized within living cells, leading to conjecture that the hydrazone formation reaction it catalyzes might also occur in vivo. Atta et al.^235^ presented biocatalytic cascades mediated by the variant LmrR_V15pAF_RMH,^232^ which was obtained by direct incorporation of pAF and efficiently transformed benzaldehyde derivatives produced within *E. coli* cells into the corresponding hydrazone products (Figure 8d). These in vivo biocatalytic cascades, characterized by the involvement of artificial enzymes, represent a significant stride toward the creation of hybrid metabolism. On the other hand, Zhou and Roelfes^236^ designed another ingenious artificial enzyme based on the LmrR_V15pAF protein^230^ featuring an indirectly introduced pAF residue via GCE, capable of serving as a nucleophilic catalyst. A Lewis acidic Cu(II) bound to the ligand 1,10-phenanthroline (Cu(II)-phen complex) nestling between the indole rings of two tryptophan residues situated in the hydrophobic pore was then introduced via supramolecular interaction^237^ with LmrR_V15pAF. LmrR_V15pAF variants by site mutagenesis yielded improved catalytic activity and high enantioselectivity (up to >99% ee) compared to wild-type LmrR with no catalytic activity in the asymmetric Michael addition reaction in water, which takes place due to a synergistic combination of iminium ion activation of the α,β-unsaturated aldehyde by the pAF residue and the Cu(II)-phen complex-induced enolization of the ketone (Figure 8e). Additionally, enantioselective protonation represents a highly appealing concept for the creation of α-chiral centers.^238^ Due to the effectiveness of the synergistic catalysis principle^236^ in artificial enzymes, they^239^ further aimed to employ the LmrR_V15pAF-based artificial enzyme for catalyzing the challenging tandem Michael addition/enantioselective protonation reaction in water. The excellent enantioselectivity in proton transfer to an enamine intermediate is evident with diastereomeric ratio (up to >20:1) and enantiomeric excess value (up to >99%) of the resulting product (Figure 8f), underscoring the potential of harnessing synergistic catalysis within artificial enzymes for addressing challenging reactions.

###### 4.1.2.2.2 Genetic Encoding of Photocatalysis

**Figure 9:**
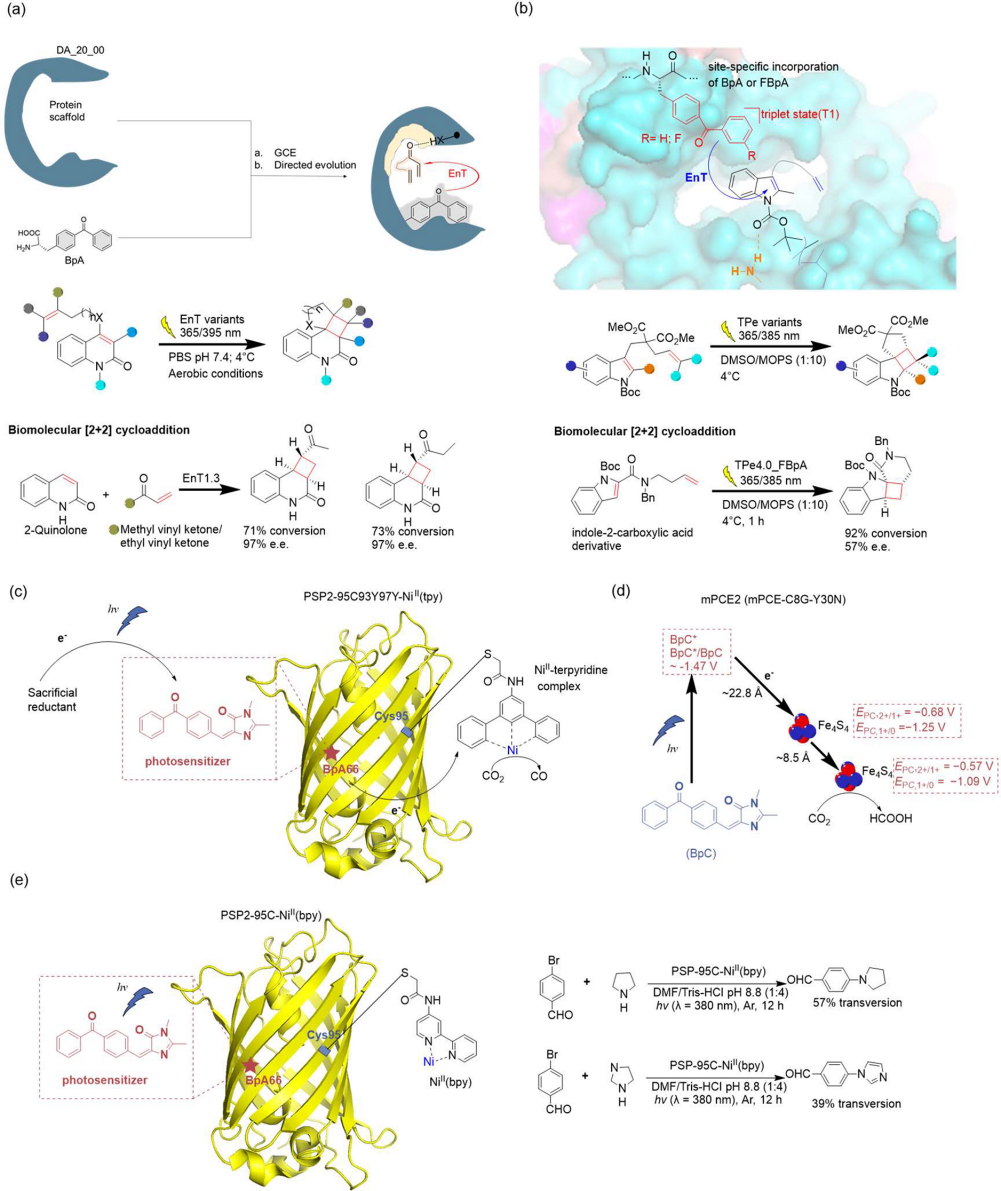
Genetic encoding of photocatalysis. (a,b) Photoenzyme design. A designed photoenzyme based on DA_20_00 for enantioselective [2 + 2] cycloadditions, photoenzyme development, substrate scope of EnT1.3 and selected variants and biomolecular [2 + 2] cycloadditions (a). Enantioselective [2 + 2]-cycloadditions with triplet photoenzymes. The design of TPe, the substrate scope of TPe variant and biomolecular [2 + 2] cycloadditions (b). (c–e) Light-harvesting metalloenzyme design. PSP was transformed into the resultant photocatalytic CO_2_-reducing enzyme PSP2–95C93Y97Y by employed the nickel (Ni^II^)–terpyridine complex, which catalyzes the selective CO_2_ reduction to CO (c). PET pathways in miniature photocatalytic CO_2_-reducing enzyme mPCE2 (mPCE-C8G-Y30N). A strongly reducing ketyl radical (BpC^•^/ BpC; *E*_pc_ = −1.47 V) is generated by charge separation in mPCE2, and sequential ET produces the reduced [Fe_4_S_4_] cluster (Fe_A,red_/Fe_A,ox_; *E*_pc,2+/1+_ = −0.68 V, *E*_pc,1+/0_ = −1.25 V, and Fe_B,red_/Fe_B,ox_; *E*_pc,2+/1+_ = −0.57 V, *E*_pc,1+/0_ = −1.09 V) for CO_2_ reduction (d). Biocatalytic cross-coupling of aryl halides with a genetically engineered photosensitizer artificial dehalogenase, which features a genetically encoded benzophenone chromophore and site-specifically modified synthetic Ni^II^(bpy) cofactor with tunable proximity to streamline the dual catalysis, which was shown on the left pane. Catalytic C–N bond formation by PSP-95C–Ni^II^(bpy) was shown on the right pane (e).

Incorporating key photocatalytic processes into proteins, like triplet energy transfer (EnT), has the potential to develop artificial photoenzymes that broaden the range of traditional biocatalytic activities.^240,241^ For this purpose, Trimble et al.^242^ harnessed the expanded genetic code to engineer a novel photoenzyme based on EnT catalysis, a versatile reactivity mode in organic synthesis currently inaccessible to biocatalysis. By introducing genetically encoded 4-benzoylphenylalanine BpA bearing photosensitizer benzophenone (BP) into at several positions around the active site pocket of DA_20_00,^243^ they found inserting BpA at site 173 in the hydrophobic cavity of DA_20_00 resulted in the creation of the active photoenzyme EnT1.0 for [2 + 2] cycloaddition (Figure 9a). Through further directed evolution, they achieved an efficient and highly selective enzyme (EnT1.3, with up to 99% ee) capable of catalyzing both intramolecular and bimolecular cycloadditions, including reactions that have proven challenging to selectively achieve with small molecule catalysts. Notably, EnT1.3 displayed over 300 turnovers and was effective under aerobic conditions and at ambient temperatures, distinguishing it from small-molecule photocatalysts. These progresses pave the way for an abundance of novel excited-state chemistry within protein active sites and lay the foundation for the creation of a new class of enantioselective photocatalysts. Similarly, using GCE technology combined with directed evolution, Sun et al.^244^ developed another photoenzymes based on LmrR,^231^ which features a large hydrophobic binding pocket at its dimer interface. They site-specifically inserted BpA or 3′-fluoro-BpA (FBpA) as a photosensitizer to construct an artificial photocatalytic center at position F93 within chiral cavity of the LmrR protein, thereby creating engineered triplet photoenzymes (TPes) (Figure 9b), thereby achieving enantioselective intramolecular [2 + 2] photocycloaddition reactions of indole derivatives and exhibiting excellent substrate compatibility and outstanding enantioselectivity (up to 99% ee).

Additionally, photocatalysts, when combined with artificial metalloenzymes, have been employed to transform light energy into a variety of chemical compounds. It is known that superfolder yellow fluorescent protein (sfYFP) produces a highly fluorescent *p*-hydroxybenzylidene-5-imidazolinone species via the self-catalyzed transformation of its tripeptide Gly65-Tyr66-Gly67. The intersystem crossing of benzophenone is notably efficient, achieving nearly 100% quantum efficiency as it transits from the singlet excited state to the triplet state, which persists 10^5^-fold longer lifetime, facilitating sacrificial reduction.^245−248^ It is thus easy to imagine that the tripeptide sequence Gly65-BpA66-Gly67 within the sfYFP containing a BpA66 residue with a benzophenone chromophore is capable of self-catalyzing into a chromophore composed of (*E*)–4-(4-benzoylbenzylidene)-1,2-dimethyl-1*H*-imidazole-5(4*H*)-one (BpAChm). Therefore, by substituting Tyr66 chromophore residue of sfYFP with BpA via GCE combined with mutating of residues at sites 230/148, Liu et al.^249^ designed a genetically encoded photosensitizer protein (PSP), which serves as a valuable tool for the rational design for CO_2_ reduction. They successfully obtained the mutant sfYFP-BpA66-D203E148 (PSP2). When exposed to visible light, PSP2 efficiently enters a long-lived triplet excited state (PSP2*), which promptly engages with reduced nicotinamide adenine dinucleotide to produce a highly potent super-reducing radical (PSP2•). To facilitate an efficient electron transfer between PSP2 and catalysts and improve catalytic efficiency, *N*-(2,6,2-terpyridine-4-yl)iodoacetamide was attached covalently to the optimized mutant PSP2–95C93Y97Y with subsequent formation of a nickel (Ni^II^)–terpyridine complex (Figure 9c), resulting in a photocatalytic CO_2_-reducing enzyme termed as PSP2T2 with an impressive CO_2_/CO conversion quantum efficiency of 2.6%. Additionally, photosystem I (PSI) is one of the most important components in photosynthesis machinery.^250^ To recapitulate the key properties of PSI and design a novel miniature photocatalytic CO_2_-reducing enzyme (mPCE), Kang et al.^251^ fused the PSP2^249^ with a [Fe_4_S_4_] cluster-binding protein by genetically linking the first 36 residues of PSP2 to the N-terminal end of ferredoxin from *Clostridium acidurici* (*Ca*FD), a compact protein of 5.9 kDa containing two [Fe_4_S_4_] clusters (FeA/FeB)—and connecting the C-terminal end of CaFD to residues 40–238 of PSP2, generating a full-length mPCE protein (33 kDa). Inspired by the ability of PSI to undergo multiple-step photoinduced electron hopping from P700* to iron–sulfur [Fe_4_S_4_] clusters, the mPCE harbors FeA/FeB and a chromophore (BpC) derivated from the genetically incorporated BpA. By precisely adjusting the reduction potential via site-mutation of the mPCE, they obtained a more reactive mutant mPCE2 (mPCE-C8G-Y30N) (Figure 9d), which enhanced the electron transfer process from BpC to FeA/FeB, achieving a quantum efficiency of 1.43% for the conversion of CO_2_ to HCOOH. Given that mPCE can be abundantly produced in *E. coli* cells without the need for synthetic cofactors, pursuing this approach could lead to swift advancements in photoenzyme quantum yield and broaden its functionalities via a streamlined directed evolution method. Also, based on the CO_2_-reducing PSP2-C95 mutant,^249^ Fu et al.^252^ further developed another novel light-harvesting metalloenzyme for conducting organometallic cross-coupling reactions under mild conditions. Bipyridine (bpy), recognized as an effective ligand for Ni^II^,^253^ was first attached covalently to the PSP2-Cys95 mutant, resulting in the formation of PSP-95C-bpy, followed by complexing with Ni^II^ to produce the photosensitizer metalloenzyme PSP-95C–Ni^II^(bpy), which consequently integrates a genetically encoded BpA and a site-specifically modified synthetic Ni^II^(bpy) cofactor. These two unnatural catalytic components, which may be challenging to harmonize in a standard solution, are now meticulously orchestrated to maintain precise spatial proximity, thereby enhancing the synergy of dual catalysis. The catalytic platform demonstrated remarkable efficacy in facilitating a variety of transformations, including the conversion of diverse aryl halides into phenols and the creation of valuable C–N bonds (Figure 9e). Notably, the incorporation of a purely synthetic metal complex, namely the Ni^II^ cofactor, sets this approach apart from the predominant photobiocatalytic methods that rely on natural redox enzymes.

###### 4.1.2.2.3 Genetic Encoding of Boronic Acid Catalysis

Recently, Longwitz et al.^254^ showcased the full potential of ncAA-based enzyme design by incorporating pBoF as a catalytic residue in LmrR protein, thus enabling the enzyme to function through a xenobiotic mechanism, which is unattainable with only natural proteogenic components. This boron-containing enzyme design utilizes the affinity of boronic acids for vicinal diols, which are transiently formed after nucleophilic attack on carbonyl groups with a hydroxyl directing group, to catalyze a condensation reaction. Unlike typical biocatalytic strategies such as Lewis/Brønsted acid activation or the use of oxyanion holes, the boronic acid activates the substrate through the formation of covalent cyclic boronate esters. This designer enzyme catalyzes the kinetic resolution of hydroxyketones through oxime formation, demonstrating high enantioselectivity for different substrates. The enzyme design and functionality were enhanced through a directed evolution approach, confirming that GCE can create evolvable, enantioselective enzymes using xenobiotic catalytic moieties such as boronic acids, highlighting the potential of integrating ncAAs into proteins to access new reaction mechanisms and advance biocatalysis.

##### 4.1.2.3 NcAAs Function As Noncanonical Ligands

**Figure 10:**
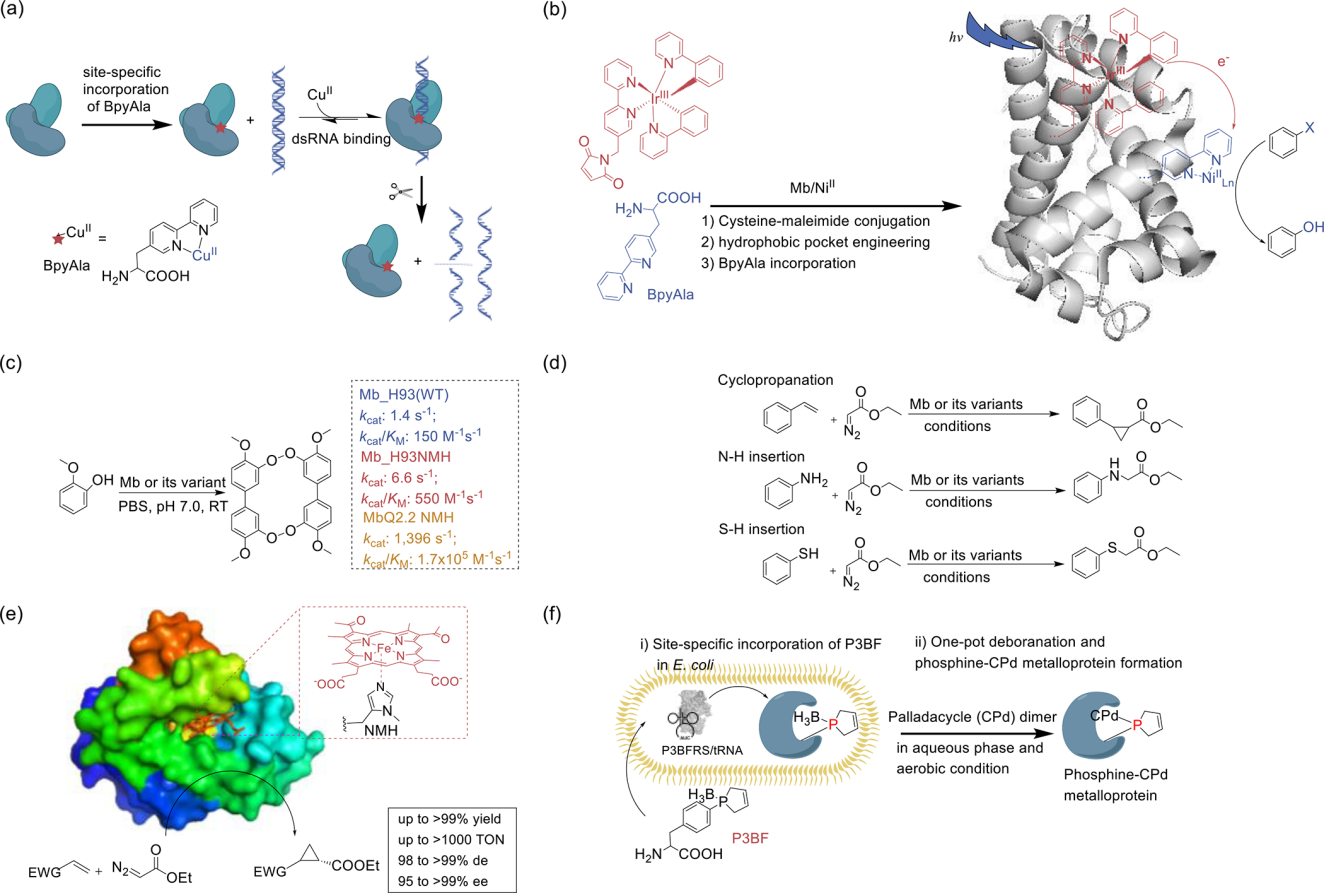
NcAAs function as noncanonical ligands. (a) Site-specific incorporation of BpyAla in two sites of the VSRS protein p19 dimer introduces a metal binding site. (b) Design scheme of an artificial metallaphotoredox enzyme (AMPE*/C45-Z97) by the introduction of the Ir photocatalyst via bioconjugation and metal binding ncAA, BpyAla, via the active site redesign and GCE method. Photocatalytic C–O coupling enzymes that operate via intramolecular electron transfer. (c) Myoglobin (Mb)-based designer enzyme was made by incorporating *N*_δ_-methylhistidine (NMH) with metal-coordination properties into myoglobin (Mb) and the enzyme activity of the resulting enzyme and its derivates was tested in guaiacol oxidation. (d) Reactivity of Mb variants in different carbene transfer reactions. (e) Stereoselective cyclopropanation of electron-deficient olefins with a cofactor redesigned carbene transferase Mb*. (f) Streamlined in vivo platform to achieve the site-specific genetic incorporation of borane-protected phosphine into proteins followed by one-pot deboronation and palladium coordination.

Nature employs a strategy to attain catalytic activities beyond what can be achieved using 20 cAAs by enlisting cofactors like metal ions. The catalytic efficacy of these metal ions heavily relies on their associated ligands. When cAAs fail to deliver the desired catalytic attributes, nature turns to metal complexes, such as heme. Consequently, ncAAs with metal-binding/coordinating capabilities or even act as a catalyst exerting a synergistic catalytic catalysis with metal ions offer a compelling means to bind and influence the characteristics of catalytic metal ions, leading to the creation of artifical metalloenzymes.^10,255^ For example, metal-chelating (2,2′-bipyridin-5-yl)alanine (BpyAla) characterized by its bipyridyl component offers an unconventional side chain that can chelate copper(II) ion, which thus can catalyze the cleavage of the phosphodiester backbone in nucleic acids.^256,257^ To this end, Ahmed et al.^258^ designed a novel unnatural endonuclease based on p19 viral suppressor protein of RNA silencing (VSRS) from Tombusvirus, known for its ability to bind and sequester small RNA duplexes with picomolar affinity. By utilizing GCE, they incorporated BpyAla into the RNA-binding pocket of p19 protein at T111 site to create a catalytic site. Upon binding to copper(II) ion, the p19-T111BpyAla variant exhibits site-specific catalytic cleavage of siRNA and human miRNAs within human cell lines (Figure 10a). They focused on targeting miR-122, a pivotal host factor in hepatitis C virus (HCV) infection, whose level was effectively reduced by the unnatural endonuclease function of p19-T111BpyAla. Additionally, followed by engineering hydrophobic heme pocket of the oxygen-binding protein sperm whale myoglobin (Mb) via triple alanine mutations to remove the heme and enlarge the void space and incorporating the same metal-binding BpyAla for the putative Ni-mediated catalytic cycle via the active site redesign and GCE, Lee et al.^259^ successfully engineered several artificial photocatalytic cross-coupling enzyme variants by incorporating an Ir photocatalyst via cysteine-maleimide conjugation and a bipyridine nickel Ni^II^(bpy) in close proximity, the resulting enzyme harnessed visible light to execute intramolecular electron transfer between the two catalysts, leading to C–O coupling products with exceptional yields of up to 96% (Figure 10b). They further fine-tuned the catalytic activity of these artificial photocatalytic cross-coupling enzymes by adjusting the electrochemical properties of the catalytic elements, their positions, and the distances between them within the protein, resulting in the identification of the most efficient mutant (AMPE*/C45-Z97) that displayed a wide substrate scope under optimized conditions.

Additionally, Pott et al.^260^ replaced the proximal histidine ligand (His93) with a less electron-donating NMH as the proximal heme ligand in Mb, yielding significant enhancements in heme redox potential and versatile peroxidase activity and leading to an approximate 5-fold increase in *k*_cat_ for guaiacol oxidation. This enzymatically altered Mb variant (Mb_H93NMH) displayed notable modifications in the proximal pocket, including changes in hydrogen-bonding interactions with the essential porphyrin cofactor. This Mb_H93NMH was further refined to yield a highly efficient peroxidase biocatalyst (MbQ2.2 NMH) within a globin fold guided by rational design and directed evolution (Figure 10c). Moreover, the same group^261^ further harnessed amber stop codon suppression in conjunction with specially engineered pyrrolysyl-tRNA synthetase variants to substitute the proximal histidine of Mb with ncAAs NMH, 5-thiazoylalanine (5ThzA), 4-thiazoylalanine (4ThzA), or 3-(3-thienyl)alanine (3ThiA). By introducing these noncanonical ligands, they achieved a remarkable tuning of the heme redox potential across a range exceeding 200 millivolts. Importantly, these ligands exerted a profound influence on the protein ability to facilitate carbene transfer reactions using ethyl diazoacetate. Variants with heightened reduction potentials demonstrated superior performance in cyclopropanation and N–H insertion reactions, while those with reduced *E*° values exhibited enhanced S–H insertion activity (Figure 10d). Considering the pivotal role of histidine in numerous enzymes, these genetically encoded analogues offer valuable tools for investigating mechanisms and enabling innovative chemical processes. Substituting NMH ligand in Mb variants has also resulted in the creation of artificial carbene transferases boasting enhanced catalytic characteristics due to the protein’s promiscuous carbene-transfer chemistry. For example, Hayashi et al.^262^ reported that substituting ligands in a modified variant Mb*, containing two mutations in the distal pocket (H64V and V68A), led to modest improvements in the conversion rates during cyclopropanation reactions between styrene and ethyl diazoacetate when a reductant was absent in established anaerobic conditions. Remarkably, changing the ligands in Mb* eliminated the need for an external dithionite reductant, enabling efficient catalysis under aerobic conditions. These enhanced properties were attributed to the increased electrophilicity of the Fe(III) center when it was coordinated by the NMH ligand. In an effort to expand the catalytic capabilities of Mb-based carbene transferases, Carminati and Fasan^263^ combined an unnatural ligand NMH with a non-native electron-deficient heme analogue iron-2,4-diacetyl deuteroporphyrin IX (Fe(DADP)) in Mb* (Figure 10e). The resulting artificial carbene transferase proved highly effective in promoting cyclopropanation of electron-deficient alkene substrates, a task that had proven challenging with previously engineered carbene transferases. This broader range of reactions was attributed to a radical-type carbene transfer mechanism resulting from the combined influence of the electron-deficient heme and the unnatural axial ligand. Therefore, altering the central metal coordination environment stands as a potent method for adjusting the properties of metalloproteins. Similarly, Green et al.^264^ showcased the successful incorporation of a genetically encoded NMH for stoichiometric replacement of the proximal histidine residue (His163) into an engineered heme enzyme ascorbate peroxidase (APX2), which employs a histidine residue as the axial ligand for heme iron, establishing a hydrogen bond between the noncoordinating *N*_δ_ atom and an aspartate residue. This catalytical modification resulted in an altered enzyme with increased robustness toward irreversible deactivation that displayed improved enzymatic turnover (*k*_cat_) without compromising its catalytic efficiency (*k*_cat_/*K*_m_). Notably, this achievement was attained despite the disruption of the previously considered “essential” hydrogen-bonding interaction between His and Asp residues. Additionally, phosphine ligands are the most crucial type of ligands for cross-coupling reactions because of their distinctive electronic and steric characteristics. To genetically incorporate phosphine ligand into proteins, Duan et al.^265^ devised an effective approach for the precise incorporation of (*S*)-2-amino-3-(4-(2,5-dihydro-1*H*-phosphol-1-borane-1-yl)phenyl)propanoic acid (P3BF), a phenylalanine (F) derivative with borane-protected phosphine, into proteins within *E. coli*. P3BF displays outstanding compatibility with biological systems and maintains its stability within the physiological milieu determined by sfGFP-17P3BF featuring an unreported P–B bond in protein structures. The LmrR-15P3BF protein, featuring P3BF at position 15 within the hydrophobic pocket of the homodimer LmrR,^231^ was subsequently created and underwent full transformation into the stable LmrR-15P3F-CPd complex achieved by efficient one-pot deboronation and palladium coordination in aqueous and aerobic conditions (Figure 10f). This innovative and efficient platform equips artificial metalloenzymes with the remarkable chemical property of phosphine ligands.

##### 4.1.2.4 Optical Control of Protein Function

Photo controllable proteins are usually created by substituting an active residue with ncAAs carrying photoprotective groups, optical control of protein function can be achieved by light irradiation. Therefore, GCE technology can be used to develop optical control methods for protein function with temporal and spatial resolution for synthetic biology applications. Specifically, protective groups on the side chains of ncAAs play a blocking role. After irradiation with light of a fixed wavelength, the protective groups on the side chains of ncAAs are removed and the functional groups are released, thereby restoring protein activity.^266^ For example, nitrobenzyl-based photolabile protecting groups (PPGs) have been applied in residues that are usually crucial for protein functions, including *o*-nitrobenzyl glutamic acid (ONBE), *o*-nitrobenzyl tyrosine (ONBY), *o*-nitrobenzyl aspartic acid (ONBD), methyl *o*-nitrobenzyl aspartic acid (MeONBD), and 4,5-dimethoxy-2-nitrobenzyl-l-cysteine (DONBC). Photoactivatable fluorescent proteins are coveted tools for the optical marking and monitoring of specific proteins within live organisms. Yang et al.^267^ endeavored to develop a photoactivatable GFP variant by substituting E222 with ONBE, observing a gradual increase in green fluorescence upon UV light exposure in both *E. coli* and mammalian cells. Additionally, Lee et al.^268^ introduced an innovative approach for in situ production of artificial melanin within native skin by a newly designed photoactivatable tyrosinase PaTy derived from *Streptomyces avermitilis* tyrosinase (SaTy). The Y41 residue was replaced with a photocleavable ONBY through GCE. The ONBY incorporation effectively inhibited the PaTy tyrosinase activity which can be promptly restored through photocleavage of ONBY under UV-irradiated condition. The activated PaTy was demonstrated effectively oxidize l-tyrosine and tyramine-conjugated hyaluronic acid (HA_T) to form melanin particles and a hydrogel, respectively. For the generation of artificial melanin within living tissues, PaTy is packed into lipid nanoparticles to give PaTy-containing liposomes (PaTy_Lip), transdermal delivery of PaTy is achieved within ex vivo porcine skin and in vivo mouse skin tissues, thus allowing for the in situ biosynthesis of artificial melanin for enhancing skin tissue protection against UV irradiation. Similarly, Zhang et al.^269^ achieved optical control of protein activity for firefly luciferase, BRAF kinase, KRAS GTPase, and MEK1 kinase by genetically incorporation of photocaged aspartic acid ONBD and MeONBD via newly evolved PylRS mutants to replace functionally essential Asp residues in these proteins. The removal of protective groups from Asp in the luciferase, BRAF and KRAS showcased the ability to optically manipulate ATP-binding residue, the conserved D-F-G and D-T-A-G motifs, while decaging of photocaged Asp, serving as a mimic of phosphorylation, highlighted the capability to dissect the role of each phosphorylation site within MEK1. Also, Hauf et al.^270^ developed a new strategy leveraging GCE by engineering highly efficient aaRSs for the photocaged ncAA ortho-nitrobenzyl DOPA (ONB-DOPA). The engineered ONB-DOPA-specific aaRS allows for production of mussel adhesive protein (MAP) type 5 with multiple site-specific ONB-DOPA incorporations in *E. coli* cells, resulting in photocaged adhesives with spatiotemporal control. Additionally, Wolffgramm et al.^271^ presented a method for controlling human DNMT (DNA methyltransferase) catalysis within cellular contexts by substituting a catalytically crucial cysteine residue with photocaged cysteine DONBC, which enables the expression of mutant DNMTs in a temporarily inactive state, followed by their swift activation with light-decaging. Courtney et al.^272^ also introduced the development of a light-activated protein phosphatase MKP3 through two distinct approaches: introducing the DONBC into the active site to completely abolish catalytic activity until photoactivation and incorporating another caged lysine hydroxycoumarin lysine (HCK) into the kinase interaction motif to modulate the protein–protein interaction between MKP3 and ERK2 to abrogate the phosphatase–substrate interaction until photoactivation. Additionally, HCK was also used for targeting protein degradation through fast optogenetic activation, Ryan et al.^273^ developed a compact and light-responsive degron, referred to as optoDeg, which harnesses the N-end proteolytic pathway and incorporates a photocaged N-terminal HCK, which is directly linked to the protein of interest via a short 13-amino acid peptide, thus enabling rapid optical activation of protein degradation by prompting the recruitment of E3 ligases and subsequent proteasomal machinery.

Moreover, other photocaged lysine or cysteine analogues carrying nitrophenyl groups were also used to enables precise spatiotemporal control over kinase activity^274^ and light activation of fragment association and protein splicing in mammalian cells.^275^ Additionally, using another photocaged ncAA 2,3-diaminopropionic acid (pDAP) carrying nitrophenyl group, Eiamthong et al.^276^ engineered a novel polyethylene terephthalate (PET) hydrolase MG8 variant MG8(S171pDAP), conjected with a split GFP system, by replacing catalytic serine residue (S171) of MG8 with its isosteric amino analogue pDAP. After MG8(S171pDAP) was photodeprotected via UV illumination, the resulting MG8(S171DAP) was further engineered to effectively convert a PET hydrolase activity into a covalent binder for PET biofunctionalization, potentially serving as a valuable instrument in developing wearable sensors and plastic items that can identify disease biomarkers and therapeutic levels.^277,278^ Noteworthily, besides the above-mentioned direct-incorporation of photocaged ncAA into target proteins for photocontrollable regulation, an indirect way for photoswitchable regulation mediated by ingenious inhibitor conjugates with photoisomerizable linkers was also reported by Jason Chin’s group.^279^ They introduced a genetically encoded bioorthogonal ligand tethering (BOLT) for the precise inhibition of protein functions (iBOLT). In iBOLT, the inhibitor conjugate/protein pairs were made where target proteins were genetically incorporated by ncAAs (bicyclononyne-lysine BCNK or cyclopropane lysine CypK) with bioorthogonal alkyne or alkene group via GCE into permissive sites, while inhibitor conjugate was designed with complementary bioorthogonal tetrazine group, which reacts with alkyne or alkene group of ncAAs within target protein through a rapid and specific inverse electron demand Diels–Alder (IEDDA) reaction, thereby attaching small-molecule inhibitor to target protein for first rapid and specific inhibition of MEK isozymes. By incorporating photoswitchable linkers in the inhibitor conjugates, protein activity was subject to reversible and optical control in live mammalian cells, a strategy named as photo-BOLT. The iBOLT’s flexibility and scalability was also demonstrated in mammalian cells to selectively and rapidly inhibit lymphocyte specific kinase (LCK)^280^ which is distantly related with MEK by using a broad-spectrum kinase inhibitor conjugate.

##### 4.1.2.5 Chemical Control of Protein Function

Besides photodependent decaging, conditional activation of protein activity by small chemical molecule-dependent decaging is a valuable technique for synthetic biology applications. In order to manipulate protein activity chemically by liberating free lysine, researchers have focused toward allyl and propargyl groups, which are widely employed protective groups for alcohol/amine and carbonate/carbamate, and can be selectively removed by organometallic catalysts under mild or living conditions.^281−284^ For this purpose, Li et al.^285^ used lysine analogues (*N*^ε^-allyloxycarbonyl-l-lysine, AlocK; *N*^ε^-propargyloxycarbonyl-l-lysine, ProcK) with allyl or propargyl protective group, which can be incorporated into a targeted protein via GCE to replace the natural lysine at a key active site, so that the protein activity is in a repressive state. Through employing a palladium-mediated deprotection reaction, they realized in situ protein activation. Additionally, another two lysine analogues, azidobenzyloxycarbonyl lysine (PABK) and *trans*-cyclooctenyloxycarbonyl lysine (TCOK), containing caging groups like azidobenzyloxycarbonyl and cyclooctenyloxycarbonyl groups can be removed by introducing small molecules like phosphines (through Staudinger reduction, followed by self-immolation of the caging group to release lysine^286^) and tetrazines (through an IEDDA cycloaddition reaction, followed by self-immolation^287^), respectively. By substituting active site lysine residue of target proteins with PABK or TCOK, Brown et al.^288^ developed two distinct small molecule triggered lysine decaging systems, which were proved versatile for caging a range of enzymes. PABK relies on the Staudinger reduction of an aryl azide by phosphine (2DPBM), while TCOK involves a cycloaddition between a tetrazine/methyl tetrazine (MeTz) and a transcyclooctene. Also, Ngai et al.^289^ established a chemical control method called bioorthogonally activatable base editor (BaseBAC) that enables in situ and on-demand initiation of pyroptosis within specific cell types. Specifically, TCOK was genetically encoded to replace the protospacer adjacent motif (PAM)-interacting residue K1200 of Cas9, thereby blocking Cas9’s DNA-binding activity, which can be activated by 1,4-dimethyl-2,3,5,6-tetrazine (Me_2_Tz). The controllable Cas9 variant was further fused with deaminase APOBEC1, giving the resulting BaseBAC with the enzymatic activity of a cytosine base editor (CBE), which permits on-demand base editing on the GSDME gene, leading to the production of a truncated version of its N-terminal domain, which triggers pyroptosis.

On the other hand, chemical control can also be used for selective inhibition of protein isoform by small molecules. RAF isoforms are frequently mutated oncogenes that function as effector kinases in the RAS-ERK signaling cascade. The use of many RAF inhibitors has been associated with a phenomenon known as “paradoxical activation” of RAF kinase activity due to the transactivation of the CRAF protomer.^290,291^ Morgan et al.^292^ employed the BOLT approach^279^ to selectively target inhibitors to CRAF. Leveraging the available RAF-selective pharmacophores, they created two BOLT ligands: AZ13-tet containing the AZ628 pharmacophore and AZ181-tet containing the PLX4720 pharmacophore, which are significantly distinguished from the previous iBOLT ligands^279^ with photoisomerizable linkers. CRAF at position S357 or Q436 was replaced by BCNK bearing a bioorthogonal alkyne group, which reacts with the two tetrazine group-containing BOLT ligands through IEDDA reaction, the small-molecule pharmacophore inhibitors then bind to target protein for specific inhibition. This selective CRAF inhibition was also demonstrated to promote paradoxical activation and showed the potential of BOLT as a valuable tool for assessing potential drug targets before the discovery of target-specific small molecules. In addition, due to the inability of chemical decaging to regulate protein function in a reversible manner, Cao et al.^293^ developed a versatile supramolecular host–guest recognition strategy that allows for the reversible control of protein function. By genetically encoding 4-*tert*-butyl-l-phenylalanine (tBuF) equipped with a guest side chain into functional surface sites of active proteins in proximity to active sites, the resulting active proteins with a guest side chain can be recognized and by a host molecule cucurbit[7]uril (CB[7]), thereby effectively blocking their functional binding surface and inhibiting their enzyme activity. Importantly, protein activity can be reactivated through treatment with a competitive guest molecule l-phenylalanylglycylglycine (FGG). Similarly, Zubi et al.^294^ combined computational and experimental techniques to realize reversible activation by developing a metal-responsive chemical switch into proteins by genetically encoding pairs of BpyAla residues, which contain the bipyridine (Bpy) groups (linking groups, LGs), into two distinct enzymes, a serine protease (prolyl oligopeptidase, POP) and firefly luciferase. Due to the bis-bidentate metal binding by the Bpy LGs, the BpyAla incorporation enables metal coordination to influence the enzyme conformation of these variants, leading to reversible activation of their activity in the presence/absence of metal ions.

#### 4.1.3 Biocontainment

In addition to the modification of intracellular details such as gene expression and enzyme engineering, GCE technology has found successful application in the broader realm of organismal engineering. On the one hand, these engineered organisms can utilize GCE technology more efficiently; on the other hand, these novel organisms also possess numerous characteristics not found in typical organisms. As mentioned previously, researchers avoid the impact of the genetic material of engineered bacteria on the natural environment through the redistribution of genetic codes.

An alternative approach to avoid contamination resulting from genetically modified organisms (GMO) involves engineering them to thrive exclusively under specific conditions, such as in the presence of particular ncAAs. For example, Rovner et al.^196^ systematically introduced in-frame TAG codons into multiple essential genes within the genomic framework of C321.ΔA. This genetic modification yielded auxotrophic strains whose growth became reliant on the presence of either *p*-iodo-l-phenylalanine (pIF) or *p*-azido-l-phenyalanine (pAzF). One notable strain, denoted as rEc.γ.dC.46′.ΔtY, featured three TAG codons inserted into conserved functional residues within the *MurG*, *DnaA*, and *SerS* genes with targeted tRNA deletions. This engineered strain not only maintained robust growth but also exhibited undetectable escape frequencies. Subsequent investigations^295^ indicated that physical containment introduced an additional level of protection for such microbes.

Recently, Chang et al.^296^ extended the application of biocontainment strategies based on ncAAs to a eukaryotic system by integrating an orthogonal translation system to incorporate OMeY into essential proteins in budding yeast. Followed by understanding escape mechanisms that could potentially undermine biocontainment, they optimized their strategy by introducing mechanisms to counteract the emergence of amber suppressor tRNAs and developing both transcriptional and translational control switches for biocontainment. Through a fitness-oriented screening method, they identified optimal sites for OMeY incorporation that ensure robust growth and negligible escape frequency of the yeast strains.

In these investigations, strain viability was contingent upon the successful translation of crucial proteins, while in other studies, the functionality of key proteins relied on the characteristics of ncAAs. For instance, Mandell et al.^197^ computationally redesigned the cores of essential enzymes, such as tyrosyl-tRNA synthetase and adenylate kinase, to necessitate the presence of l-4,4′-biphenylalanine (bipA) for correct translation, proper folding, and functional activity. The combination of multiple redesigned enzymes in C321.ΔA culminated in the generation of a bipA-dependent strain designated as DEP that exhibited markedly reduced escape frequencies. Even following an extended period of automated continuous evolution, DEP strains consistently demonstrated a complete absence of observable escape events and maintained effective containment without causing detrimental effects upon introduction into mammalian cell cultures.^297^ These results signify the efficacy of DEP both over extended time frames and within various experimental contexts. In 2016, Tack et al.^298^ engineered TEM-1 β-lactamase to be reliant on 3-nitro-l-tyrosine (3nY) or 3iY, thereby leading to the development of diverse bacterial strains dependent on ncAAs. Moreover, organisms that are dependent on various ncAA attributes, such as orthogonal protein–protein interface,^299,300^ PTM^301^ and metal chelation,^302^ have also been developed. These strains were not only suitable for biocontainment, but also possessed the capacity for screening and evolving synthesis pathways to produce corresponding ncAAs.^190^

### 4.2 Biomedicine

#### 4.2.1 Conjugates and Antibodies

**Figure 11:**
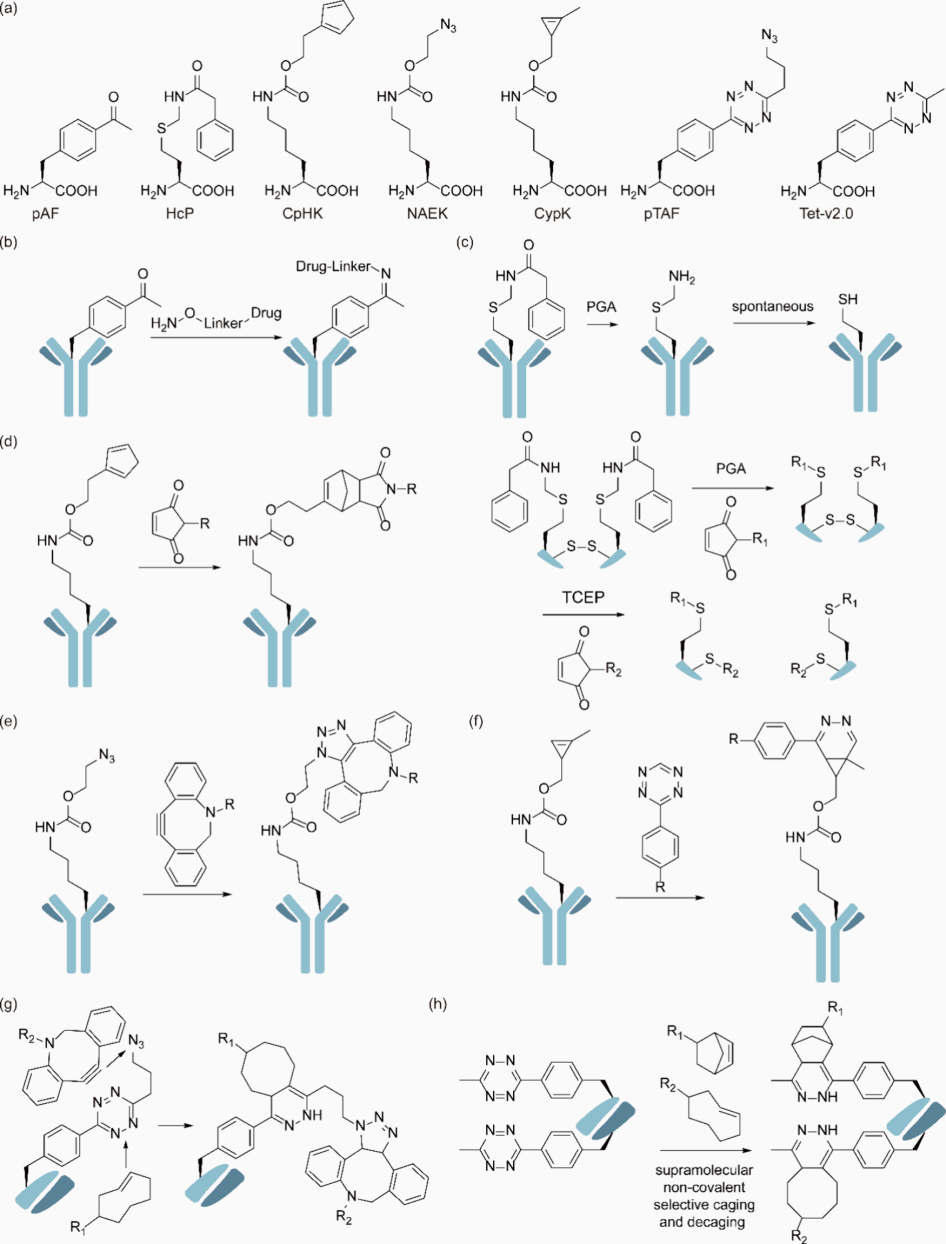
Protein conjugates. (a) Chemial structures of commonly used ncAAs for bioconjugation. (b) Reaction on the incorporated ncAA pAF for bioconjugation. (c) Reaction on the incorporated ncAA HcP for bioconjugation (upper) and dual-bioconjugation (lower). PGA, penicillin G acylase. For protein with cystine and HcP, PGA can deprotect HcP and the subsequent TCEP can activate cystine, enabling sequential labeling of the protein. TCEP, tris(2-carboxyethyl)phosphine. (d) Reaction on the incorporated ncAA CpHK for bioconjugation. (e) Reaction on the incorporated ncAA NAEK for bioconjugation. (f) Reaction on the incorporated ncAA CypK for bioconjugation. (g) Reaction on the incorporated ncAA pTAF for bioconjugation. (h) A brief schematic of the strategy to prepare protein dual conjugates with ncAA Tet-v2.0. Tetrazine residue positions could be computationally designed so that exposed residues could be reversibly caged by a barrel-shaped supramolecular host, whereas semiburied residues that were resistant to caging retained their reactivity, thus allowing sequential IEDDA labeling.

Antibody–drug conjugates (ADCs) are biopharmaceutical drugs designed to enhance the therapeutic window against cancer, with 11 ADCs approved by the FDA.^303^ Traditional ADC production methods involve nonselective conjugation to lysine or cysteine residues, leading to product heterogeneity. This heterogeneity negatively impacts the pharmacokinetics, tolerability, and efficacy of ADCs, making them less desirable.^304^ Additionally, the limited number of available conjugation sites on antibodies poses challenges for scaling up production. Bispecific antibodies, engineered to recognize two distinct epitopes simultaneously, face similar challenges due to low fidelity in assembling heavy and light chains through chemical conjugations at lysine or cysteine residues. Despite some successful efforts, GCE strategies offer particularly promising solutions.^305,306^ GCE enables the site-specific incorporation of reactive amino acids, facilitating the production of homogeneous ADCs and bispecific antibodies. Several ncAAs and strategies have been employed for various tasks in this context (Figure 11a).

One strategy is to use ncAAs to directly conjugate with reagents, thus modifying the antibody with a single kind of molecule. For example, *p*-acetylphenylalanine (pAF) is commonly used to form a stable oxime bond with amino-oxy-modified drugs to build antibody–drug conjugates and bispecific antibodies (Figure 11b);^307,308^ (*S*)-((2-phenylacetamido)methyl)-l-homocysteine (HcP) can be enzymatically deprotected to homocysteine (Hcy) for bioconjugation(Figure 11c);^309^ cyclopentadiene derivative of lysine (CpHK) enables the conjugation between antibody and maleimide-modified drug (Figure 11d).^310^ It is worth mentioning that ADC synthesis increasingly leverages rapid and selective bioorthogonal reactions to enhance process efficiency.^311^ The strain-promoted azide–alkyne cycloaddition (SPAAC) on an azido group-bearing amino acid *N*^ε^-2-azidoethyloxycarbonyl-l-lysine (NAEK) and the inverse electron demand Diels–Alder (IEDDA) reaction on an alkenyl group-bearing amino acid *N*^ε^-[((2-methylcycloprop-2-en-1-yl)methoxy)carbonyl]-l-lysine (CypK) are used to prepare site-specific antibodies (Figure 11e,f).^312,313^

Other strategies aim at producing dual-modified antibody derivatives. Wang et al. designed 4-(6-(3-azidopropyl)-s-tetrazin-3-yl) phenylalanine (pTAF) containing mutually orthogonal and bioorthogonal azide and tetrazine reaction handles to enable one-pot reactions with combinations of reagents for multifunctional antibody multiconjugates (Figure 11g).^314^ The sequential IEDDA reaction strategy was also developed. This strategy involves computational-aided design to select a fully exposed site and a semiburied site to incorporate two tetrazine amino acids into one protein. The selective caging of tetrazine amino acid reactivity by a barrel-shaped supramolecular host achieves dual-labeling of antibody with two different IEDDA reactions (Figure 11h).^315^

#### 4.2.2 Tuning Pharmacokinetic and Pharmacodynamic Properties of Protein Drugs

Protein-based therapeutics have become a significant part of medicine market due to their high potency, high specificity, low side effects, and versatility. Besides high therapeutic potency, to function as effective drugs, protein-based drugs must have high stability, cell permeability, and appropriate pharmacokinetics and pharmacodynamics.^316^ However, due to inherent susceptibility of proteins, protein-based drugs tend to have a short blood circulation time and concomitant loss of activity.^317^ Two common approaches to extending the short blood half-life of protein therapeutics involve attaching polyethylene glycol (PEG) to protein surfaces and improving the affinity between serum proteins and protein drugs. However, both methods face hurdles of selective homogeneous and scalable conjugation, as well as the maintenance of protein bioactivity. GCE technology offers an opportunity to overcome these challenges through the incorporation of ncAAs, providing a highly selective, homogeneous, and scalable conjugation reaction for controllable cross-linking while maintaining protein bioactivity.^317^

**Figure 12:**
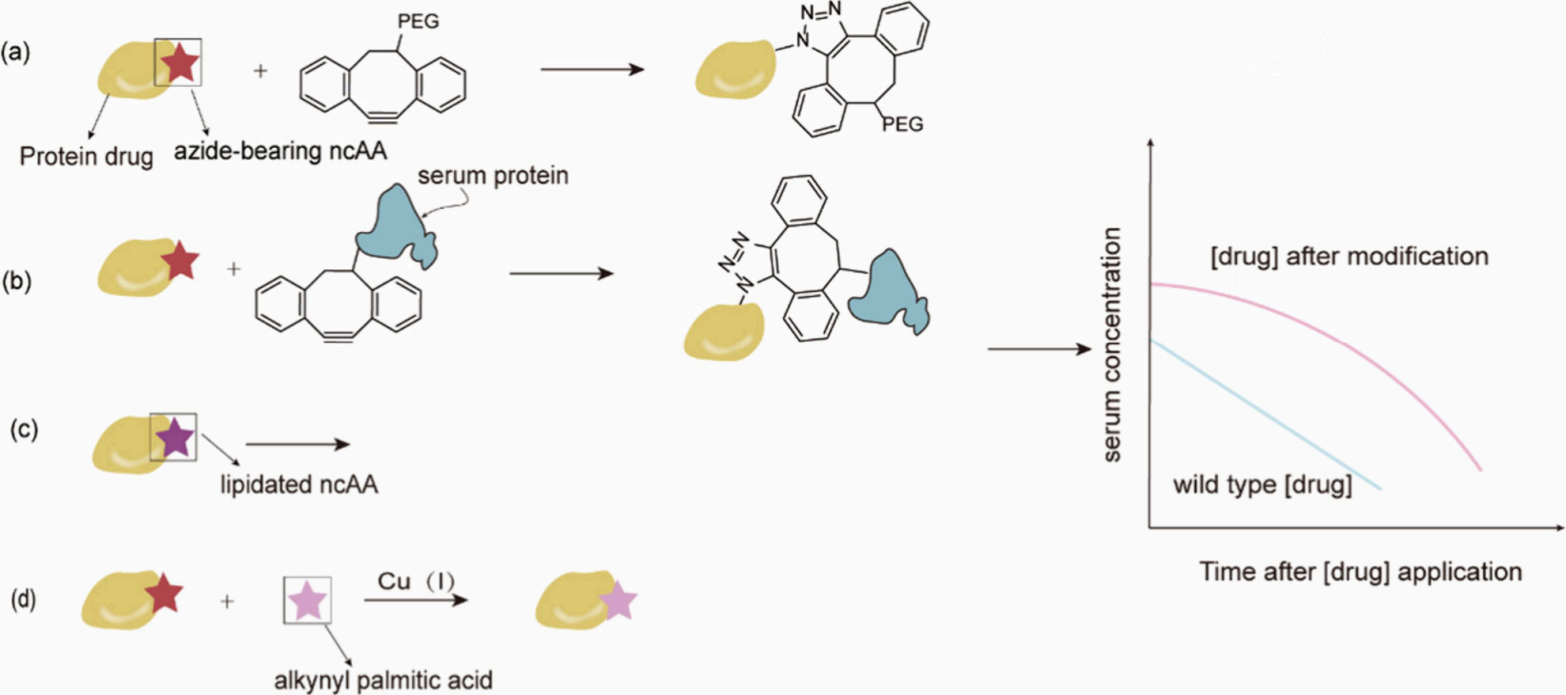
Applications of the GCE technology using azide groups or lipidated amino acids for site-specific PEGylation or lipidation to tune the pharmacokinetic properties of proteins. (a) Genetically incorporated azide-bearing ncAA enables site-specific PEGylation, increasing the half-life of protein drugs while retaining their bioactivity. (b) Genetically incorporated azide-bearing ncAA allows for the construction of serum protein-drug conjugates, prolonging the serum half-life of the proteins. (c) Genetically incorporated fatty acid-containing ncAA facilitates site-specific fatty acid decoration, extending the protein’s half-life. (d) Genetically incorporated azide-bearing ncAA enables site-specific lipidation, resulting in a tailored half-life.

PEGylation is a widely adopted strategy to enhance various pharmaceutical aspects of protein therapeutics, including the stability, pharmacokinetics, and efficacy of the bioconjugate. However, it encounters significant challenges, including the production of heterogeneous conjugates and immunological side effects. GCE technology addresses these issues by enabling precise cross-linking positions for proteins, thereby overcoming the problem of nonspecific conjugation. The site-specific PEGylated interleukin-4 (IL-4) has an approximately 6-fold improvement in terminal half-life from 0.76 to 4.7 h as compared with unmodified IL-4 and above 3-fold improvement in the total drug exposure across time. In addition, the conjugates retained bioactivity and showed targeting properties. This conjugation is achieved by two steps, including the incorporation of azide-bearing amino acids at desired sites through GCE technology and the subsequent attachment of PEG moieties through a bioorthogonal click reaction (Figure 12a).^318^ Similarly, via the incorporation of NAEK, various precise PEGylations were introduced into interleukin-2(IL-2), facilitating the exploration of the effect of PEG sizes, different cross-linking sites, and the number of PEG moieties. One variant with dual 20 kDa PEG modification at the Tyr 31 and Thr 51 sites of IL-2 presented superior properties. This variant not only showed significant pharmacokinetic enhancements and an increased half-life in vivo compared to the wild type, but it also exhibited remarkable therapeutic efficacy with minimal adverse effects.^319^ Similar effect was also observed in human interferon-α2a(IFN-α2a) variants decorated with different sizes of PEG moieties or liner polyglycerol by an azido functionalized IFN-α2a.^320^

Incorporation of ncAAs can also tune the affinity of serum proteins and drugs, therefore enhance the pharmacokinetic and pharmacodynamic properties. A direct serum albumin and protein conjugate was constructed by incorporation of AzF which prolongs the serum half-life of proteins (Figure 12b).^321^ In addition, the production of fatty acids decorated protein therapeutics via genetical encoding ncAAs with increased binding affinity to serum proteins have also been reported. To enhance the blood circulation time of therapeutic proteins in vivo, Caiyun Fu introduced a fatty acid-containing amino acid ε-*N*-heptanoyl-l-lysine into glucagon-like peptide-1(GLP-1). Compared with wild-type GLP-1, the conjugate demonstrated improved binding affinity toward serum albumin, and therefore had extending half-lives (Figure 12c).^322^ More recently, AzF was genetically incorporated into human interleukin-6(IL-6). Subsequently, through chemical cross-linking reactions, site-specific lipidation of IL-6 was constructed, resulting in approximately 30fold extended terminal half-life.^323^ To address the need for tailor the half-life of protein drugs, multiple instances of AzF were introduced to protein–polymer fusions and then attached to alkynyl palmitic acid using click-chemistry (Figure 12d). The AzF residues facilitate precise and controlled functionalization with fatty acids by precisely control the position and number of incorporated ncAAs. Incorporating a single instance of the ncAA remarkably limited the half-life scope of protein drugs but multiple instances of ncAAs incorporation break the limitation. Various instances of AzF were introduced to protein–polymer fusions and then attached to alkynyl palmitic acid through chemical ligation. Different instances of AzF contributed to varied binding affinity between protein complex and serum albumin, resulting a tailored half-life.^317^ Overall, GCE technology has provided a powerful tool to applied in improving pharmacokinetics and pharmacodynamics.

#### 4.2.3 On-Demand Activation of Proteins

**Figure 13:**
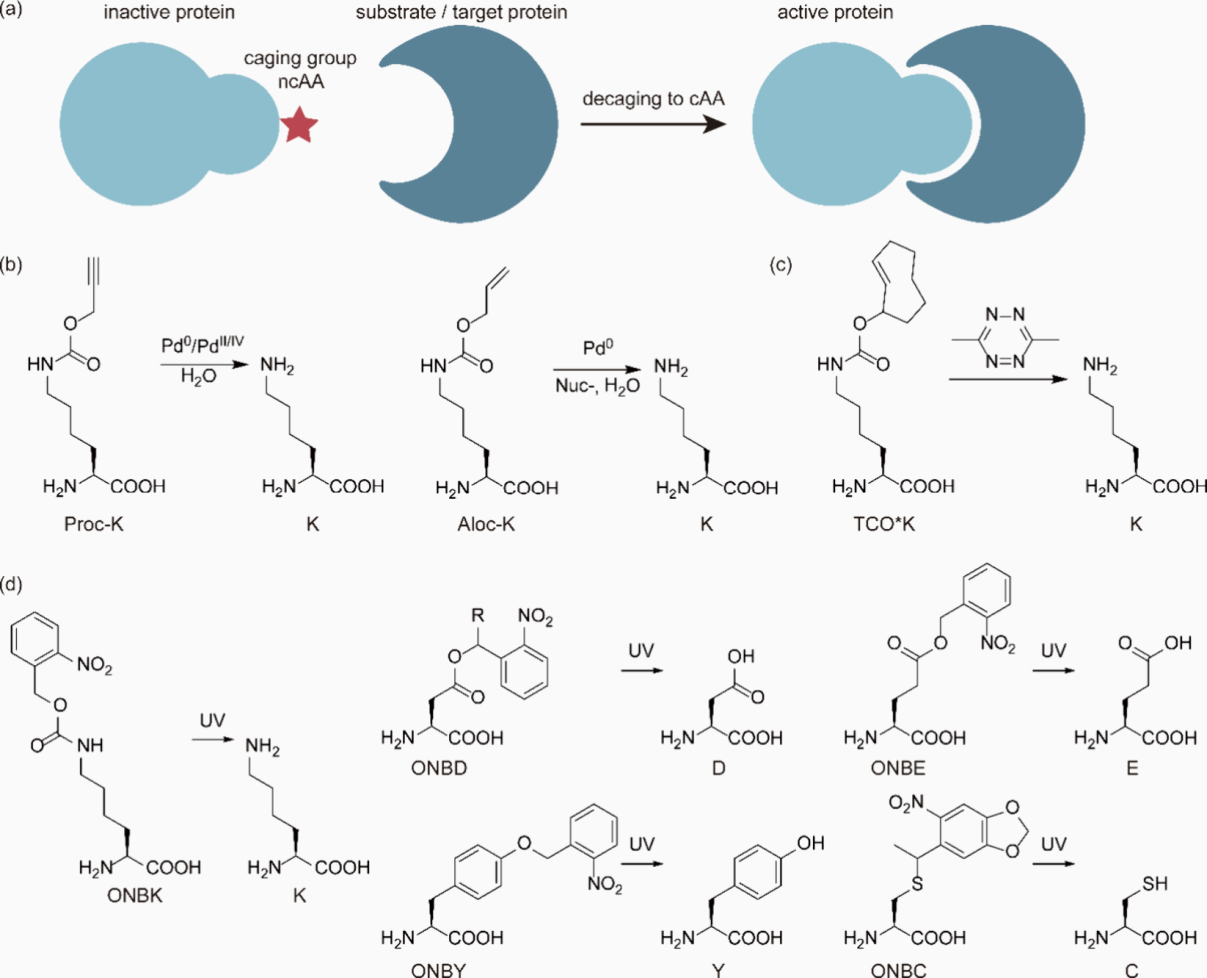
On-demand activation of proteins. (a) Strategy of on-demand activation of proteins. The ncAA with caging group is incorporated at a key site to inactivate the protein. With decaging methods, the ncAA becomes cAA and activate the protein. cAA, canonical amino acid. (b) Reactions for Proc-Lys and Aloc-Lys to be decaged to lysine by Pd catalyst. (c) Click-to-release reaction for TCO*K to be decaged to lysine by tetrazines. (d) Reactions for ncAAs with nitrobenzyl-based photolabile protecting groups (PPGs) be decaged to cAA.

Methods for on-demand activation of proteins enable targeted study and manipulation of proteins by researchers and clinicians. These methods use an active protein as the active pharmaceutical ingredient linked to a pro-moiety via a labile chemical bond, allowing for specific and efficient delivery to the intended target (Figure 13a).^324^ Over the past decade, several ncAAs with protective groups that can be intentionally cleaved have been developed to activate therapeutic proteins. These include chemical or photoactive analogues of lysine, tyrosine, glutamic acid, and aspartic acid, used in prodrug designs of enzymes, protein toxins, and antibodies.

One type of these amino acids requires chemical activation. One example is *N*^ε^-propargyloxycarbonyl-l-lysine (Proc-K) and *N*^ε^-allyloxycarbonyl-l-lysine (Aloc-K), which bear caging groups cleaved by palladium catalysts to replace key lysines in enzymes (Figure 13b).^285^ Another example is *N*^ε^-(((*trans*-cyclooct-2-en-1-yl)oxy)carbonyl)-l-lysine, TCO*K, which undergoes a click-to-release bioorthogonal reaction and has achieved enzyme activation in vivo (Figure 13c).^289^

The other type of these amino acids requires photo activation via their nitrobenzyl-based photolabile protecting groups (PPGs) (Figure 13d). One example is *o*-nitrobenzyl-*o*-tyrosine (ONBY), which converts back to tyrosine upon UV light exposure, allowing rapid and temporal protein activation.^325^ ONBY was used to activate protein kinase, lethal factor and antibody.^326,327^ Other photocaged amino acids developed include analogues of glutamic acid (ONBE), aspartic acid (ONBD), cysteine (ONBC), and dihydroxyphenylalanine (DOPA).^267,269,270,328^ Combining this prodrug strategy with other genetic code expansion strategies can enhance prodrug therapeutics. For example, Bridge et al. used ONBY and *p*-benzoyl-l-phenylalanine (pBpA) on an antibody to make it a photoactivable covalent antibody.^329^

On-demand activation of proteins using ncAAs enables precise spatial and temporal control over protein function, enhancing the specificity and efficiency of therapeutic interventions. Future advancements combining chemical and photoactive ncAAs with genetic code expansion strategies hold great potential for developing next-generation biopharmaceuticals with unprecedented precision and functionality.

#### 4.2.4 Development of Covalent Protein Drugs by Proximity-Enabled Cross-Linking Amino Acids

Nearly 30% of currently available drugs targeting enzymes use a covalent mechanism. There is an increasing trend in utilizing targeted covalent inhibition to address challenging targets that are difficult to engage with noncovalent drugs.^330^ However, the development of small molecular covalent inhibitors is often hampered by potential off-target toxicity and limited interaction surfaces.^331^ Similarly, covalent protein drugs face challenges due to their reliance on cysteine for linkage and lack of covalent reactivity with natural residues of targets.^332^ Covalent protein drugs incorporating ncAAs for cross-linking offer a promising solution, providing precise recognition, a wide selection of reactive amino acids, and diverse incorporation sites. Recent developments in proximity-enabled or photocaged cross-linking amino acids, as we systematically summarized in the section 4.3.1.3, have opened new opportunities for covalent protein drug development.^333^ In this section we highlight current promising covalent protein drugs.

**Figure 14:**
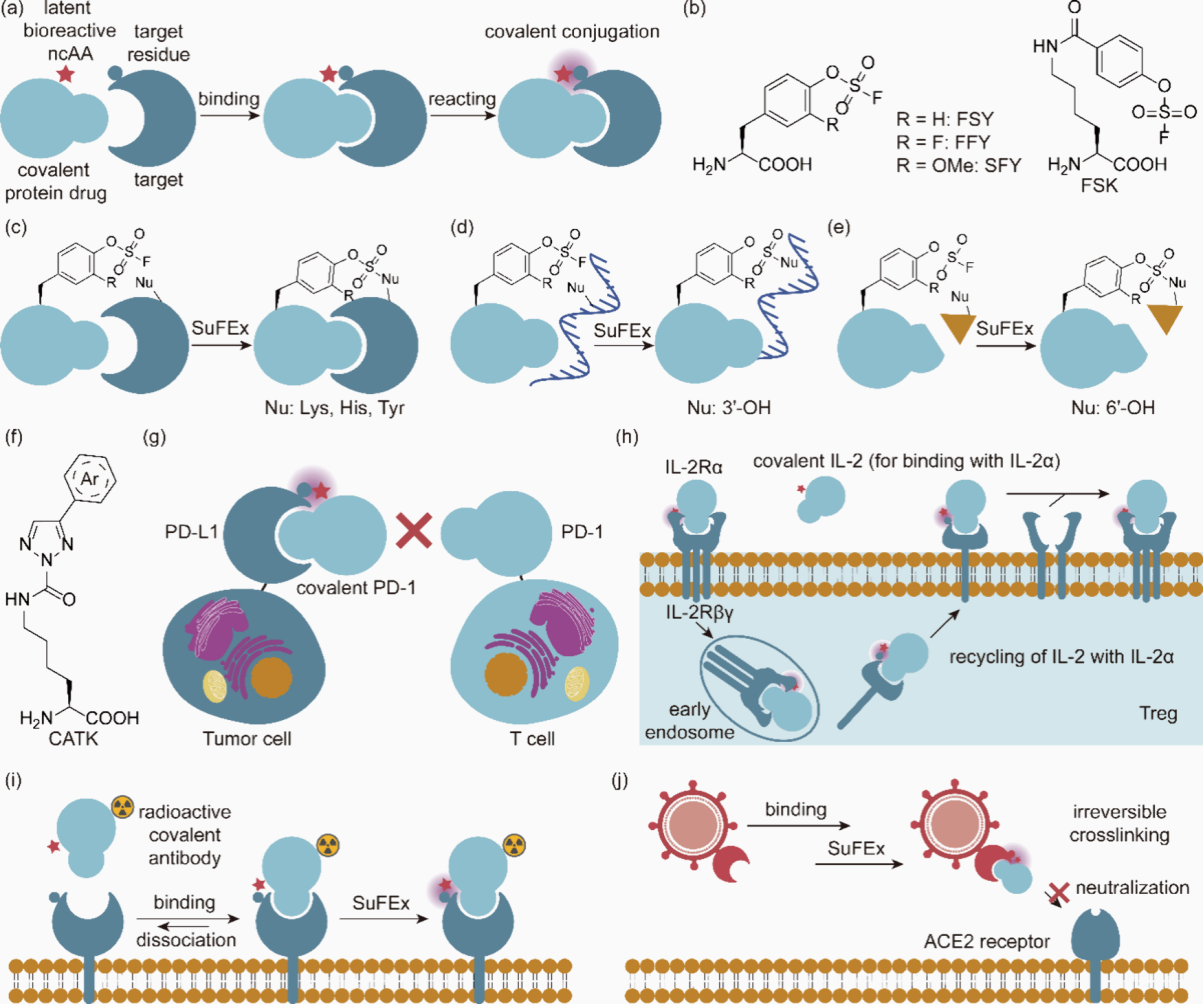
Covalent protein drugs. (a) Strategy of covalent protein drugs based on proximity-enabled protein cross-linking. The latent reactive ncAA on protein drug can conjugate with target residue on the drug target once the two proteins bind together. (b) Chemical structures of SuFEx-based amino acids. (c) Proximity-enabled protein cross-linking based on SuFEx. The ncAAs can react with Lys, His, and Tyr on the target protein. (d) Proximity-enabled cross-linking with RNA based on SuFEx. The ncAAs can react with 3′-OH on the RNA. (e) Proximity-enabled cross-linking with carbohydrate based on SuFEx. The ncAAs can react with 6′-OH on the RNA. (f) Other amino acids for proximity-enabled cross-linking. The aromatic ring can be varied. (g) Strategy of covalent PD-1. Covalent PD-1 can conjugate with PD-L1 on tumor cells, which prevents PD-L1 to bind with PD-1 on T cells and inhibit T cell activity. (h) Strategy of covalent cytokine IL-2. FSY-bearing IL-2 variants covalently bind to IL-2Rα when in proximity, resulting in persistent recycling of IL-2 and selectively promoting the expansion of Tregs but not effector cells. interleukin-2, IL-2. Treg, regulatory T cell. (i) Strategy of covalent antibody conjugates. The radioactive antibody can covalently bind with receptor, increasing radioisotope levels and extending tumor residence time. (j) Strategy of covalent antibody. The covalent antibody can covalently bind with receptors on virus, preventing the binding between virus and ACE2 on target cells. ACE2, angiotensin-converting enzyme 2.

One groundbreaking research is the platform technology, proximity-enabled reactive therapeutics (PERx) developed by Li et al. (Figure 14a).^334^ This platform introduces a latent bioreactive ncAA into a protein drug at or near the binding interface. The protein–drug interaction then enables selective cross-linking of the ncAA with a proximal residue of the target protein, RNA, or carbohydrate, allowing the protein drug to covalently bind to the target and elicit therapeutic effects (Figure 14c,d,e). The fluorosulfate-l-tyrosine (FSY) has a latent chemical reactivity based on sulfur-fluoride exchange (SuFEx) reactions and remains inert inside the protein and in vivo (Figure 14b,c).^335^ FSY incorporated into programmed cell death protein 1 (PD-1) can react with a proximal histidine of PD-L1, thereby forming a covalent linkage that enhances T cell and CAR-T cell activation and inhibits tumor growth more effectively than noncovalent PD-1 or anti-PD-L1 antibody (Figure 14g). FSY was also used to produce a radiolabeled covalent antibody, which significantly augmenting the efficacy of radio therapy (Figure 14i), and a covalent interleukin-2 (IL-2) to enhance and prolong Treg activation to suppress inflammatory and autoimmune responses in mouse models (Figure 14h).^336,337^

To improve the reactive capabilities of proximity-enabled reactive amino acids, Yu et al. developed fluorine-substituted fluorosulfate-l-tyrosine (FFY), which exhibited faster cross-linking kinetics than FSY. FFY-containing nanobodies demonstrated increased potency in neutralizing SARS-CoV-2 (Figure 14b,j).^338^ Additionally, *o*-sulfonyl fluoride-*O*-methyltyrosine (SFY), developed to enlarge the reaction area, can cross-link with carbohydrates and enhance cancer cell killing by NK cells through irreversible cloaking of sialoglycan (Figure 14b,e).^339^ Liu et al. developed fluorosulfonyloxybenzoyl-l-lysine (FSK) to produce covalent nanobodies that irreversibly bound to epidermal growth factor receptors (EGFR) on cells.^340^

Proximity-enabled cross-linking amino acids also refine protein drug structures. Xu et al. encoded *N*^2^-carboxy-4-aryl-1,2,3-triazole-lysines (CATKs), which permits a spontaneous, quantitative, site-specific interstrand cross-linking with a proximal Tyr in a small antibody-mimetic protein (Figure 14f).^341^ This technology paves the way for designing novel protein structures with orthogonal cross-links, potentially leading to new applications in protein drugs.

The use of ncAAs for proximity-enabled cross-linking in covalent protein drugs represents a significant advancement in therapeutic development. This approach offers higher selectivity, enhanced efficacy, and broader applicability in targeting challenging biological systems. Future research may further optimize these strategies, expanding their use in diverse therapeutic areas.

#### 4.2.5 Drug Screening

**Figure 15:**
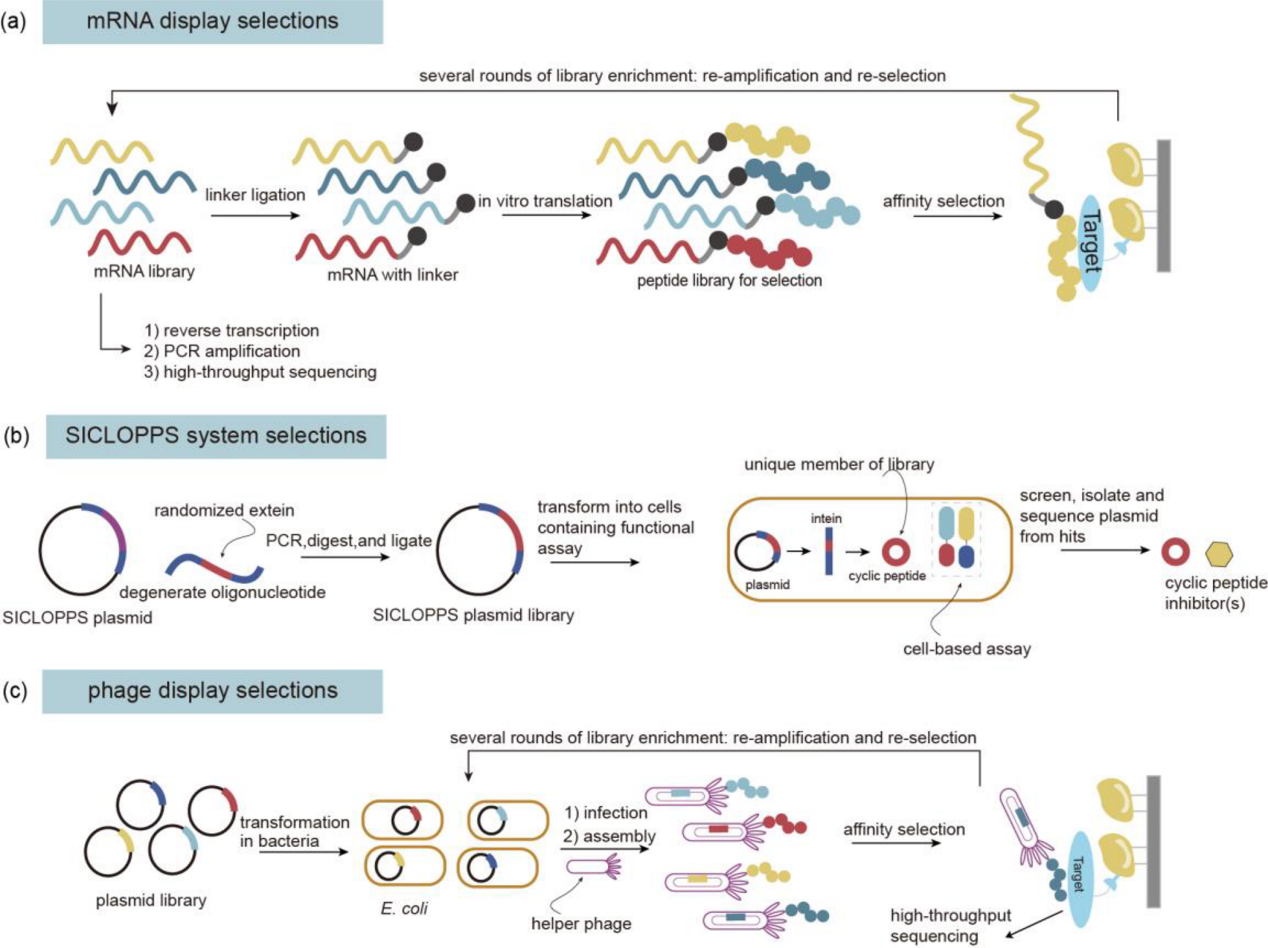
Comparison of mRNA display, SICLOPPS system, and phage display selections. (a) For mRNA display selections, an initial mRNA library is fused to a linker through various techniques. During in vitro translation, this results in the peptides being covalently attached to their corresponding mRNA coding tags. Affinity selection is then performed, followed by iterative rounds of mRNA/cDNA tag recovery, library reamplification, and reselection until sufficient enrichment is achieved. (b) To generate and screen a SICLOPPS library, start with standard molecular biology techniques to create the library using a degenerate oligonucleotide. This oligonucleotide is designed to specify the ring size of the cyclic peptides, the number of randomized amino acids, and any fixed amino acids. The resulting plasmid library is then transformed into cells equipped with an assay (e.g., FRET, life/death, phenotypic) for screening. Active cyclic peptides are identified by isolating the SICLOPPS plasmids from cells exhibiting the desired phenotype, followed by DNA sequencing to determine their identities. (c) Phage display selections start by transforming a bacterial host with a plasmid library. These bacteria are then infected with helper phages, leading to the production of a phage library where library peptides are displayed on phage coat proteins. Similar to mRNA display selections, this process involves affinity selection followed by multiple cycles of reamplification and reselection until the desired enrichment level is reached.

GCE technology has extensive applications in drug screening by incorporating ncAAs into proteins, thereby creating more diverse peptide and protein libraries. Here are some specific applications of GCE in drug screening: cyclic peptide libraries, antibody screening. and fragment-based drug discovery. Considering the growing significance of macrocyclic peptides as research tools and potential therapeutic agents, peptide drug-screening methods like phage display,^342,343^ mRNA display,^344,345^ and split-intein circular ligation of peptides and proteins (SICLOPPS)^346^ (Figure 15) gradually advance, the process of encoding diverse peptide libraries for discovery of bioactive macrocycles has also become increasingly convenient.^347^ Importantly, in view of the successful incorporation of many electrophilic ncAAs into proteins through GCE,^335,348−350^ it is possible to use them to create genetically encoded cyclic-peptide libraries. For example, Iskandar et al.^351^ presented evidence that the promiscuous ORS, p-CNF-RS^352^ can effectively incorporate ncAAs at amber codons during in vitro translation (IVT) as a means to mRNA display library expansion. Utilizing the novel ncAA substrate *para*-cyanopyridylalanine (*p*-CNpyrA) for p-CNF-RS, they created pyridine-thiazoline (pyr-thn) bridged macrocyclic mRNA display libraries peptide by addition of deformylase (PDF) and methionine aminopeptidase (MAP) to remove formyl-methionine (fMet) and display N-terminal cysteine for cyclization^353^ with subsequent oxidation by thiazoline oxidase ThcOxi.^354,355^ The Pyr-thn-based selections targeting the deubiquitinase USP15 resulted in the identification of a nanomolar macrocyclic binder SEI144, which exhibits good selectivity for USP15 and its closely related homologues, opening the door for potential development as a chemical probe or deubiquitinase-recruiting ligand. An alternative approach for generating cyclic peptide libraries is known as SICLOPPS, a method leveraging circularly permuted inteins that self-catalyze the cyclization of a target peptide within cells. Young et al.^356^ employed the SICLOPPS system conbined with GCE to evolve cyclic peptide inhibitors of HIV protease in *E. coli* cells. Specifically, a diverse library of cyclic hexapeptides containing benzoylphenylalanine (*p*BzF) was first generated using the SICLOPPS expression system, and a subsequent selection process was undertaken to identify cyclic peptides capable of inhibiting HIV protease based on an electrophilic aryl ketone group of *p*BzF potentially to form a covalent Schiff base adduct with nucleophilic lysine (Lys) residues found in the HIV protease enzyme. After two rounds of selection, eight of twelve resulting isolated cyclic peptides were incorporated by *p*BzF. The most effective of these peptides (G12: GIXVSL; X = pBzF) was able to inhibit HIV protease by forming a covalent Schiff base adduct between the *p*BzF residue and the ε-amino group of Lys14 on the protease. Similarly, based on the SICLOPPS system, genetically encoded warhead-bearing ncAA 2-amino-8-oxodecanoic acid (AODA) via GCE as well as a yeast two-hybrid technique, Kang et al.^357^ also developed another versatile strategy for a yeast-based colorimetric screening platform termed as custom-designed warhead-armed cyclic peptide screening (CWCPS) platform to discover cyclic peptide inhibitors. AODA possesses the distinctive aliphatic group (8-oxo-decanoyl) of apicidin (a natural cyclic peptide for histone deacetylase (HDAC) inhibition) with an ethylketone moiety, which can strongly bind to Zn^II^ ion occupying HDAC’s active site cavity, thereby functioning as a warhead. Once the SICLOPPS split intein is spliced out, the intein fragment I_N_ separates from the recombinant protein, resulting in a lariat peptide attached to the GAL4 activation domain (AD). When a cyclic peptide equipped with the warhead binds to a target protein linked to the GAL4 DNA binding domain (DBD), it triggers the transcription of the *lacZ* reporter gene, leading to the display of a colorimetric phenotype and discovery of a highly potent cyclic peptide inhibitor CY5–6Q for the oncogenic target HDAC8.

**Figure 16:**
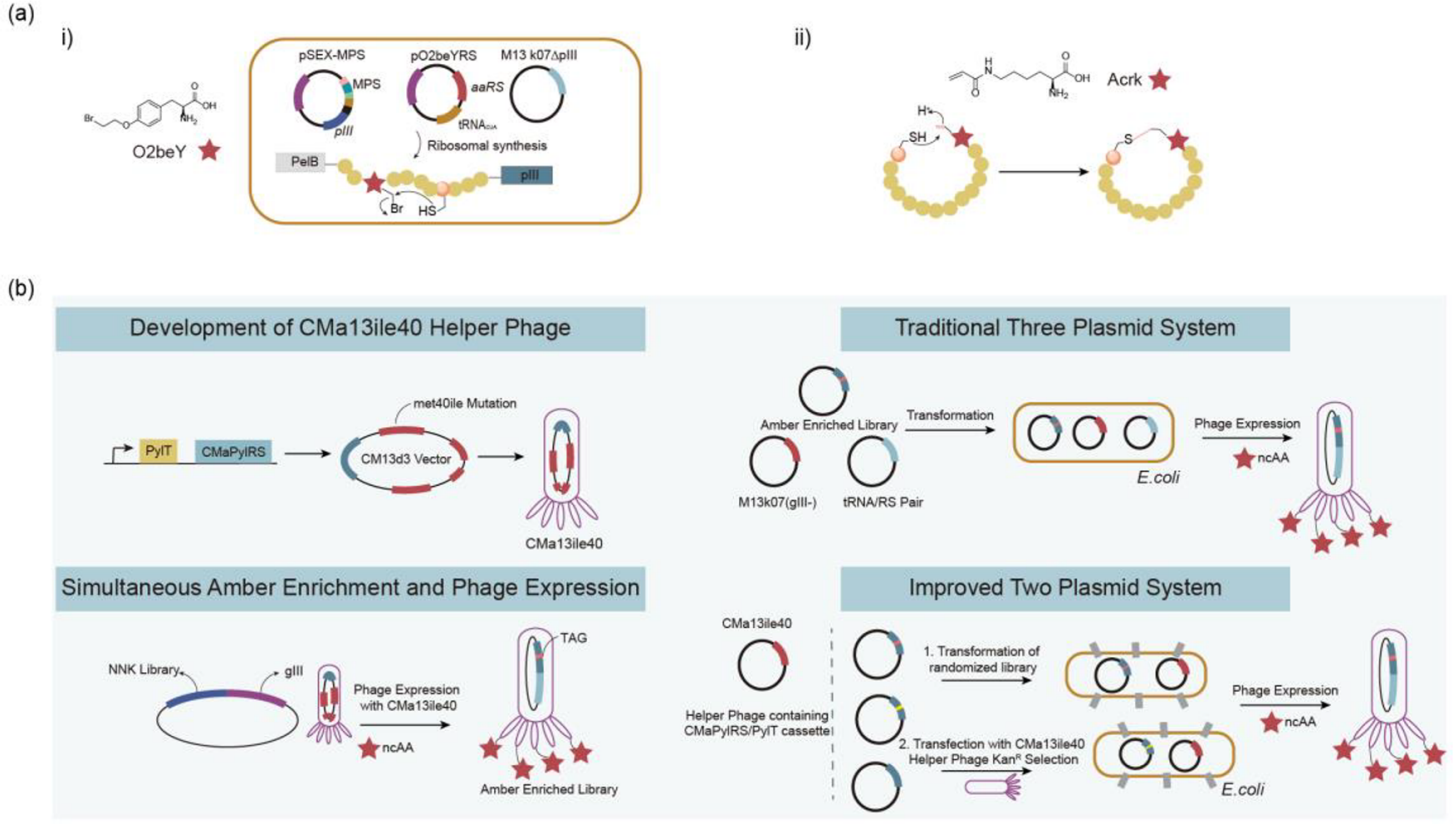
Application and optimization of phage display. (a) Genetically encoded, phage-displayed ncAA-containing cyclic peptides. (i) Cysteine-reactive O2beY and a proximal cysteine residue, resulting in the formation of macrocyclic peptides constrained by a nonreducible, interside-chain-to-side-chain thioether linkage. (ii) Cyclization of phage-displayed peptides through Michael addition between a cysteine and a genetically incorporated AcrK. (b) An amber-encoding helper phage for more efficient phage display of ncAAs. Comparison of the traditional three-plasmid expression system with the newly designed two-plasmid system for expression of phage libraries containing ncAAs.

Compared with mRNA display and SICLOPPS methods, phage display method offers benefits of being more cost-effective and simpler to operate, making it the most widely used display technique for generating and screening libraries of cyclic peptides in drug discovery.^358,359^ In efforts toward developing methods for generation and functional screening of phage displayed peptide macrocycles, Rudi Fasan’s team previously established techniques for producing peptide-based macrocycles (also known as macrocyclic organo-peptide hybrids, MOrPHs) by cyclizing ribosomally synthesized polypeptides using genetically encoded ncAAs.^360−364^ Notably, they have created macrocyclic peptides featuring a nonreducible, interside-chain-to-side-chain thioether linkage by achieving a cross-linking reaction between a genetically encoded alkyl bromide-bearing ncAA *O*-(2-bromoethyl)-tyrosine (O2beY) using an engineered *Mj*TyrRS/tRNA_CUA_ pair and a proximal cysteine thiol group^360,364^ (Figure 16a), and they combined this technique with a low-throughput, plate-based assay to develop a macrocyclic peptide inhibitor targeting the Sonic Hedgehog/Patched 1 interaction.^365^ Based on these established techniques, the same team^366^ further constructed a nonreducible thioether-cyclized phage display library, termed as MOrPH phage display system (MOrPH-PhD), a high-throughput and versatile platform for the discovery and evolution of functional macrocyclic peptides tailored for targeting proteins and protein-mediated interactions. In this system, the same cysteine-reactive O2beY was genetically encoded to achieve spontaneous, posttranslational cyclization of peptides combined with M13 bacteriophage display technology, facilitating the generation of genetically encoded macrocyclic peptide libraries displayed on the surface of phage particles. This cyclization technique was successfully used to construct various combinatorial libraries and yielded high-affinity binding peptides for three target molecules: streptavidin, Kelchlike ECH-associated protein 1, and Sonic Hedgehog.

Other research teams also have employed different variants of *Mj*TyrRS/tRNA_CUA_ pair for the precise incorporation of ncAAs into phage-displayed proteins, which can be labeled through azide–alkyne cycloadditions^367,368^ and Staudinger ligations.^369^ However, different variants of the *Mj*TyrRS/tRNA_CUA_ pair can only incorporate phenylalanine-derived ncAAs but cannot incorporate diverse aliphatic ncAAs, while PylRS/tRNA pair can incorporate diverse aliphatic ncAAs into proteins.^370^ Wang et al.^371^ developed a genetically encoded phage display cyclic-peptide library that takes advantage of PylRS/tRNA. The displayed peptides are cyclized through a proximity-driven Michael addition reaction between a proximal cysteine residue and an electrophilic aliphatic ncAA *N*^ε^-acryloyl-lysine (AcrK) encoded by an evolved PylRS variant (PrKRS) and its cognate tRNA_CUA_.^372^ Using a random 6-mer peptide library where peptides are spontaneously cyclized at both ends through cysteine-AcrK linkage (Figure 16a), they demonstrated the successful selection of effective ligands for tobacco etch virus (TEV) protease and HDAC8. Additionally, most epigenetic regulators operate through a binary mechanism with both active sites and peptide-binding grooves to identify their substrates, but inhibitors designed for these regulators primarily focus on their active sites, resulting in significant issues with low selectivity. Tharp et al.^342^ developed an amber-obligate phage library to display peptides, which were genetically incorporated by a ligand-fused ncAA *N*^ε^-butyryl-l-lysine (BuK) through an evolved PylRS variant (BuKRS) and its cognate tRNA_CUA_,^372^ for rapid selection of ligands binding to aepigenetic regulator, NAD-dependent sirtuin SIRT2. The phage library was created with degenerate primers incorporating NNK triplet nucleotide sequences, which randomly encoded only one amber codon and other sense codes instead of the amber codon only at fixed position^373^ in peptide-coding sequences, followed by a superinfection-immunity-based selection to isolate clones containing an amber codon, effectively excluding those with solely sense codons or harmful mutations from the library. The selected peptides, which were substituted with another ligand-fused thiobutyryl analogues (*N*^ε^-thiobutyryl-l-lysine, tBuK) and thiomyristoyl (*N*^ε^-thiomyristoyl-l-lysine, tMyK) in place of BuK, displayed selective inhibition against other sirtuin isoforms and showed more effective than previously reported small-molecule sirtuin inhibitors.^374^ In general, the expression of phage libraries containing ncAAs depends on a three-plasmid expression system including one plasmid encoding randomized peptide library, one plasmid encoding the RS/tRNA pair essential for amber suppression using a ncAA and another helper phage plasmid which supplies the necessary phage proteins for packaging. Nonetheless, this three-plasmid system used for amber-obligate phage display encounters challenges such as low library transformation efficiencies and the prerequisite for prior amber enrichment before phage expression (Figure 16b). Recently, by integrating a novel PylRS/PylT pair, CMaPylRS/PylT, which is found in *Candidatus Methanomethylophilus alvus* and much smaller (825 bp versus 1200–1300 bp), into the CM13d3 helper phage to generate a novel helper phage, CMa13ile40, Hampton et al.^375^ further utilized this novel two-plasmid system (Figure 16b) for continuous enrichment of amber-obligate phage clones for two different libraries, demonstrating a remarkable increase in packaging selectivity compared to the original CM13d3 helper phage. The CMa13ile40 was subsequently employed to generate two peptide libraries, each containing distinct ncAAs, BocK and AlocK, for identification of peptide ligands bound to the extracellular domain of ZNRF3, which is a membrane-bound E3 ubiquitin ligase known for its role in developing proteolysis-targeted chimeras (PROTACs) aimed at degrading oncoproteins.^376^ Notably, each selection process exhibited distinct enrichment patterns of unique sequences based on the specific ncAA employed, and the peptides from both selections were confirmed to exhibit low micromolar affinity for ZNRF3, contingent upon the presence of the ncAA used during the selection process.

Additionally, phage display can be also used for selection of larger protein drugs, such as antibodies, taking advantage of covalently cross-link reactivity. The main approaches for creating covalent protein adducts involve utilizing either photo-cross-linkable or spontaneously cross-linkable functional groups. Chen et al.^377^ devised a method for the straightforward selection and recognition of antibodies that specifically bind to a particular epitope of an antigen, which was site-specifically incorporated by BpA with photo-cross-linking reactivity through GCE.^19,91,378^ The modified antigen was then employed in the epitope-directed panning process against antibody phagemid display libraries from B cell repertoires of different sources. Based on the Bpa-incorporating epitope to form a covalent bond with antibody-displayed phages in proximity under UV irradiation, only the phages that bind to the target epitope are enriched and selected during stringent washing steps. This approach was employed for identified hits targeting specific epitopes within human interleukin-1β (IL-1β) and complement 5a (hC5a), both yielding more than one-third of the identified hits bound to target epitopes. On the other hand, Alcala-Torano et al.^379^ utilized yeast display combined with ncAAs to identify irreversible variants of single-domain antibodies (sdAbs) that target botulinum neurotoxin light chain A (LC/A). Based on a series of structurally characterized sdAbs, they assessed antibodies substituted with reactive ncAAs, AzF (for photo-cross-linking) and OBeY (for spontaneous cross-linking) combined with systematic evaluations in yeast display format of over 40 ncAA-substituted variants, multiple clones that retained binding function were discovered while gaining UV-mediated or spontaneous cross-linking abilities. Solution-based analyses revealed that ncAA-substituted clones exhibit site-dependent target specificity and cross-linking abilities uniquely provided by ncAAs, emphasizing the potential of yeast display combined with GCE in discovering binding agents that covalently engage their targets. In brief, these platforms simplify the discovery and characterization of antibodies with therapeutically relevant properties that are not accessible through the canonical genetic code. It is worth mentioning that introducing ncAAs containing covalent reactive group to the target protein could also facilitate fragment-based drug discovery through bioorthogonal coupling reactions. For example, Mattheisen et al.^380^ screened conformational modulators for G protein-coupled receptor (GPCR) C–C chemokine receptor 5 (CCR5) by incorporating cyclopropene-l-lysine (CpK) adjacent to a conformationally sensitive binding site modulated by allosteric antagonist maraviroc (mvc) in CCR5. The chosen CpK harboring a cyclopropene handle is known for its capacity to undergo bioorthogonal IEDDA coupling reactions with tetrazine-containing compounds.^381^ A series of heterobifunctional mvc analogues, which featured an mvc pharmacophore, a variable-length polyethylene glycol (PEG) linker, and a reactive tetrazine or methyltetrazine group, were developed and the pharmacological activities of these various mvc analogues were characterized. Contrasting with conventional biophysical fragment screening methods, this novel approach eliminates the necessity for extensive protein purification. The entire process, including receptor expression, orthogonal conjugation of drug fragments, and pharmacological evaluation, can be efficiently performed on individual cells in microtiter plate wells. This methodology not only streamlines the process of fragment-based drug discovery using live cells but also potentially enhances the ability to investigate state-dependent and dynamic cryptic sites on GPCRs.

#### 4.2.6 Synthesis of Cyclic Peptide/Peptide-Like Products

Macrocyclization can mitigate certain drawbacks of peptides^357,365,382^ and impart unstructured peptides to gain conformational rigidity, frequently enhancing their ability to bind to specific targets.^383^ Additionally, cyclic peptides exhibit much higher resistance to proteolytic degradation.^384^ GCE technology has been widely used to biosynthesize of cyclic peptides and peptide-like products which exhibit potent antibacterial, antifungal, and antiviral activities, by introducing novel chemical structures into these biologically active compounds.^385^ For instance, Zambaldo et al.^386^ altered the native macrocyclic structure of lanthipeptide (Nisin A) by introducing ncAAs at discrete positions, including a ncAA containing α-chloroacetamide group, which enabled the production of Nisin variants with innovative macrocyclic structures. Tai et al.^387^ presented a scalable and robust system that relies on the natural synthesis of a ncAA d-Cys-ε-Lys, integrated with optimized PylRS/tRNA_CUA_ pair and specific cell lines, facilitating the creation of lasso-grafted proteins. They applied this system to cyclize a 23-amino acid therapeutic P16 peptide, grafted onto an MBP tag via intein-mediated native chemical ligation (NCL)^388^ between the d-Cys-ε-Lys and C-terminal thioester, and the resulting cyclic P16 peptide fusion protein exhibited significantly higher binding affinity for CDK4 compared to its linear counterpart. They further generated a bifunctional bicyclic protein featuring a cyclic cancer cell-targeting RGD motif on one end and the cyclic P16 peptide on the other, demonstrating potent cell cycle arrest properties and improved serum stability. Additionally, different research teams also successfully applied ncAAs via GCE to thiopeptides,^389^ cinnamycin,^390^ lanthipeptides,^391,392^ lasso peptides,^363,393,394^ and other post-translationally modified peptides.^391^ Moreover, the demand for peptide-based pharmaceuticals has been steadily rising, Zhang et al.^395^ and Kelemen et al.^396^ successfully leveraged GCE-based incorporation of azide-bearing ncAAs into a cyclic-RGD peptide (cRGD) and bioorthogonal conjugation strategies to selectively attach cRGD onto the Adeno-Associated Virus (AAV) capsid protein to enhance the specificity of AAV targeting the integrin receptor. Recently, considering that the lack of orthogonal PylRS/tRNA that accept non-l-α-amino acids is a major obstacle hindering the in vivo translation of sequence-defined hetero-oligomers and biomaterials, Fricke et al.^397^ demonstrated that pyrrolysyl-tRNA synthetase (PylRS) and specific PylRS variants can accept α-hydroxy, α-thio, and *N*-formyl-l-α-amino acids, as well as α-carboxy acid monomers, which are precursors to polyketide natural products. These monomers are accommodated and accepted by the translation machinery in the in vitro translation reactions, and those with reactive nucleophiles are incorporated into proteins expressed in wild-type or engineered *E. coli* strains, highlighting the promise of PylRS as a platform for engineering novel enzymes that could work in concert with natural or engineered ribosomes to produce a wide range of sequence-specific nonprotein heteropolymers.

Despite remarkable progresses, however, these efforts to create noncanonical macrocycles have been confined to incorporating just a single type of ncAA with modest efficiency in response to amber stop codon. In recent years, total synthesis of *E. coli* with a recoded genome in combination with GCE has enabled the efficient synthesis of different cyclic peptide/peptide-like products. Leveraging evolution of the genetically recoded Syn61,^126^ Jason Chin and his colleagues^127^ further obtained Syn61Δ3 and its evolved derivatives to facilitate the cellular synthesis of polymers composed entirely of ncAAs and a single five-membered macrocycle containing four ncAAs. Moreover, the same team^128^ also orchestrated synthesis of 25 varied non-natural macrocyclic peptides, each incorporating two ncAAs in Syn61Δ3-derived cells. To create genetically programmed cell-based synthesis of non-natural depsipeptide macrocycles (or any non-α-l-amino acid linked macrocycle), they further utilized PylRS/tRNA pairs from different classes to facilitate the cotranslational integration of various α-hydroxy acids encompassing both aliphatic and aromatic side chains, and finally identified a total of 49 engineered, mutually orthogonal pairs capable of recognizing distinct ncAAs or alpha hydroxy acids and deciphering distinct codons. Ultimately, they combined these progresses to program Syn61Δ3-derived cells for the encoded synthesis of 12 diverse non-natural depsipeptide macrocycles, featuring two noncanonical side chains and either one or two ester bonds. By altering the gene sequence, they strategically positioned noncanonical monomers (ncMs) at various points in the macrocycles. Furthermore, by modifying the reassignment scheme, they were also able to vary the monomers encoded at each position.^128^ To further expand the chemical diversity of the genetic code to include new classes of monomers, recently Jason Chin’s lab^160^ has introduced a novel technique tRNA display to identify orthogonal synthetases that are capable of acylating their corresponding orthogonal tRNA with a range of eight ncAAs and eight different ncMs, including a variety of β-amino acids, α,α-disubstituted-amino acids, and β-hydroxy acids, thereby facilitating the genetic encoding β-amino acids and α,α-disubstituted amino acids into proteins and the encoded synthesis of noncanonical polymers and peptide-like products.

#### 4.2.7 Vaccine Development

Prophylactic vaccines are designed to provide long-term protection against pathogens by activating the adaptive immune system. Therapeutic vaccines express specific antigens to induce cell-mediated immunity by activating cytotoxic T cells or humoral immunity by activating B cells to produce specific antibodies.^398^ These vaccines could benefit from GCE by adding unnatural building blocks.^7^

**Figure 17:**
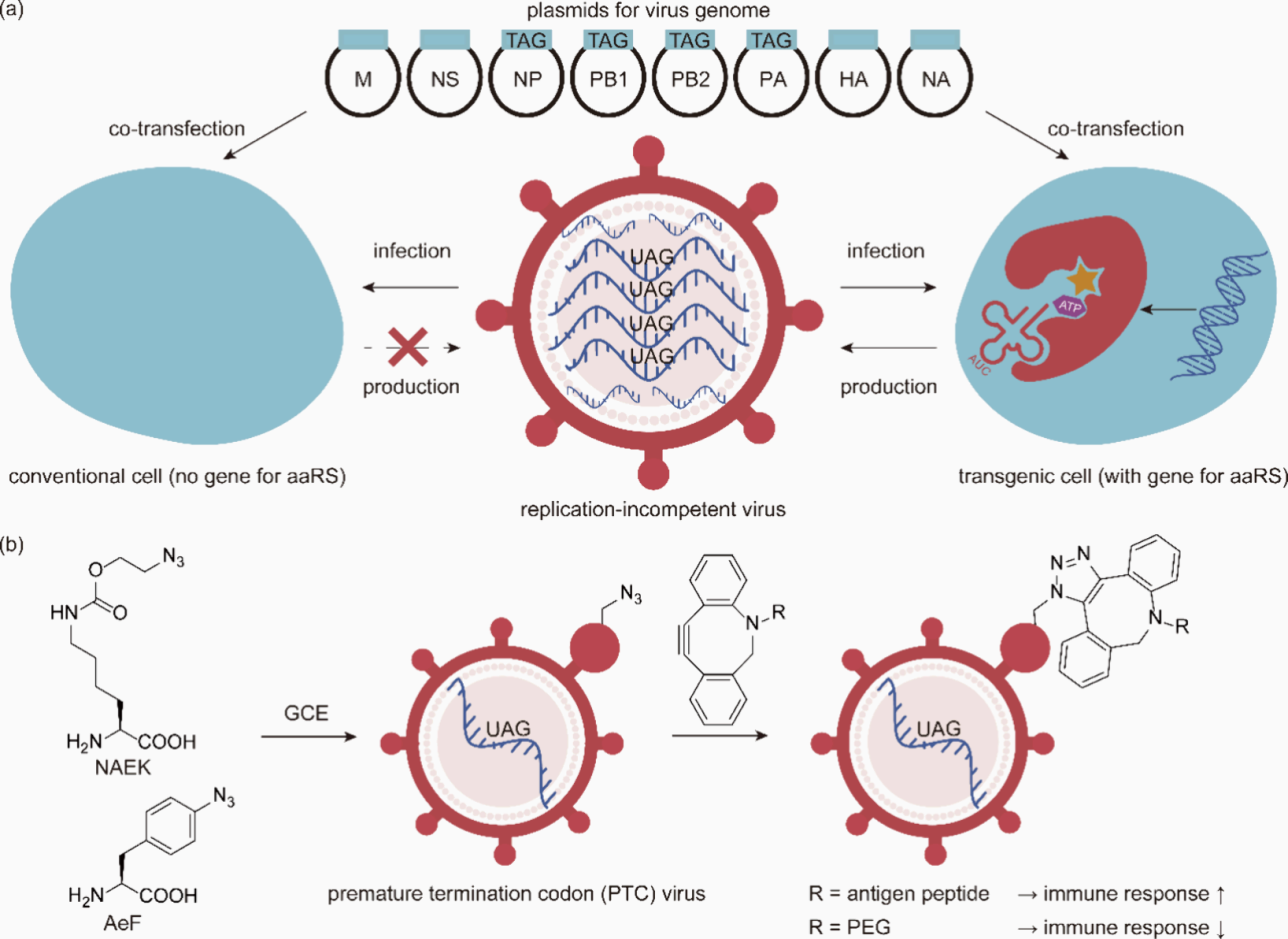
Vaccine development. (a) Schematic illustration of the creation of premature termination codon (PTC) influenza viruses. These viruses exhibit replication incompetence in standard cells but exhibit high replication in transgenic cells. The transgenic cells contain integrated cassettes for the expression of orthogonal tRNA (tRNA_CUA_) and tRNA synthase (pylRS). NP, nucleoprotein; PB1 and PB2, polymerase basic proteins 1 and 2; PA, polymerase acidic protein; M, matrix protein; NS, nonstructural protein; HA, hemagglutinin; NA, neuraminidase. (b) Modification for multifunctional virus vaccines. The ncAAs on virus serve as bioorthogonal reaction handles for conjugation with components like antigen peptide or PEG to modify virus and change the functions of virus.

One major application of GCE is creating live but replication-incompetent viruses. By introducing premature termination codons (PTCs) into essential viral proteins, researchers can produce viruses that are unable to replicate in normal cells but can still stimulate the immune system. This approach has been successfully used in the development of vaccines for HIV-1 and influenza A, providing both safety and efficacy (Figure 17a).^198,399,400^ For instance, Si et al. used this method to create an influenza A virus that can only replicate in cells containing specific ncAAs, thus eliminating the risk associated with live virus vaccines.^400^ This innovative approach offers excellent new possibilities for vaccine production.

Additionally, GCE enables the modification of viruses with additional functional elements (Figure 17b). Ji et al. incorporated NAEK into influenza A virus (WSN) to conjugate antigenic peptides to the virus surface.^401^ This engineered virus resulted in strong antigen uptake by dendritic cells, specific immune cell responses, and a significant increase in tumor-infiltrating lymphocytes. This vaccine successfully induced strong immune responses and transformed a “cold” tumor microenvironment into a “hot” one.

Moreover, GCE allows the development of multivalent nanoscaffolds for presenting antigens. Castro et al. used ncAAs to conjugate polyethylene glycol (PEG) to the domain III protein of the Zika virus envelope, refocusing the immune response toward protective antibody targets.^402^ They also created multivalent nanoscaffolds for vaccine applications using ncAAs.

#### 4.2.8 Drug-Delivery Systems

Drug delivery systems are essential in disease treatment and prevention, offering a platform for precise therapeutic delivery to targeted sites.^403^ These systems prioritize adjustable release, precise targeting, prolonged circulation, and safety and efficacy of drugs. Protein-based polymers stand out for their adaptability and biological compatibility, making them ideal for drug delivery applications.^404^ The integration of ncAAs through protein engineering enhances these systems, enabling precise delivery and multifunctionality.^405^

A series of protein-base polymers has been utilized in drug delivery system, such as elastin-like polypeptides, resilin-like proteins, and silk-like proteins. These polymers, composed of representative repeat sequence, usually have distinctive properties. One of the most common used polymers is elastin-like polymers which have the ability of temperature responsive phase transition, therefore making it easy to purify and achieving temperature responsive drug release.^406^ The incorporation of ncAAs addresses the unmet need for fine-tuning the assembly behavior of proteins and endow the protein polymers with novel physicochemical properties and biological functions. Incorporation photoswitchable azobenzene containing amino acids have effect on the transition temperature of elastin-like polymers and hold promise in different temperature response delivery strategy.^407^ More recently, incorporation of 15 different aromatic ncAAs into elastin-like proteins has shown to have effect on both phase transition temperature and secondary structure of proteins.^408^ The transition temperature of elastin-like proteins spanned about 60 °C. And the same conclusion was also elucidated in resilin-like proteins, another type of temperature responsive polymers. The phase transition temperature of resilin-like proteins ranged from 38.3 to 63.9 °C. Moreover, AzF was incorporated into resilin-like proteins and then UV irradiation was adopted to realize irreversible intra- and intermolecular covalently cross-linking. The cross-linking plays a crucial role in the stability of resilin-/elastin-like diblock polypeptides and holds promising potential in the construction of the drug-loaded core-cross-linked nanoparticles.^409^

Through the ncAAs incorporation, the desired ligands or molecules can also be attached to the surface of proteins to amplify the performance of drug delivery. In this way, the AzF was cotranslationally incorporated in the amphiphilic elastin-like proteins library and small organic dyes were attached via SPACC reaction.^410^ The presence of small organic dyes showed diverse supramolecular protein assemblies clearly, such as spherical coacervates, twisted fiber bundles and vesicles. The protein assemblies have the capability of encapsulation of molecules with different size and physicochemical properties which paved the road for targeted drug delivery. In another study, Simone et al. presented a universal platform to accomplish site-specific drug conjugation and efficient targeting.^411^ The pAF containing elastin-like polypepetides and nanobody are fused to express, and then pAF is utilized as a biorthogonal handle for drug attachment. Besides, more functions may be achieved when combine the click chemistry with other chemical and biological cross-linking methods. In one study, via SPACC reaction based on AzF and cysteine-maleimide chemistry, Cas9 protein was conjugated with drug and carrier polymers and provided a multifunctional platform for delivery, gene editing, and therapy.^412^

#### 4.2.9 Gene Editing

Gene editing technology has emerged as a groundbreaking scientific advancement that promises to reshape the landscape of genetics and biotechnology. Especially the recently emerged Clustered Regularly Interspaced Short Palindromic Repeats (CRISPR)/Cas9, which has won the Nobel Prize in Chemistry in 2020, surpassing other gene editing methods in terms of ease of use. GCE technology has also played an important role in the optimization of gene editing tools. Beyond harnessing the reactivity inherent in ncAAs for the modulation of CRISPR/Cas9 activity,^413^ GCE technology also enhanced the efficacy and versatility of gene editing. By incorporating a genetically encoded 4-(2-azidoethoxy)-l-phenylalanine (AeF) into recombinant Cas9 protein, Ling et al.^414^ established a methodology for the precise conjugation of oligonucleotides, resulting in local concentration of donor DNA increasement and a significant enhancement of the homology-directed repair (HDR) efficiency in both human cell cultures and mouse zygotes.

The same method was applied not only to donor DNA but also to site-directed coupling of Cas9 and crRNA,^415^ further shortening the customized RNA scaffolds. Beyond Cas9, crRNA has also been conjugated to Cas12a,^416^ a candidate with a substantially lower incidence of off-target effects compared to Cas9. However, owing to its reduced binding affinity for crRNA, it has been suffering from comparatively lower genome editing efficiency. The resulting conjugated Cas12a complex (cCas12a) exhibited significantly improved genome editing efficiency when compared to its wild-type counterpart.

Moreover, the GCE technology has made substantial contributions to the field of CRISPR-based drug delivery. In a study conducted by Beha et al.,^412^ CRISPR/Cas9 was adeptly conjugated with the chemotherapeutic compound olaparib and a carrier polymer (CP). This strategic combination resulted in the development of a multifunctional self-delivered nanomedicine platform designed for the precise targeting of the RAD52 gene, ultimately yielding outstanding antitumor outcomes. This novel platform was aptly termed the “combinatorial and bioorthogonal nano-editing complex” (ComBiNE).

### 4.3 Mechanistic Studies

#### 4.3.1 Studies of Biological Mechanisms

The advancements in GCE technology, which allows for the site-specific incorporation of ncAAs into proteins, enabling precise modifications of proteins to study various physiological mechanisms, post-translational modifications (PTMs), protein–protein interactions, and dynamic cellular processes. The basic application of GCE is used to introduce ncAAs, regardless of its functional groups, into specific proteins, enabling the controlled translation and expression of full-length proteins, which has been applied to study circadian rhythms, particularly in the suprachiasmatic nucleus (SCN), by manipulating the expression of CRY1 protein in neurons and astrocytes.^417−420^ Besides the translational switching to directly regulate gene expression without considering functional groups of ncAAs, almost all GCE applications is strictly dependent on versatile groups of ncAAs, such as mimicking PTMs such as phosphorylation, acetylation, and ubiquitination, cross-linking to stabilize transient protein complexes, and incorporating fluorescent probes for tracking protein dynamics. Specifically, GCE allows the incorporation of ncAAs that mimic natural PTMs like phosphorylation, acetylation, methylation, and ubiquitination, etc. This enables the study of complex PTMs, which are difficult to handle using traditional methods, and provides insights into their roles in cellular processes. Covalent ncAAs serve as proximity probes to capture interactions between proteins and other molecules. Techniques like photo-cross-linking and chemical cross-linking stabilize protein complexes, even those with weak or transient interactions, for detailed analysis. Genetically encoded fluorescent amino acids or ncAAs with reaction handles are used for precise imaging and tracking of proteins in situ, which facilitates the study of dynamic physiological processes and protein interactions using methods like FRET and super-resolution imaging. GCE enables site-specific labeling for NMR studies, allowing the exploration of protein structure and dynamics, particularly for large transmembrane protein complexes. Techniques like ^19^F NMR and ^1^H NMR provide insights into conformational changes and interactions within proteins. NcAAs incorporated at key sites in ion channels can alter their conformations and functions, helping researchers understand the roles of specific residues in voltage changes and ion channel behavior. These innovations highlight the versatility and broad applicability of GCE technology in advancing our understanding of various biological mechanisms through precise molecular manipulations.

##### 4.3.1.1 Studying Protein Post-Translational Modification (PTM)

**Figure 18:**
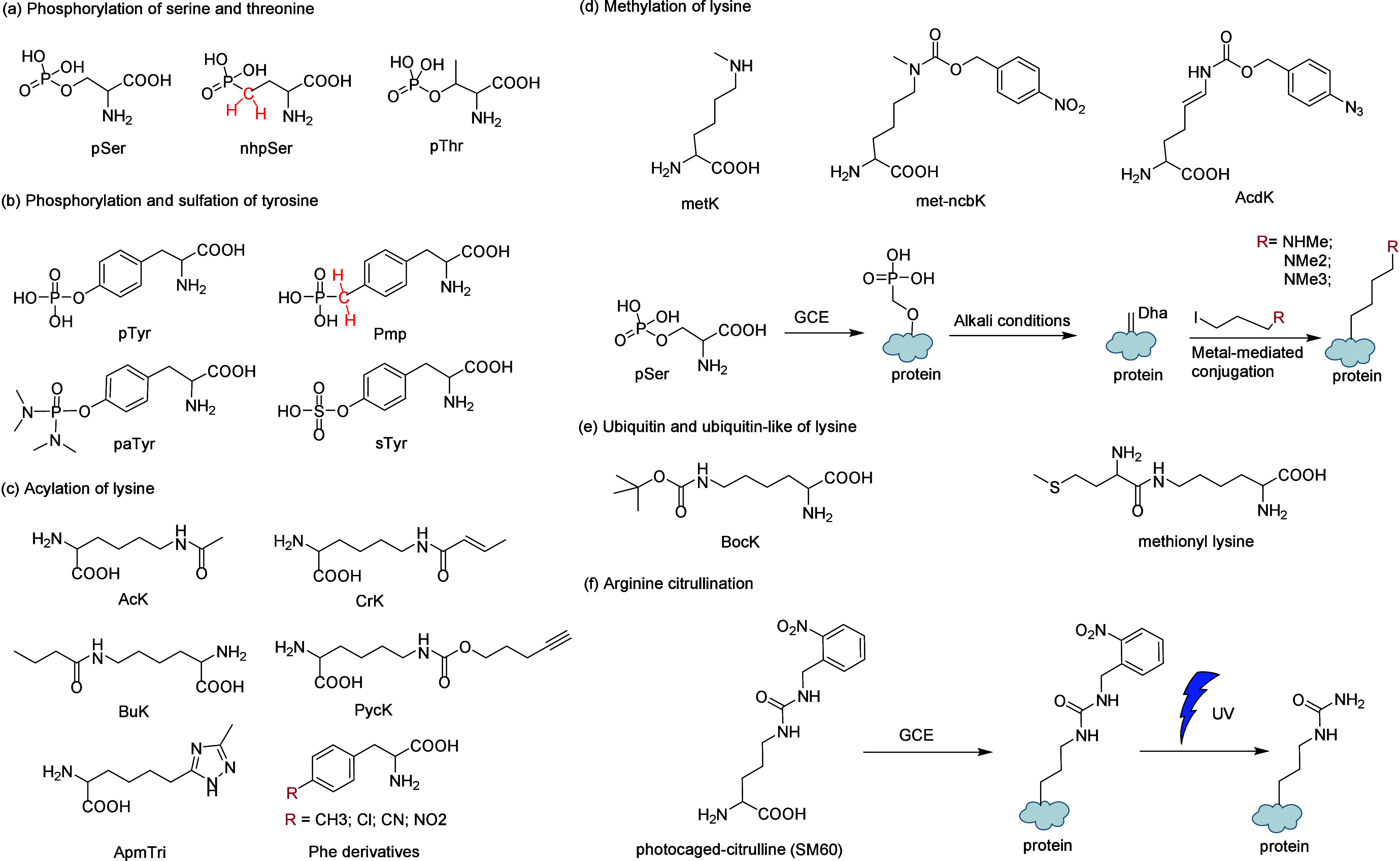
NcAAs used for exploring biological mechanisms of serine/threonine phosphorylation, tyrosine phosphorylation/sulfation, lysine acylation/methylation/ubiquitin, and arginine citrullination, via GCE.

Given the complexity of PTMs, current methods for obtaining proteins with PTMs, such as using amino acid mutation to mimic modified groups, enzyme-catalyzed reactions, chemical synthesis, etc., are challenging to accurately replicate the in vivo activity of PTMs. And the purity and yield of modified products are low, making it difficult to further apply them for studying PTMs in cells. GCE technology introduces orthogonal aaRS/tRNA pairs, which specifically recognize and incorporate ncAAs at specific sites in proteins, resulting in proteins with PTMs (Figure 18). The genetically encoded ncAAs such as phosphorylated serine/threonine/tyrosine and acetylated lysine, are natural PTM groups, avoiding the artificial and nonphysiological results caused by simple amino acid mutation to mimic PTMs.

###### 4.3.1.1.1 Phosphorylation of Serine, Threonine, and Tyrosine

(a) *Phosphorylation of Serine*. Post-translational phosphorylation of Ser, Thr or Tyr residues in proteins plays a crucial role in various human cellular processes,^421,422^ yet its study in native systems is hindered by its transient and heterogeneous nature. Until now, site-specific incorporation of phosphoserine (pSer) can be genetically encoded in mammalian cells in contrast to inabilities of phosphothreonine (pThr)/phosphotyrosine (pTyr) to be incorporated in mammalian cells, while GCE approaches have provided tremendous opportunities to incorporate them into recombinant proteins in *E. coli*. pSer represents the most prevalent phosphor-amino acid in the eukaryotic phosphoproteome.^423^ Park et al.^108^ pioneered the genetical encoded pSer in human mitogen-activated ERK activating kinase 1 (MEK1) by engineering an *E. coli* strain that harbors a *M. maripaludis*-derived pSer–tRNA synthetase (SepRS), its cognate *M. jannaschii*-derived Sep-accepting tRNA (tRNA^Sep^), and an engineered EF-Tu (EF-Sep). Liu et al.^424^ further employed this evolved SepRS/tRNA_CUA_^108^ to incorporate pSer at the position 361 of Rpn1 expressed in an engineered *E. coli* C321^425^ and revealed that phosphorylation of Rpn1 at Ser361 enhances proteasome assembly by facilitating its interaction with Rpt2, a critical step in the 19S regulatory particle formation. Moreover, to enhance both efficiency and accuracy for incorporating pSer, Rogerson et al.^45^ developed novel SepRS/tRNA_CUA_ pairs that facilitate the efficient incorporation of pSer and nonhydrolyzable pSer analogue (nhpSer) into recombinant proteins in a metabolically engineered *E. coli* strain without requirement of mutations in EF-Tu, enabling to purify several milligrams-per-liter of proteins with biologically significant phosphorylations that were previously difficult or impossible to obtain. As examples, the phosphorylated ubiquitin at serine 65 and the kinase Nek7 at serine 195, which are artificially activated by genetically encoded pSer, can respectively activate Parkin E3 ligase activity, and robustly phosphorylate myelin basic protein (MBP) as well as bypass activation by the C-terminal domain of Nek9 (Nek9-CTD) in vitro. Since then, this efficient SepRS/tRNA_CUA_ pair^45^ has been widely used to incorporate pSer into proteins both in *E. coli* and in mammalian cells for study of different biological mechanisms. For example, Huguenin-Dezot et al.^426^ generated substantial quantities of phosphorylated ubiquitin at different serine sites expressed in BL21 *ΔserB*(DE3) cells^45^ and found that phosphorylation at these sites can either activate or inhibit the cleavage of particular ubiquitin linkages by deubiquitinases (DUBs), highlighting the critical role of phosphorylation in regulating ubiquitin signaling pathways. Similarly, Dickson et al.^427^ also incorporated pSer into Fc receptor TRIM21, demonstrating that TRIM21’s signaling is inherently repressed by its B-Box domain and is activated via phosphorylation. This phosphorylation facilitates TRIM21’s ability to bind to ubiquitin enzymes, thereby enhancing its immune signaling functions. In addition, by generating a phosphorylated chimeric protein (ClACCp) containing clathrin heavy chain (CHC) (aa1–574) and CHC interaction domain (TACC3^CID^, aa549–570) of TACC3 (a member of the transforming acidic coiled-coil family of proteins separated by four repeats of Thr-Gly-Ser linkers (TGS4) with pSer incorporation of at TACC3 residue 558 (ClACCp) using the same SepRS/tRNA,^45^ Burgess et al.^428^ explored how Aurora-A kinase regulates the recruitment of TACC3 to the mitotic spindle through phosphorylation, thereby showing that phosphorylation of Ser558 and a hydrophobic docking motif in TACC3 play key roles in engaging with Aurora-A and CHC, highlighting a mechanism that could be common in phosphorylation-dependent signaling pathways. On the other hand, Beránek et al.^429^ introduced SepRS/tRNA_CUA_^45^ into mammalian cells, combined with creation of an eukaryotic elongation factor variant EF-1α-Sep and a release factor eRF1(E55D) mutant and manipulations of intracellular phosphorylated serine biosynthesis pathway to enhance incorporation efficiency of pSer and nhpSer incorporation. Then nhpSer was successfully inserted into the protein kinase MEK1 at position 218, confirming that phosphorylation of serine can fully activate MEK1 and ERK phosphorylation.

Furthermore, the application of GCE technology for pSer incorporation was not just confined to the examination of single or several proteins, it has also provided a proteome-level insights into the overarching significance of phosphorylation in complex systems with the absence of phosphoproteome-level biological methods. Barber et al.^430^ devised a proteome-wide approach to investigate phosphorylation and its involvement in protein–protein interactions. By genetically incorporating pSer in *E. coli* C321.ΔA,^106,113^ they created a peptide-based synthetic human serine phosphoproteome (SHSP) with precise verification of pSer incorporation in more than 36 000 of these peptides at specific sites, based on a single-plasmid library containing 110 139 human phosphopeptides. Through the application of a library screening technique for identify which pSer-containing phosphopeptide library members interact with a phosphorylation binding domain of interest, they successfully uncovered numerous known and possible new interactions involving pSer with 14–3–3 proteins and WW domains. Importantly, these identified phosphosites exhibited critical binding characteristics akin to the native human phosphoproteome, as confirmed through motif analysis and pull-down assays involving full-length phosphoproteins. Similarly, Gassaway et al.^431^ developed another new version of pSer-containing SHSP termed as Iterative Synthetically Phosphorylated Isomers (iSPI) to achieve multipurpose application. They restructured the SHSP into ten distinct subpools, each meticulously crafted to segregate phosphopeptide positional isomers into separate pools so that the phosphorylation positional isomers are unique. By using iSPI and the latest pSer orthogonal translation system (OTS) (pSerOTS),^432^ approximately 500 μg-per-liter of phosphopeptides can be produced in *E. coli* cultures, containing approximately 11 000 unique phosphopeptides with known phosphorylation sites in each subpool. This SHSP library stands out for being cost-effective, renewable, and diverse, with accurately identified phosphorylation sites.

Nonetheless, generating homogeneous proteins containing pSer in mammalian cells remains a difficult task because of the numerous endogenous phosphatases present. Although *E. coli* lacks the complex phospho-signaling systems found in eukaryotes, phospho-proteins generated through GCE can undergo dephosphorylation during protein expression,^180,433,434^ which even hinders subsequent in vitro characterization in some scenarios, then the capability to incorporate a nonhydrolyzable pSer mimic (nhpSer) is an essential alternative to directly installing pSer. Recently, by engineering a six-step biosynthetic pathway that produces nhpSer from phosphoenolpyruvate and introducing the previous SepRS/tRNA^45^ in *E. coli*, Zhu et al.^435^ developed a “PermaPhos” system for the autonomous biosynthesis of nhpSer-containing proteins, which overcomed previous limitations in synthesizing phosphorylated proteins, allowing for a 40-fold improvement of nhpSer-containing protein production. By enabling the site-specific incorporation of nhpSer into three biologically relevant proteins, they demonstrated the potential of “PermaPhos” system in elucidating phosphorylation-dependent protein interactions and signaling pathways, particularly with the 14–3–3ζ protein, highlighting its applicability in both basic and applied research contexts.

(b) *Phosphorylation of Threonine*. Phosphorylation of threonine residues in proteins plays a pivotal role in regulating various processes including energy metabolism, apoptosis, cell cycle, and signal transduction pathways within eukaryotic cells.^436−439^ To advance understanding of how threonine phosphorylation influences biological functions, the requirement of general methods for synthesizing well-defined phospho-proteins was urgent. Zhang et al.^180^ employed a novel approach that combines parallel positive selections with deep sequencing, resulting in screening out a phosphothreonyl-tRNA synthetase/tRNA_CUA_ pair (pThrRS/tRNA^v2.0^_CUA_) based on the previous SepRS/tRNA.^45^ Due to the poor transmembrane transport capacity of pThr, they built a pThr biosynthesis pathway in *E. coli* cells to increase intracellular pThr concentration, thereby enhancing pThr incorporation into cyclin-dependent kinase 2 (Cdk2) at position 160 via pThrRS/tRNA^v2.0^_CUA_. Cdk2 (pThr160) exhibited significantly higher activity than the wild-type Cdk2 in phosphorylating histone H1, thereby showcasing synthetic kinase activation achieved through the encoding of pThr in the activation loop. Moen et al.^440^ further engineered the pThrRS/tRNA^v2.0^_CUA_ pair and obtained a more finely tuned OTS capable of efficiently incorporating pThr, followed by strain optimization that maximized phosphoprotein production, facilitating the highest pThr-containing protein production. Using this system, they elucidated the activation mechanism that phosphorylation of checkpoint kinase 2 (CHK2) activation loop at Thr383 is essential and sufficient to activate kinase activity and profiled the overlap between CHK2 substrate phosphorylation and 14–3–3β phosphorylation-dependent interactions.

(c) *Phosphorylation of Tyrosine*. Compared to serine/threonine phosphorylation, the distribution of tyrosine phosphorylation is much less in mammalian cells.^423^ To introduce tyrosine phosphorylation modifications using GCE technology, Fan et al.^111^ conducted in vitro screening of 76 *Mj*TyrRS mutants and identified two mutants, named pYRS1 and pYRS2, which exhibited enhanced recognition of pTyr. In a subsequent experiment, pYRS1, tRNA^Tyr^_CUA_, and EF-Tu mutants were coexpressed in a distinct *E. coli* strain lacking five phosphatase-encoding genes to stabilize pTyr, and a substantial amount of exogenous feeding pTyr (10 mM) was needed to facilitate the insertion of pTyr into proteins. Like pSer and pThr, pTyr also carries a phosphate group with negative charge, resulting in poor permeability to *E. coli* cell wall, thus reducing efficiency pTyr incorporation via GCE. To address this issue, two research groups independently made modifications to pTyr to enhance cellular uptake. Luo et al.^441^ leveraged the ability of *E. coli* dipeptide transporter DppA to recognize N-terminus of all 20 cAAs without restriction toward the C-terminus residue. Thus, two synthesized dipeptides (Lys-pTyr and Lys-Pmp) can be easily transported into *E. coli* cells and then hydrolyzed by intracellular nonspecific peptidases, yielding free pTyr or the nonhydrolyzed pTyr analogue 4-phosphomethyl-phenylalanine (Pmp). Using the evolved *Mj*TyrRS variant CMFRS and *Mj*tRNA^Tyr^, pTyr and Pmp were successfully incorporated into the target proteins. By incorporating Pmp into a putative phosphorylation site of Abelson murine leukemia viral oncogene homologue 1 (ABL1),^442^ they assessed the affinities of the various ABL1 variants containing Pmp to the substrate 3BP2 peptide.^443^ On the other hand, Hoppmann et al.^444^ designed an electrically neutral and stable pTyr analogue termed as phosphoramidate tyrosine (paTyr), which was incorporated into the proteins using an evolved *Mm*PylRS-derived mutant *Mm*NpYRS. The analogue containing a phosphoramidate group can be cleaved under acidic conditions to convert into a native pTyr,^445^ ultimately yielding pTyr-containing proteins. Using this approach, they explored the impact of tyrosine phosphorylation on the structure and function of ubiquitin, uncovering a potential inhibitory role of ubiquitin phosphorylation.

###### 4.3.1.1.2 Acylation of Lysine

Protein lysine acylations encompass a diverse range of protein modifications, characterized by variations in acyl-group length, charge, and saturation, and they are closely associated with numerous vital physiological processes. Lysine acylations include lysine acetylation (Kac), crotonylation (Kcr), butyrylation (Kbu), propionylation (Kpr), malonylation (Kmal), glutarylation (Kglu), benzoylation (Kbz), 2-hydroxyisobutyrylation (Khib), β-hydroxybutyrylation (Kbhb), succinylation (Ksucc), and lactylation (Kla).^446^ Lysine acetylation (Kac) represents a reversible posttranslational modification of proteins which refers to an alteration where an acetyl group is transferred to the ε-amino group of lysine residues. Notably, acetylation of lysine eliminates the positive charge of the side chain, thereby directly influencing the electrostatic properties of the modified protein.^447^ Engineered PylRS variants can recognized *N*^ε^-acetyl-lysine (AcK) and nonhydrolyzable analogues such as thioacetyl lysine and trifluoroacetyl lysine to incorporate them into proteins.^46,448−450^ Besides in vitro assays to elucidate the direct biological functions such as the roles of lysine acetylation in activating the deubiquitinase activity of OTU domain-containing protein 3 (OTUD3),^451^ and the kinase activity of the cell-cycle checkpoint kinase WEE1^452^ as well as inhibiting the enzymatic activity of cyclic GMP–AMP synthase (cGAS),^453^ GCE approaches for site-specific incorporation of AcK also hold significant potential for in vivo functional studies of acetylation in eukaryotic systems. For instance, Han et al.^66^ introduced a range of ncAAs, including AcK, into *Mus musculus* via stable integration of transgenes that encode a specially engineered *N*^ε^-acetyl-lysyl-tRNA synthetase (AcKRS) and tRNA^Pyl^ pair. They showcased the ability to control the timing and location of protein acetylation in various organs of the transgenic mouse model using recombinant green fluorescent protein (GFPuv). This innovative approach holds significant promise as a robust tool for conducting systematic, in vivo investigations of cellular proteins within the most widely used mammalian model organism for studying human physiology and disease. Also, Qin et al.^454^ incorporated AcK via GCE into IRF3/IRF7 DNA-binding domain (DBD) to create single and double site-specific fully acetylated proteins and demonstrated that the introduction of an acetyl group at precise sites within the interferon (IFN) regulatory factors IRF3/IRF7 DBD region inhibits the formation of IRF3/IRF7 liquid–liquid phase separation (LLPS) and the type I IFN (IFN-I) induction. Activation of SIRT1 by specific agonists successfully restored SIRT1 activity in aged mice, consequently reinstating IFN signaling and thwarting viral replication, unveiling a novel mechanism through which SIRT1 influences IFN production by modulating IRF3/IRF7 LLPS and offering insights into the factors contributing to age-related declines in innate immune function. In addition to AcK, lysine acetylation mimic such as 1,2,4-triazole amino acid (ApmTri) has been directly introduced into proteins through GCE by Kirchgäßner et al.^455^ Lysine acetylation neutralizes the charge of proteins bound by bromodomains (Brds). To mimic Kac and recruit Brds from the bromodomain and extraterminal domain (BET) family, they introduced a 1,2,4-triazole amino acid (ApmTri) as an alternative to the commonly used glutamine for simulating this modification. By optimizing the triazole substituents and side chain spacing, they achieved BET Brd recruitment to ApmTri-containing peptides with affinities comparable to those of natural substrates. Crystal structures of peptides containing ApmTri in complex with two BET Brds revealed a binding mode similar to that of Kac ligands. Thus, genetically encoding ApmTri in HEK293 cells allowed to produce ApmTri-containing recombinant proteins, which coenriched chromatin factor BRD3(2) which possess two adjacent Brds from cellular lysates.

Besides Kac, lysine acylation analogues and derivatives have been directly introduced into proteins through GCE, offering novel tools for investigating the biology of PTMs. For instance, Kcr is a PTM of histones associated with various epigenetic processes and diseases. To clarify the underlying mechanisms by which YEATS domains recognize Kcr involves amide−π and alkene−π interactions, Krone et al.^456^ investigated how the YEATS domain of human reader protein AF9 recognizes both Kac and Kcr by incorporating *para*-substituted phenylalanine derivatives (R = CH_3_, Cl, CN, or NO_2_) with distinct electrostatic properties at two specific positions (Phe28 and Tyr78) via GCE and revealed that the amide−π interactions between AF9 and acyllysines can be fine-tuned by altering the electron-rich nature of the aromatic rings, with more electron-rich rings resulting in more favorable interactions, which contrasts with amide–heteroarene interactions and offers valuable insights for potential therapeutic applications. Furthermore, they presented a novel discovery that CH−π interactions at Phe28 play a direct role in AF9 recognition of acyllysines, shedding light on the different features of YEATS domains. Notably, Phe28 is not highly conserved among these domains but has been shown to confer selectivity for specific PTMs. As another example, through employing chemical proteomic analysis, Zhang et al.^457^ discovered that various virulence factors encoded by *Salmonella typhimurium* are subject to acylation by short-chain fatty acids and a transcriptional regulator of pathogenicity island-1 (SPI-1), known as HilA, undergoes Kbu at specific lysine residues. They further utilized CRISPR-Cas9 gene editing and *M. barkeri* PylRSASF mutant (*Mb*PylRS-ASF) to genetically incorporate *N*^ε^-pent-4-ynyloxy-carbonyl-lysine (PycK) as a the stable mimic of Kbu into HilA and revealed that the site-specific Kbu modification of HilA has a profound impact on its genomic occupancy and expression of SPI-1 genes. Importantly, it attenuates the invasion of HeLa epithelial cells, as well as its dissemination in mice mesenteric lymph nodes (mLN) and liver. Furthermore, a *S. Typhimurium* strain carrying multiple-site lysine acylation modifications in HilA exhibited resistance to butyrate-mediated suppression of SPI-1 genes in microbiota-sufficient mice. These findings strongly indicated that significant microbiota-derived metabolites may directly acylate virulence factors, thereby impeding microbial pathogenesis in living organisms. Lastly, it is worth mentioning that the acylation of lysine side chains extends beyond acetylation to include a variety of modifications, such as short acyl chains like butyryl (bu) and crotonyl (cr), as well as fatty acids and charged functional groups.^458^ All of these modifications can be reversed by a relatively small group of lysine deacetylases (KDACs), which are classified into four protein families.^459,460^ However, only a limited number of KDACs display broad substrate promiscuity, capable of adding or removing this wide array of acylations. Spinck et al.^461^ devised a bacterial selection system for the directed evolution of KDACs based on the principle that deacetylation of orotidine-5′-monophosphate (OMP) decarboxylase Ura3 K93ac by sirtuins enables growth of *E. coli* in the absence of uracil. A critical lysine residue in the OMP decarboxylase was replaced with lysine derivatives, including AcK, BuK, and crotonyl-lysine(CrK) via GCE.^462^ They finally identified the evolved KDAC variants that exhibit massively greater selectivity for BuK compared to CrK. Leveraging the newly developed butyryl-selective KDAC variant, they shifted the cellular acylation profile toward an increased prevalence of lysine crotonylation, which will help to dissect the intricate roles played by various lysine acylations in the realm of cell physiology.

###### 4.3.1.1.3 Methylation of Lysine

Methylation can occur on residues of lysine, histidine and arginine. However, in contrast to lysine methylation, the methylation of histidine and arginine in physiological mechanism study using site-specific incorporation of methylated histidine/arginine via GCE has been largely unexplored so far. The initial discovery of lysine methylation occurs in histones, which plays a role in activating or silencing transcription, contingent upon the specific sites and methylation types (including mono-, di-, and trimethylation).^463^ Based on the established pSer orthogonal translation system to generate pSer-containing proteins,^108^ Yang et al.^463^ further developed a chemical method to dephosphorylate pSer residue to produce dehydroalanine (Dha), followed by metal-mediated conjugate additions of alkyl iodides to Dha, achieving chemoselective carbon–carbon bond formation to afford different lysine methylation modifications. Using this approach, they created variants of histone H3 with mono-, di-, or trimethylation at K79 and found these methylated histones promote transcription through histone acetylation. On the other hand, methylation of lysine on nonhistone proteins also plays vital functions in numerous cellular processes.^464^ To address the roles for methylation of a conserved lysine in the C-terminal domain of the molecular chaperone Hsp90, Rehn et al.^465^ introduced methylated lysine (metK) into C-terminal lysine K594 of Hsp90 through site-specific incorporation of the photocaged *N*^ε^-methyl-*N*^ε^(pnitrocarbobenzyloxy)lysine (met-ncbK) which gets decaged to metK via UV irradiation in living mammalian cells, and demonstrated that this modification alters the conformational cycle of Hsp90, which thus enables precise allosteric effects on other part of the protein far away from metK. Additionally, Wang et al.^466^ employed the amber suppression method to genetically encode an allysine precursor *N*^ε^-(4-azidobenzoxycarbonyl)-δ,ε-dehydrolysine (AcdK) in *E. coli* for convenient synthesis of both histone H3 and p53 proteins with site-specific lysine dimethylation through TCEP reduction and reductive amination, enabling the exploration of epigenetic enzyme functions such as histone demethylase LSD1 and histone acetyltransferase Tip60. These results confirmed LSD1 catalytic activity toward H3K4me2 and H3K9me2 but not H3K36me2, while methylation at K372 of p53 directly triggers Tip60, promoting acetylation at K120 of p53. Similar to lysine methylation, arginine methylation can occur at varying levels, resulting in the formation of monomethylarginine and dimethylarginine. However, the site-specific incorporation of methylated arginine analogues through GCE in vivo has been unexplored yet. Specifically, the incorporation of monomethylarginine has only been successfully achieved in an in vitro translation system using yeast arginyl-tRNA synthetase.^467^ Importantly, both monomethylarginine and dimethylarginine can be chemically introduced into recombinant proteins using the versatile Dha.^468^

###### 4.3.1.1.4 Ubiquitin and Ubiquitin-Like of Lysine

Ubiquitin, a 76-amino acid linear polypeptide, plays a pivotal role in the PTM of target proteins, as well as ubiquitin-like (e.g., SUMO) proteins, which collectively regulate nearly all facets of eukaryotic biology.^469^ One of the most noteworthy functions is its involvement in the protein degradation.^470^ Several strategies have been devised to generate proteins modified with ubiquitin or ubiquitin-like proteins at specific sites. Virdee et al.^471^ leveraged genetically encoded orthogonal protection and activated ligation to synthesize site-specific ubiquitination of recombinant protein. BocK was initially introduced into the target protein through GCE and the remaining amino groups are safeguarded using benzyloxycarbonyl or carbobenzoxy (Cbz) group. The removal of Boc unmasks the sole amino functionality on the target protein. This exposed amine then reacts with a Cbz-protected ubiquitin thioester, resulting in site-specific ubiquitylation of the target protein after a global Cbz deprotection step. Utilizing this method, they further made a significant discovery by demonstrating that the deubiquitinase TRABID, previously believed to be selective for K63-linked ubiquitin, cleaves the K29 linkage 40-fold more efficiently than the K63 linkage.^472^ More recently, Madrzak et al.^473^ applied a similar strategy to show that ubiquitylation of the human Dvl2 DIX domain at K54, but not K58, hinders its polymerization in solution, thereby preventing the formation of signalosomes. Zang et al.^474^ discovered and validated a previously unreported form of protein PTM, known as aminoacylated lysine ubiquitination, which involves the attachment of a ubiquitin group to the α-amine group of aminoacylated lysine. Using genetically encoded methionyl lysine and other aminoacylated lysine analogues as specialized probes, over 2000 ubiquitination sites on all 20 aminoacylated lysines in two distinct human cell lines were identified, thus demonstrating that the ubiquitin-conjugating enzyme UBE2W serves as the catalyst for aminoacylated lysine ubiquitination. This breakthrough not only sheds light on aminoacylated lysine ubiquitination but also lays the foundation for the detection and validation of novel protein PTMs through GCE strategy.

###### 4.3.1.1.5 Sulfation of Tyrosine

Tyrosine sulfation represents a generally considered irreversible PTM playing vital roles in a range of biomolecular interactions, such as chemotaxis, anticoagulation, viral infection, plant immunity and cell adhesion.^475,476^ Using recombinant sulfo-hirudin generated in *E. coli* combined with evolving *Mj*TyrRS, Liu et al. discovered that tyrosine sulfation significantly enhances the affinity of hirudin for human thrombin, increasing it over 10-fold.^477^ Additionally, Schwessinger et al. developed a recombinant RaxX containing sulfotyrosine (sTyr) which induce immunity in rice plants and thus protecting rice plants from bacterial pathogen.^478^ Moreover, two research groups^172,479^ independently evolved the *E. coli*-derived tyrosyl-tRNA synthetase/tRNA (EcTyrRS/tRNA) pair using distinct directed evolution and selection methods for site-specific incorporation of sTyr in eukaryotic cells. Specifically, Italia et al.^172^ achieved site-specific incorporation of sTyr into human heparin cofactor II in HEK293T cells, revealing distinct roles for the two sulfation sites (Y60/Y73) in modulating the protein interaction with glycosaminoglycans and thrombin, while He et al.^479^ harnessed the evolved *Ec*TyrRS/tRNA for genetic encoding of sTyr into chemokine receptor CXCR4 and demonstrated the essential role of Y21 sulfation in CXCR4-mediated signaling transduction in HEK293T cells. Recently, Chen et al.^189^ created autonomous cells, both prokaryotic and eukaryotic, with the ability to biosynthesize and genetically encode sTyr, and finally used the autonomous *E. coli* chassis for preparation of therapeutic sTyr-containing thrombin inhibitors with enhanced efficacy.

###### 4.3.1.1.6 Arginine Citrullination

Citrullination is a process in which the guanidinium group of arginine undergoes hydrolysis, transforming into a neutral urea group and giving rise to the formation of citrulline. This modification, a pivotal PTM of arginine essential for various physiological functions such as gene regulation and neutrophil extracellular trap formation,^480^ remains a challenge in protein studies due to the difficulty of homogeneously citrullinating proteins at specific sites. Mondal et al.^481^ presented a novel technique allowing precise integration of citrulline (Cit) into mammalian cell proteins. This method leverages an engineered *E. coli*-derived leucyl tRNA synthetase-tRNA pair that introduces a photocaged-citrulline (SM60) bearing an *o*-nitrobenzyl photocaging group into proteins. Subsequent light exposure converts SM60 to Cit both in vitro and within living cells. To showcase its efficacy, a biochemical analysis was conducted by incorporating Cit at two known autocitrullination sites (R372/R374) in protein arginine deiminase 4 (PAD4) and revealed that the R372 Cit and R374 Cit mutants are significantly less active (181- and 9-fold, respectively) compared to the wild-type enzyme. This breakthrough technology holds promise in unraveling the intricacies of citrullination biology.

##### 4.3.1.2 Site-Specific Cross-Linking in Situ for Research of Biological Mechanism

Studying interactions between proteins and other molecules, including protein–protein interactions (PPIs), is crucial but challenging. Covalent ncAAs have significantly advanced this field by serving as proximity probes that can be activated to form covalent bonds with nearby molecules, such as specific residues from proteins or enzyme substrates.^3^ These bonds stabilize weak or transient protein complexes, allowing for detailed analysis through techniques like affinity mass spectrometry or computational modeling.^482,483^ Here we spread out by listing amino acids possessing different chemical groups, which are highly paralleled with their functions.

**Figure 19:**
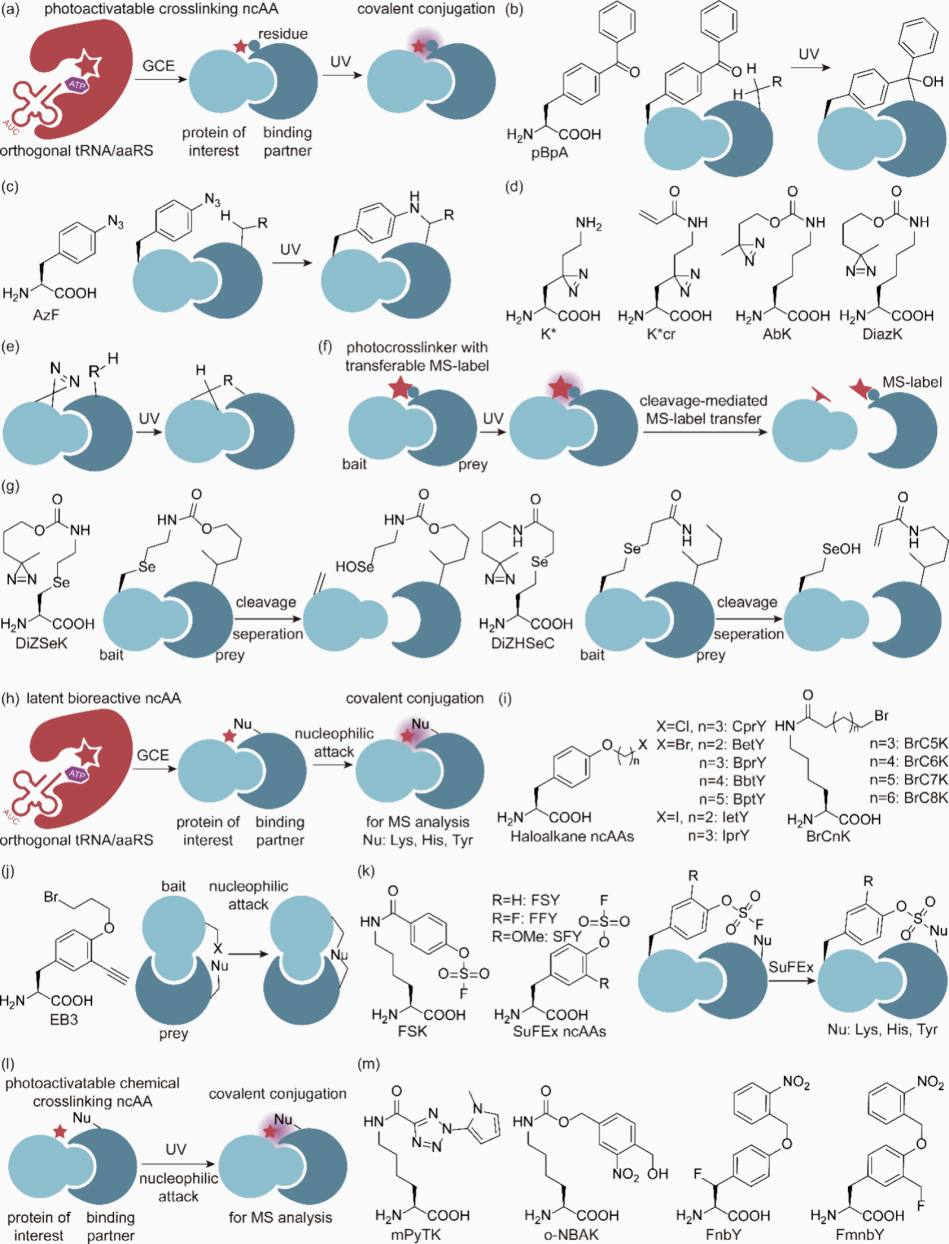
Site-specific cross-linking in situ for research of biological mechanism. (a) Schematic illustration of photoinduced site-specific cross-linking in situ. The photoactivatable cross-linking ncAA on the protein of interest can be activated by UV and covalently onjugate with nearby residue on the binding partner protein. (b) Chemical structure (left) of the photoactivatable cross-linking ncAA pBpA and its cross-linking reaction (right). (c) Chemical structure (left) of the photoactivatable cross-linking ncAA AzF and its cross-linking reaction (right). (d) Chemical structures of diazirine-containing lysine analogues as photoactivatable cross-linking ncAAs. (e) Photoactivated cross-linking reaction for diazirine-containing lysine analogues. (f) Schematic illustration of photoinduced site-specific cross-linking followed by MS-label transfer. After conjugating with the prey protein by UV light, the ncAA can cleave by processing and leave a MS label on the prey protein. MS, mass sprectrum. (g) Chemical structures and their cross-linking reactions of the diazirine-containing self-releasable lysine analogues DiZSeK (left) and DiZHSeC (right). (h) Schematic illustration of chemical cross-linking in situ. The latent bioreactive ncAA on the protein of interest can react with nearby residues such as Lys, His and Tyr. (i) Chemical structures of haloalkane ncAAs as latent bioreactive ncAAs. (j) Chemical structure of the haloalkane ncAA EB3 (left) and the cross-linking reaction on haloalkane ncAAs (right). (k) Chemical structures of SuFEx-based amino acids (left) and their cross-linking reactions (right). (l) Schematic illustration of photoactivated chemical cross-linking. The photoactivatable latent bioreactive ncAA on the protein of interest can react with nearby residues upon activation by UV light. (m) Chemical structures of photoactivated chemical cross-linking amino acids.

One of the most impactful advancements in this area is the use of photoactivatable ncAAs (Figure 19a), which include benzophenone (e.g., pBpA), aryl azide (e.g., AzF), and diazirine (e.g., K*, K*cr, AbK, and DiZPK) (Figure 19b,c,d). These ncAAs form covalent bonds upon exposure to UV-A light. Benzophenone generates a diradical capable of integrating into C–H bonds (Figure 19b).^484,485^ For instance, pBpA has been used to map the interaction of protein with DNA and to explore the structural details of protein complexes.^486−490^ Aryl azide, less likely to interfere with protein functions, is effective for mapping ligand–receptor interfaces and visualizing protein complexes (Figure 19c).^491−493^ Diazirine, being small and hydrophilic, minimizes binding impact and has been used to explore histone acylations and discover novel interactions in small proteins (Figure 19d,e).^494−497^

To decrease the signal background of bait protein in high concentrations, diazirine amino acids containing releasable photo-cross-linkers were developed (Figure 19f,g).^498^ They include a selenium atom, which, after photo-cross-linking, can be oxidatively cleaved to separate the bait and prey protein, generating a selenenic acid (SA) moiety on the prey protein. This SA moiety can then be captured by tagging with an alkynyl-SA scavenger molecule suitable for CuAAC-labeling with an azide-bearing tag for analysis. Lin et al. and Yang et al. developed a robust and precise cleavage-and-capture after protein photo-cross-linking strategy by utilizing these two amino acids.^499,500^

In addition to photoactivatable ncAAs, chemical cross-linkers like alkyl halides and sulfur-fluoride exchange (SuFEx) reagents provide another method for capturing protein interactions (Figure 19h). These cross-linkers remain stable under physiological conditions but react with nucleophilic amino acids such as cysteine, lysine, histidine, serine, and tyrosine. This reactivity is contingent upon the spatial arrangement of these amino acids within the three-dimensional structure of a protein or during protein–protein interactions.

Alkyl halide cross-linkers, such as (*S*)-2-amino-3-(4-(3-bromopropoxy)phenyl)propanoic acid (BprY) and its derivatives, are used to covalently link interacting proteins to stabilize them for identification or structural analysis (Figure 19i,j).^454,482,483,487,501,502^ Interestingly, Jacob et al. incorporated a bromine-containing amino acid, *O*-2-bromoethyl tyrosine (O2beY), into an enzyme to conjugate with a nearby cysteine within this enzyme.^503^ With computational-aided design, the thermostabiligy of this enzyme could be tuned. SuFEx reagents, such as fluorosulfate-l-tyrosine (FSY), fluorosulfonyloxybenzoyl-l-lysine (FSK) and *o*-sulfonyl fluoride-*O*-methyltyrosine (SFY), could play roles in a complementary manner regarding the side chains, distances and orientations of the targeting amino acids and have been used to capture elusive enzyme–substrate interactions like protein–nucleic acid interaction (Figure 19i,j).^335,340,504^ FSY’s target residues are often found at protein surface and interface; FSK could cross-link amino acids in a farther distance; FSY and SFY could also covalently capture m^6^ A-containing RNAs in vivo, contributing to epigenetics research with other amino acids.^339,505^

Recent developments in photoactivatable chemical cross-linkers, such as *N*-methylpyrroletetrazole-lysine (mPyTK) and *o*-nitrobenzyl-alcohol-lysine (o-NBAK) have further advanced this field (Figure 19l,m).^506−508^ They are photoactivatable and have selective reactivity and have facilitate the discovery of protease and hydrolase substrates. Future advancements in ncAA cross-linkers and their applications are expected to lead to more sophisticated and targeted approaches in studying complex biological systems.

##### 4.3.1.3 Introducing Site-Specific Fluorophores in Situ for Study of Biological Mechanisms

Protein structures are the foundation for us to understand many biological mechanisms. However, techniques like X-ray diffraction and cryo-electron microscopy are hard to capture dynamic physiological state, and fusing target proteins with fluorescent proteins or tags are not available on some packed proteins or complicated proteins. GCE addresses this issue by enabling the site-specific introduction of fluorophores into proteins in situ with minimal disturbance,^509−511^ allowing researchers to precisely track and identify dynamic physiological states using microscopes.^512−514^ Experiments using these techniques can be conducted with either purified proteins or in living cells.^515,516^

**Figure 20:**
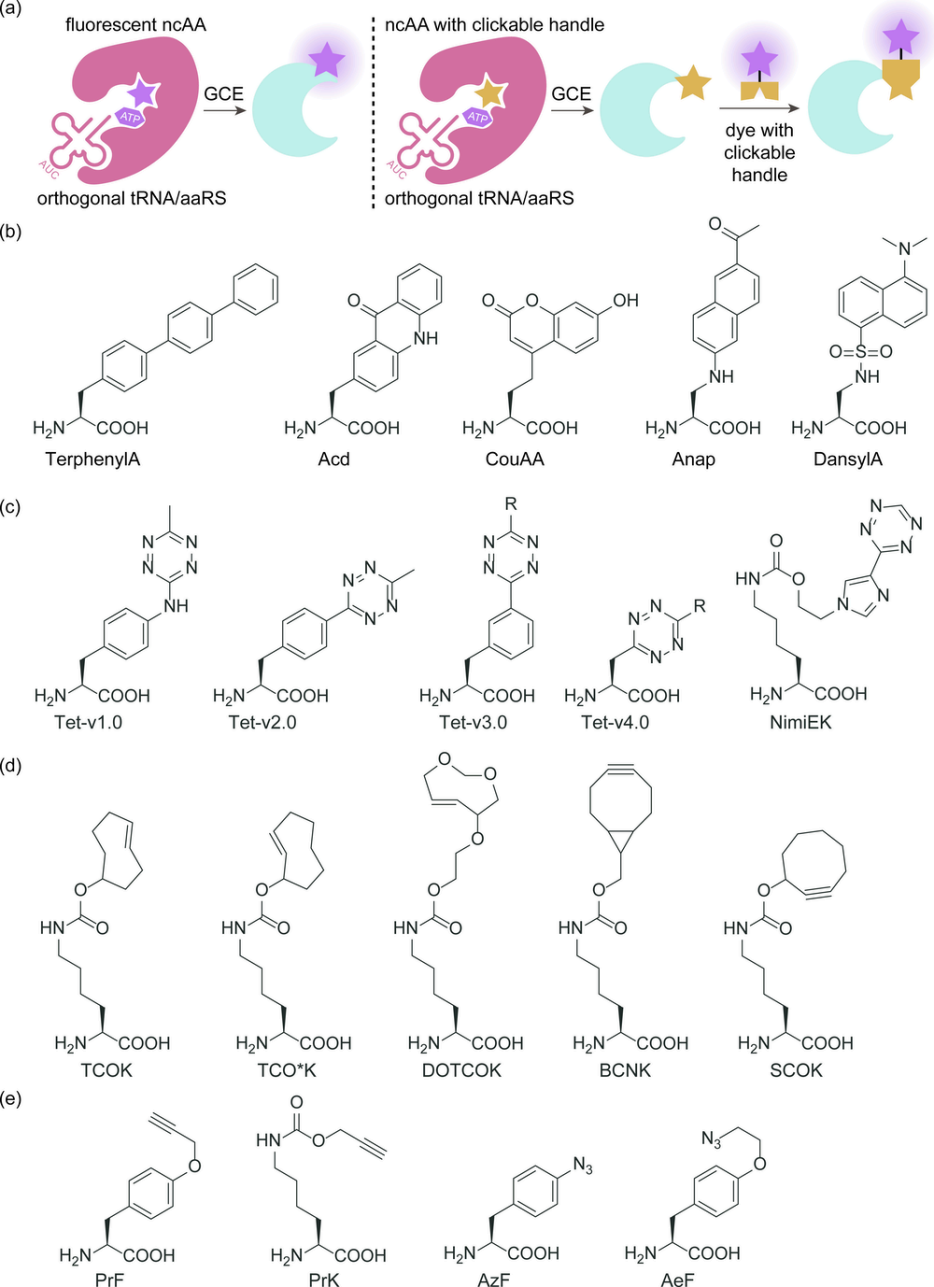
Introducing site-specific fluorophores in situ for research of biological mechanisms. (a) Schematic illustration of enlightening proteins with fluorescent ncAAs (left) or ncAAs with reaction handles (right). The ncAAs with reaction handles on proteins can react with fluorescent dyes with reaction handles. (b) Chemical structures of commonly used fluorescent ncAAs. (c) Chemical structures of tetrazine-containing ncAAs used for imaging. (d) Chemical structures of commonly used strained dienophile-containing ncAAs for SPIEDAC reaction. (e) Chemical structures of commonly used ncAAs for SPAAC or CuAAC reaction.

In this section, we aim to summarize the elements necessary to conduct this type of research, including strategies for introducing and utilizing fluorophores and their relevant applications. These strategies involve two kinds of ncAAs (Figure 20a): the first type includes amino acids with inherent fluorescent side chains, and the second type includes amino acids capable of conjugating with chemicals containing fluorophores. We also discuss strategies for using these fluorophores, such as observing spatial fluorescence distribution patterns and dynamic fluorescence changes, with the assistance of advanced imaging tools like super-resolution microscopy (SRM).

4.3.1.3.1. Genetically Encoded Fluorescent Amino Acids. A series of fluorescent amino acids have been genetically encoded in different cells, such as 3-((6-acetylnaphthalen-2-yl)amino)-2-aminopropanoic acid (Anap) and 2-amino-3-((5-(dimethylamino)naphthalene)-1-sulfonamido)propanoic acid (DansylA) for eukaryotes, l-(7-hydroxycoumarin-4-yl) ethylglycine (CouA), l-2-acrydonylalanine (Acd), and 4-biphenyl-l-phenylalanine (terphenylA) for prokaryotes (Figure 20b).^2,512^ Some of these amino acids are sensitive to their environment. For example, Anap has an emission maximum that shifts in different solvents, making it useful for investigating protein dynamics, especially in membrane proteins.^517−520^ Similarly, DansylA and CouA show sensitivity to their environment, such as solvent polarity and pH, enabling the monitoring of protein dynamics and conformational changes.^521−524^

Other fluorescent amino acids are not environmentally sensitive but possess unique properties. For instance, Acd has high quantum yield and photostability, making it suitable for confocal microscopy.^525−528^ Additionally, terphenylA has a long fluorescence lifetime and unique emission spectra, making it useful for applications like two-photon fluorescence microscopy.^529−531^

4.3.1.3.2. Amino Acids with Reaction Handles for Imaging. The ncAAs with bioorthogonal reaction handles are widely used to conjugate with fluorescent dyes for structural and mechanistic studies (Figure 20c–e),^513^ although there are a few other reactions available.^532^ Although alternative reactions are available, these bioorthogonal reactions are preferred for their rapid kinetics, selectivity, and biocompatibility.^514^ As there are many good-quality reviews^311,510,513,514,533,534^ and articles^509,535^ that discuss bioorthogonal reactions and ncAAs, here we focus on three reactions commonly used because of their fast kinetics and high bioorthogonality.

The primary and favored method is the strain-promoted inverse electron-demand Diels–Alder cycloaddition (SPIEDAC). It is the fastest bioorthogonal reaction with 1–10^4^ M^–1^ s^–1^ of rate constant and has become the preferred choice for use in living cells.^311,513,534^ This reaction employs *trans*-cyclooctene as a dienophile and tetrazine as a diene. Numerous ncAAs have been developed as *trans*-cyclooctene or tetrazine derivatives, including *N*^ε^-(((*trans*-cyclooct-4-en-1-yl)oxy)carbonyl)-l-lysine (TCOK),^536,537^*N*^ε^-(((*trans*-cyclooct-2-en-1-yl)oxy)carbonyl)-l-lysine (TCO*K),^538^*N*^ε^-((bicyclo[6.1.0]non-4-yn-9-ylmethoxy)carbonyl)-l-lysine (BCNK),^537,539^*N*^ε^-((cyclooct-2-yn-1-yloxy)carbonyl)-l-lysine (SCOK),^538,540^*N*^ε^-((2-((*trans*-5,8-dihydro-4*H*-1,3-dioxocin-5-yl)oxy)ethoxy)carbonyl)-l-lysine (DOTCOK),^541^ Tet-v1.0,^542^ Tet-v2.0,^543^ Tet-v3.0,^544^ Tet-v4.0,^545^ and NimiEK.^546^ An additional advantage of SPIEDAC labeling is the self-quenching property of tetrazine dye, which reduces the fluorescent background.^547−549^

Cu-catalyzed azide alkyne cycloaddition (CuAAC) is also significant, utilizing simple and small functional group with relatively fast kinetics (*k* = 10–100 M^–1^ s^–1^). Several amino acids have been developed, such as *N*^ε^-[(2-propynyloxy)carbonyl]-l-lysine (PrK),^550^ propargyloxy-l-phenylalanine (PrF),^551^*p*-azido-l-phenylalanine (AzF),^552^ 4-(2-azidoethoxy)-l-phenylalanine (AeF).^553^ However, the toxicity of high concentration of Cu(I) catalyst and the requirement of ligands limited their applications on living cells. Consequently, strain-promoted azide alkyne cycloaddition (SPAAC) was developed, which only employs azide and cyclooctyne derivatives and offers slower kinetics (*k* = 10^–3^ – 60 M^–1^ s^–1^).^554^

4.3.1.3.3. Exploring Mechanisms by Observing Spatial Fluorescence Distribution Patterns. After the site-specific incorporation of fluorophores using the above ncAAs, observing strategies are essential for gathering research data. Generally, there are two approaches: observing spatial fluorescence distribution patterns and monitoring dynamic fluorescence changes. These strategies are often employed in conjunction with advanced imaging technologies.

The first approach focuses on utilizing the spatial distribution of introduced fluorophores to delineate protein distribution patterns. This is particularly useful for proteins that are densely packed or complex, making them challenging to tag with fluorescent markers.^79,555^ The development of imaging technologies with higher resolution, such as STED, STORM, and SIM, enhances the strength of GCE and this strategy.^556^ For example, minimal photon fluxes (MINFLUX) and minimal stimulated emission depletion (MINSTED) are cutting-edge super-resolution microscopy techniques that achieve nanoscale imaging by precisely localizing fluorescent molecules with minimal photon usage and employing optimized depletion strategies to confine fluorescence emission, respectively.^79,557^ MINFLUX attains three-dimensional precision <3 nm (SD) in biological samples, facilitating the imaging of β-actin cytoskeleton, a tightly packed structure, through GCE-based bioorthogonal reaction labeling.^79,558^ STORM (stochastic optical reconstruction microscopy) and dSTORM (direct STORM) achieve nanoscale resolution by randomly activating and precisely localizing individual fluorescent molecules to reconstruct detailed, high-resolution images.^559−561^ dSTORM has been applied to visualize the nanoscale spatial distribution of ion channels in live neurons,^555,562^ the aggregation formation of cell surface receptors,^563^ the production of nuclear actin filaments,^564^ and the location of bacterial effector proteins within host cells by GCE-based SPIEDAC labeling.^49^ SIM (structured illumination microscopy) is a super-resolution imaging technique that enhances resolution by using patterned light to illuminate the sample,^565^ and it has also been used in similar methods.^566^ Another interesting research involves using photocaged amino acids like ONBY to mask and unmask key residues, allowing spatiotemporal control of protein activity and self-organization for research under total internal reflection fluorescence (TIRF) microscopy.^567^ With higher ncAA incorporation efficiency and advanced imaging technologies, this GCE strategy offers new avenues for fundamental studies of protein functions.

4.3.1.3.4. Exploring Mechanisms by Observing Fluorescence Change. The second strategy for research, observing the dynamic fluorescence changes, is widely utilized for biological mechanistic studies. Several methods have been developed based on the property of ncAAs, chemical reactions, and resonance energy transfer. Here we highlight some methods on recently published or high-impact articles.

**Figure 21:**
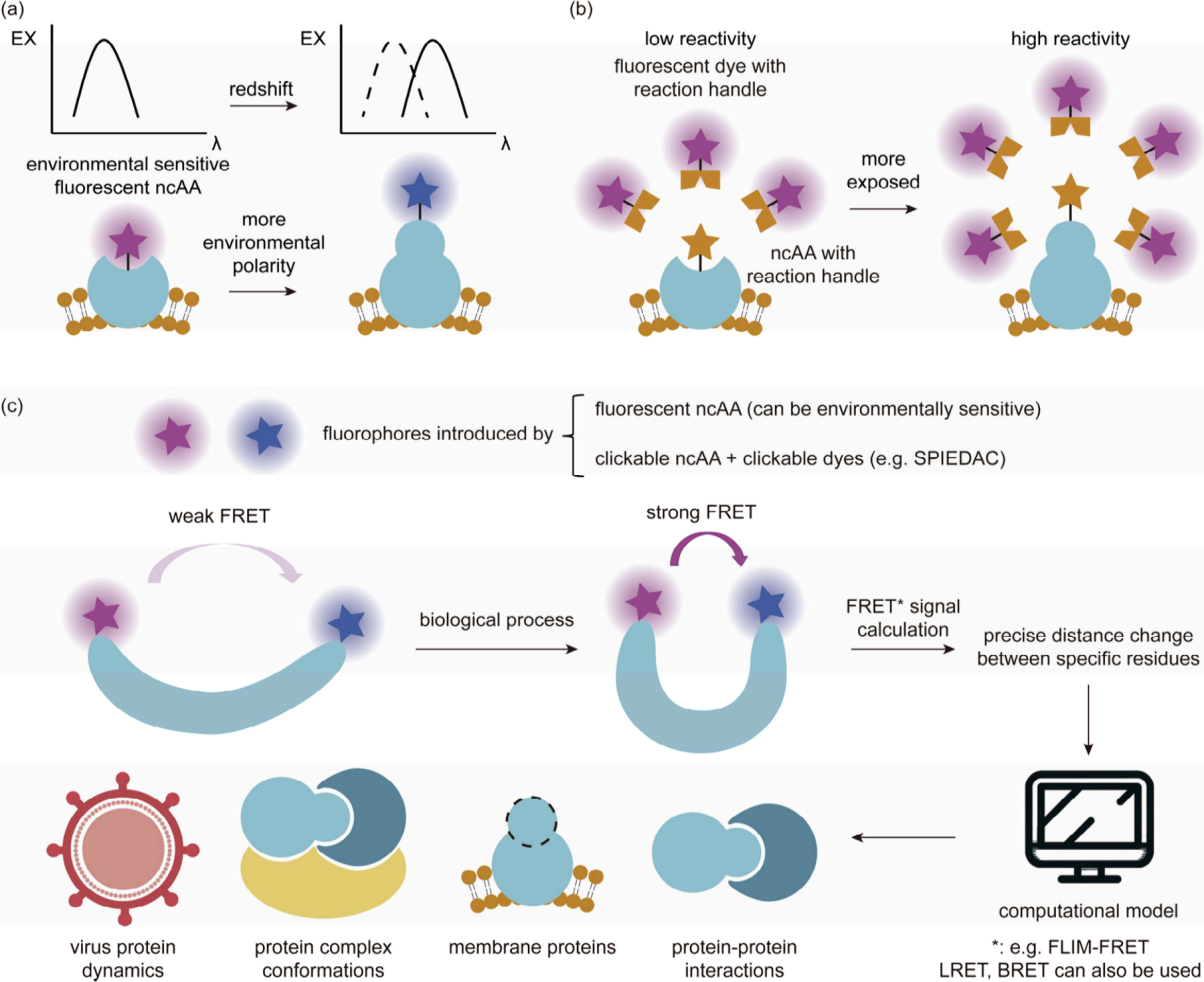
Schematic illustration of strategies of using fluorophores to study biological mechanisms. (a) Schematic illustration of the redshift of fluorescent ncAA induced by enhanced environmental polarity. EX, excitation. (b) Schematic illustration of different reactivities of differently exposed ncAAs with reaction handle. More exposed ncAA have higher reactivity. (c) Schematic illustration of using smFRET on introduced fluorophores to study biological mechanisms. Fluorophores are introduced at specific sites using fluorescent ncAA or clickable ncAAs and dyes. During a biological process, the distance between these residues changes, leading to a change of FRET signal. Inputting these into computational models can help to work out protein or protein complex conformation changes. BRET, bioluminescence resonance energy transfer; LRET, luminescence (or lanthanide-based) resonance energy transfer.

The first method relies on the fluorescent ncAAs that exhibit optical properties sensitive to the polarity of their local environment (Figure 21a). For example, the emission maxima of Anap shifts to longer wavelength when exposed to solvents with increasing polarity, indicating changes in solvent accessible surface area (SASA) on specific residues during biological processes. This sensitivity facilitates the exploration of protein dynamics, particularly for the conformational changes of ion channels like TRPM8 and TRPV1 in biological processes, providing insights into their activation and desensitization mechanisms.^517,568−572^ Additionally, 4-cyano-phenylalanine (Phe_CN_) can quench the fluorescence of nearby tryptophan residues, enabling researchers to monitor distance changes between key residues in proteins.^573,574^

The second method assesses the reaction efficiencies between clickable fluorophores and clickable ncAAs at different sites of proteins. As more solvent-exposed residues exhibit higher labeling efficiency, the reaction efficiencies can reveal the environmental conditions of specific residues on membrane proteins like GPCRs (Figure 21b).^575,576^

The third method relies on the single-molecule Förster resonance energy transfer (smFRET) signals. The smFRET is a powerful tool for studying protein conformational changes and complex assembly, particularly in dynamic biological processes. This method uses two site-specifically introduced fluorophores or one fluorophore with a fluorescent protein to form a FRET pair.^577^ The intensity of FRET correlates with the distance between the fluorophores, providing detailed distance measurements that can be mapped onto protein structures (Figure 21c). Advanced imaging technologies like fluorescence lifetime imaging microscopy (FLIM-FRET) can offer precise distance measurements, leveraging the strengths of GCE to provide precise distance measurements.^578^

One of the wide-ranging applications of smFRET on GCE is in the study of dynamic conformations of viruses, which has multiple conformations in biological processes but faces engineering challenges like compact genome and overlapping open reading frames.^579^ For example, Das et al. introduced two TCOK at different positions of the influenza HA2 subunit and attached Cy3 and Cy5 fluorophores via SPIEDAC reaction. They performed smFRET to measure the distance between the fluorophores and monitor HA’s conformational dynamics during membrane fusion, finding that HA can reversibly interconvert between three distinct conformations regulated by pH, receptor binding, and target membrane interaction.^580^ Similarly, Lu et al. used TCOK on V1 and V4 Env variants of HIV-1, reacted with Cy3B or Cy5 fluorophores, and performed smFRET to measure distances between the fluorophores. They discovered that native Env on the virus preferentially samples the pretriggered conformation (state 1), unlike soluble and stabilized Env trimers which predominantly occupy downstream conformations (states 2 and 3).^516^

This method also facilitates the study of large protein complexes. Yu et al. incorporated TCOK at specific sites of NUP98 in the nuclear pore complex (NPC) and labeled them with donor and acceptor dyes.^515^ Using FLIM-FRET, they probed the distance distribution of NUP98 segments inside the NPC, obtaining insights into their distribution and motion. Desai et al. utilized AzF to conjugate with Cy3 and Cy5 to generate smFRET signals, revealing conformational changes in the ribosome during translation.^581^ Voith von Voithenberg et al. employed a three-color FRET system with AzF conjugated Atto488 in Hsp70s, providing evidence for key differences in the conformational cycle of Hsp70s.^582^

smFRET can not only detect conformations of proteins like EGFR, but also be effective in studying protein–protein interactions (PPIs).^583^ It has been used for studying PPIs in enzyme–substrate recognition and large protein complexes.^584,585^ Combining FRET methods with the environmental sensitivity of Anap, PPIs on membrane proteins like ion channels could be further depicted.^569^ Researchers also use photocaged ncAAs to block and recover protein complex interactions and measure FRET changes.^586^

Except for FRET, other methods like bioluminescence resonance energy transfer (BRET) and luminescence (or lanthanide-based) resonance energy transfer (LRET) have also been adapted for research. BRET avoids issues like photobleaching and autofluorescence by using a bioluminescent donor and a fluorescent acceptor.^587,588^ For instance, BRET was used to detect conformational changes in Frizzled receptors.^589^ LRET, involving transfer from a lanthanide donor to an organic fluorophore or fluorescent protein acceptor, allows time-resolved detection due to the long-lived luminescence of lanthanide complexes.^590^ This method has been used to model the complex between AMPA receptors and stargazin by measuring LRET lifetimes.^532^

In summary, with the combination of a variety of ncAAs to introduce fluorophores, strategies and methods to observe them, and advanced imaging techniques, researchers have uncovered many biological mechanisms. With the development of these elements, GCE based imaging techniques hold promise for the research of protein dynamics, protein complex, protein distribution, PPI, etc.

##### 4.3.1.4 Nuclear Magnetic Resonance (NMR) Probes

NMR spectroscopy provides distinct advantages for studying dynamic and complex biological systems at atomic resolution, allowing for analysis under both physiological and native conditions.^591^ GCE presents a practical approach for NMR spectroscopists to incorporate spectroscopic probes including fluorine-19 (^19^F)-based NMR probes, trimethylsilyl (TMS)-based NMR probes and isotope-based NMR probes, in a site-specific manner. Since the incorporation of ^19^F-containing ncAA 3,5-difluorotyrosine (F2Y) into β-arrestin 1 (β-arr1) induces minimal disturbances to the overall structure and results in more substantial chemical shifts compared to the conventional bromo-4(trifluoromethyl)acetanilide (BTFA)-labeled cysteine-negative arrestin, and the phenolic F2Y oxygen’s sensitivity to environmental changes could increase fluorine-19 nuclear magnetic resonance (^19^F-NMR) signal, Yang et al.^592^ introduced F2Y at specific positions in β-arr1 recombinantly expressed in *E. coli* via GCE^593^ combined with ^19^F-NMR and demonstrated that β-arr1 deciphers receptor phospho-C-tail signals through its phosphate-binding concave surface, which contains at least 10 potential phosphate-binding sites. Using the same method, they^594^ further observed alterations in the three proline regions (PRs) of β-arr1 when exposed to diverse receptor phospho-C-tail stimuli, revealing that allosteric mechanisms are crucial in regulating the specific conformational states and functional outcomes of PRs in β-arr1 which interact with SH3-domain proteins, and the NMR chemical shifts observed in arrestin PRs matched the intrinsic efficacy and specificity of SH3 domain recruitment. On the other hand, while site-specific labeling with ^19^F-NMR probes has been a significant advancement for membrane protein complex studies,^595−597^ its reliance on cysteine-mediated chemical labeling limits observations to protein surface residues, omitting crucial interactions within hydrophobic cores, and necessitates altering other surface-exposed cysteine residues, potentially impacting protein structure and function. Although ^19^F-NMR has broad applications in protein structure and dynamics characterization, studying large transmembrane protein complexes remains a challenge due to complex NMR spectra with severe line broadening and overlapping resonances. Wang et al.^598^ developed another novel method in eukaryotic cells for the utility of a genetically encoded ^19^F NMR probe in exploring eukaryotic membrane protein dynamics. This method involves genetic integration of another ^19^F-containing ncAA 3′-trifluoromethyl-phenylalanine (tfmF) via GCE^46^ into cannabinoid receptor 1 (CB1) at sensitive sites without significant structural alterations using the Baculovirus expression system.^599^ Through ^19^F NMR and X-ray crystallography methods, they discovered a new preactive state of CB1, stabilized by the allosteric modulator Org27569 and agonists, suggesting a unique model of allosteric modulation. Similar tfmF-based ^19^F NMR method was used by Chan et al.^600^ for cotranslational folding studies, permitting direct, background-free observation of how the ribosome stabilizes partially folded intermediate states of the FLN5 immunoglobulin-like domain of a nascent multidomain filamin FLN^601,602^ during translation, providing insights into cotranslational folding process and the ribosome’s role in protein biosynthesis.

Despite these significant progresses in combination of ^19^F-NMR with GCE technology for studying protein dynamic conformation changes, ^19^F-NMR spectroscopy typically demands a substantial quantity of protein (usually exceeding 100 μM), with each measurement taking over 12 h. Liu et al.^603^ presented another novel approach termed DeSipher method using genetically encoded 4-trimethylsilyl phenylalanine (TMSiPhe) in combination with ^1^H NMR spectroscopy to detect conformational changes in biological systems, requiring only less protein amount and spectrum accumulation time compared to ^19^F-NMR method. The trimethylsilyl (TMS) groups serve as excellent NMR probes for biological macromolecules, generating intense singlets in ^1^H NMR spectra near 0 ppm, a region with minimal interference from other proton resonances. The distinctive upfield ^1^H NMR chemical shift and the remarkably efficient incorporation of TMSiPhe into β-arr1 allowed them to explore multiple conformational states within a phospho-β2 adrenergic receptor/β-arr1 membrane protein signaling complex, subsequently demonstrating that extracellular ligands induced conformational alterations situated in the polar core or ERK interaction site of β-arr1, driven by direct interactions with transmembrane core of the receptor. He et al.^604^ further combined FRET with the same DeSipher method by incorporating TMSiPhe into arrestin2, and revealed an interdependent mechanism in phospho-receptor-arrestin interactions, highlighting the complex role of phosphorylation in modulating arrestin functions. Additionally, Abdelkader et al.^78^ optimized the ^1^H NMR method by developing a system for incorporating another TMS-containing ncAA, *N*^6^-(((trimethylsilyl)methoxy)carbonyl)-l-lysine (TMSK), into proteins for NMR analysis. TMSK acts as an effective NMR probe for examining protein–protein interactions and ligand–protein binding due to its distinct signal in ^1^H NMR spectra without need of isotope labeling for resonance detection and assignment. On the other hand, due to the homotypic nature of homorepeat (HR) proteins, high-resolution structural and dynamic characterization is generally inaccessible, Urbanek et al.^605^ devised an isotope-based NMR probe for isotopically labeling individual glutamines within HRs by site-specifically introduce a unique [^15^N,^13^C]-labeled glutamine in combination of GCE^16^ and cell-free protein synthesis.^606^ This method has empowered them to employ NMR techniques to investigate polyglutamine (poly-Q) HR huntingtin^607^ exon1 containing a 16-residue poly-Q region, revealing the presence of an N-terminal α-helix under near-neutral pH conditions, which gradually disappears toward the end of the HR. And the versatility of this strategy was also demonstrated by introducing labeled glutamine into a pathogenic form of huntingtin with 46 glutamines.

##### 4.3.1.5 Monitoring Voltage Changes to Study Biological Mechanisms

**Figure 22:**
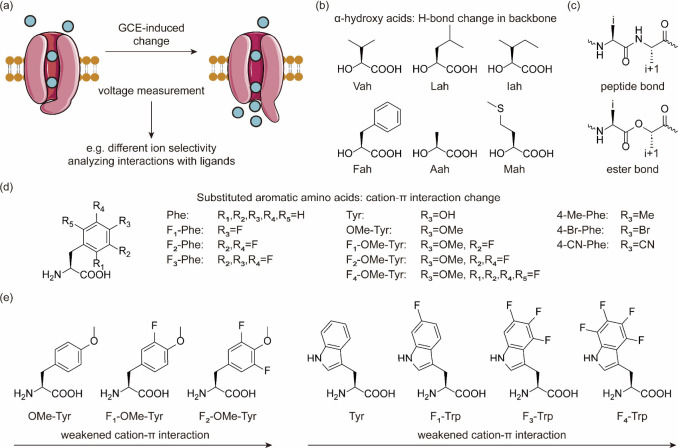
Monitoring voltage changes for study of biological mechanisms. (a) Schematic illustration of GCE-induced change for voltage measurement. Special ncAA is introduced to key sites of proteins like ion channels, leading to changes of protein functions. (b) Chemical structures of α-hydroxy acids. (c) The change of H-bond in backbone when introducing α-hydroxy acids (lower) compared to canonical amino acids (upper). (d) Chemical structures of substituted aromatic amino acids. (e) The change of cation−π interaction when introducing substituted aromatic amino acids.

Incorporating ncAAs at key sites on ion channels can affect their conformations and functions. By measuring voltage changes and observing structural models, researchers can identify the role of specific residues, enhancing our understanding of ion channels (Figure 22a).

Many studies have utilized α-hydroxy acids to investigate ion channel function (Figure 22b,c). Infield et al. used α-hydroxy acids to substitute amide bonds with ester bonds in the S4 segment of Shaker potassium channels and found it can disrupt voltage-gating, highlighting the critical role of main-chain chemistry in voltage sensing.^608^ Leisle et al. replaced pore-lining residues in ion channels with α-hydroxy acids and discovered that backbone amides are the key for Cl^–^ specificity in these channels.^609^

Other studies have used aromatic amino acid analogues (Figure 22d,e). Blom et al. added fluorinated aromatic amino acid analogues to key sites in the nicotinic acetylcholine receptor binding site and found that these different cation−π and hydrogen bonding patterns altered binding patterns and receptor activation.^610^ Similarly, Knox et al. used fluorinated O-Me-Tyr analogues in the nicotinic acetylcholine receptor and developed models for how ACh and cytisine bind to the receptor.^611^

##### 4.3.1.6 Studying Physiological Mechanisms by Regulating Enzyme Activity

Biological processes in nature are fine-tuned with exact spatial and temporal control across molecular, cellular, and organismal scales. The incorporation of light-triggered ncAAs into proteins via GCE has enabled precise, spatiotemporal control of various protein functions within cells and organisms through optical manipulation to studying physiological mechanisms.^266^ Due to nitrobenzyl-based ncAAs widely used in on-demand activation of proteins for biomedicine purposes (see section 4.2.3), this kind of photocaged ncAAs has also been applied to regulate protein functions for studying physiological mechanisms. For example, Palei et al.^612^ introduced a novel method for activating ten-11-translocation (TET) dioxygenases within mammalian cells by genetically incorporating photocaged ncAA, 4,5-dimethoxy-2-nitrobenzyl-l-serine (DONBS) by an amber suppressor leucyl-tRNA-synthetase (LRS)/tRNA^Leu^ pair^613,614^ to replace active residues. The caging group as a transient active-site inhibitor can be subsequently removed using light-induced deprotection, thus providing a means to gain precise insights into the effects of cancer-associated mutations in TET2 on the catalytic kinetics of TET2 in vivo. Additionally, a photocaged variant of lysine (pc-Lys) was incorporated in (lymphocyte-specific protein tyrosine kinase) LCK* via GCE. Liaunardy-Jopeace et al.^274^ demonstrated that CD4 and CD8 (the T cell coreceptors), can augment LCK activity, shedding light on their role in physiological T-cell receptor (TCR) signaling. Importantly, their approach offered valuable insights into the dynamics of SRC-family kinases beyond LCK, contributing to broader understanding of these kinase proteins. Courtney et al.^272^ utilized another photocaged lysine analogue hydroxycoumarin lysine (HCK) to light-responsively modulate the protein–protein interaction between MKP3 and ERK2, demonstrating nuclear translocation of ERK is modulated in a dose-dependent fashion as MKP3 activity increases. A similar strategy^615^ was applied for optically controlling the synthesis of 3-phosphorylated phosphoinositides by specifically replacing Lys464 residue of *Salmonella* type III secretion system effector protein SopB with HCK. The bulky hydroxycoumarin group was exposed to 405 nm illumination and photolyzed, SopB with phosphatase activity was restored, thereby promptly leading to the SopB-mediated synthesis of plasma membrane PtdIns(3,4)P2 via a phosphotransferase/phosphoisomerase mechanism. Using this photoactivatable SopB, they further demonstrated SopB requires PtdIns(4,5)P2 to generate PtdIns(3,4)P2 during *Salmonella* entry.

Besides these light-mediated deprotection reactions have been employed for the controllable release of proteins, bioorthogonal cleavage reactions that respond to metal or small molecules exhibit significant promise because of their adaptability, adjustability, and enhanced ability to penetrate and target specific locations within living tissues and animals.^616,617^ Based on glycogenin (GYG) as a “seed core” for the formation of the glycogen particle by autoglucosylating one of its own residues, Tyr195, which serves as an anchor to begin building the polysaccharide, Bilyard et al.^618^ devised an innovative approach by site-specific incorporation of l-*p*-iodophenylalanine (pIPhe) via amber codon suppression to replace the key residue Tyr195 of GYG, resulting in introducing an “OH → I” mutation. This mutation puts GYG into an “off” state, facilitating a palladium(Pd)-mediated activation that mimicked intermediate states of GYG. By meticulously mimicking GYG intermediates, they recapitulated catalytic activity at various stages, ultimately unveiling three distinct kinetic phases in the process of glycogenesis: “priming”, “extension”, and “refining”. Similar palladium(Pd)-mediated activation technique by incorporating ProcK with a chemical protective group (described in section 4.1.2.5) into a *Shigella* type III effector protein OspF at Lys134 position was used by Li et al.^285^ to study the intracellular localization of protein kinase Erk^619^ on irreversible dephosphorylation by OspF, demonstrating that Erk, once dephosphorylated by OspF, undergoes an irreversible transfer from the nucleus to the cytoplasm, followed by accumulation of damaged Erk in the cytoplasmic region, which thus addressed pathological impact of OspF on regulating Erk’s phosphorylation levels and consequently affecting the MAPK signaling pathway. On the other hand, Brown et al.^288^ developed the strategy (described in section 4.1.2.5) for utility of the phosphine (2DPBM)-decaged PABK switch with remarkable incorporation efficiency via GCE into zebrafish embryo GTPase protein NRAS.^620^ By temporally caging the constitutively active RASopathy mutant (NRAS G60E) with reduced GTPase activating protein (GAP) binding^621^ through replacing this Lys16 residue with PABK, they further added 2DPBM to the media for protein decaging activation, and gained insights into the mechanisms behind RASopathy-induced heart looping defects, suggesting that disordered looping may serve as a common initiator for congenital heart defects in RASopathies.

#### 4.3.2 Exploration of Bioinorganic Chemistry for Enzyme Mechanisms and Organic Chemistry

Bioinorganic chemistry explores the role of metals in biology, including metalloproteins like enzymes. GCE can be used in designing metalloproteins by incorporating ncAAs with reactive groups at key sites to form covalent bonds, revealing transient states or cofactors and providing insights into these proteins.^510,622^ Here we conclude these strategies.

**Figure 23:**
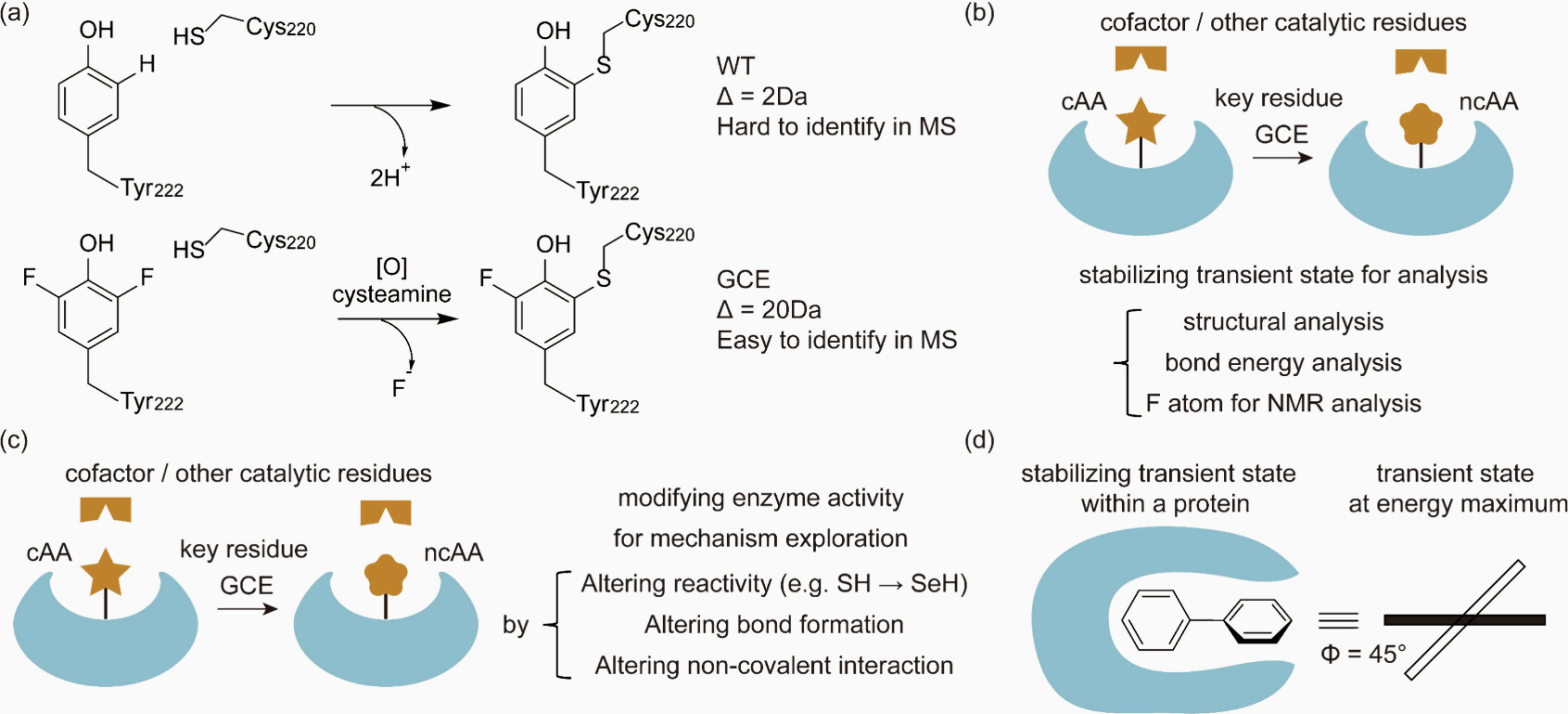
Exploration of bioinorganic chemistry and organic chemistry. (a) Using mass difference between cAA and ncAA to reveal enzyme mechanisms. More obvious loss of mass provide convenience for MS identification to detect reactions on the enzyme. MS, mass sprectrum. (b) Schematic illustration of stabilizing transient state structure for analysis. The ncAA at key residue cannot cross-link with cofactor as cAA, thus retaining transient uncrosslined state of enzyme for analysis. (c) Schematic illustration of changing enzyme activity for comprehensive mechanistic analysis. Substituting cAA with different ncAAs changes the activity of enzymes, providing insights into the mechanism of this enzyme. (d) Schematic illustration of using protein to stabilize transient state at energy maximum for organic chemistry study. The biphenyl is locked in the protein and keeps a transient state at energy maximum, enabling researchers to study this transient state.

The first strategy uses the mass difference between cAAs and ncAAs to reveal enzyme mechanisms (Figure 23a). Wang et al. used 3,5-difluoro-tyrosine (F2-Tyr) to identify the cofactor structure of cysteamine dioxygenase (ADO). By substituting Tyr222 with F2-Tyr, they detected which atom Cys220 attacks to form a Cys-Tyr cofactor, aiding in understanding ADO’s structure and mechanism.^623^

The second strategy involves stabilizing transient state structures for analysis, such as un-cross-linked or cross-linked enzyme-cofactor complexes (Figure 23b). Li et al. used F_2_-Tyr to weaken hydrogen bonding at a critical site, obtaining an uncross-linked crystal structure of a cysteine-metabolizing enzyme and observing oxidative C–F bond cleavage during cofactor formation.^624^ They also incorporated F_2_-Tyr and 3,5-dichlorotyrosine (Cl_2_-Tyr) into a copper enzyme, revealing novel copper-mediated C–F bond scission during cofactor biogenesis.^625^

A third approach involves modifying enzyme activity to analyze mechanisms (Figure 23c). Evans et al. substituted cysteine with selenocysteine in a hydrogenase enzyme and showed that the substitution increases the enzyme’s oxygen tolerance but does not enhance hydrogen production.^626^ Weaver et al. replaced tyrosine at a key position in photosynthetic reaction centers with tyrosine analogues, finding that this modification tunes primary electron transfer by modulating energy levels.^627^ Cheng et al. studied a biosynthetic pathway, finding that substituting a key sulfur-containing intermediate with a selenium analogue does not produce the expected product but does catalyze deuterium exchange, suggesting a carbene intermediate.^628^ Chen et al. measured the kinetic isotope effect in enzyme-catalyzed reactions by replacing an active site tyrosine with a methylthio-tyrosine analogue, revealing that a common intermediate regulates the balance between two reaction pathways.^629^

GCE can also facilitate organic chemistry research. Pearson et al. used genetically incorporated *p*-biphenylalanine (BiPhe) to mimic transient states of biphenyl.^630^ The π-stacking interactions within the core of the designed protein stabilized the planar TS conformation, enabling crystal structure analysis of biphenyl trapped in the coplanar TS conformation during bond rotation (Figure 23d).

### 4.4 Other Applications

#### 4.4.1 Biomaterials

The application of GCE in the development and enhancement of biomaterials has emerged as a groundbreaking technique that significantly extends the capabilities and functionalities of both natural and synthetic materials in biotechnology and medicine.^631^ By enabling the incorporation of ncAAs into proteins, GCE allows for the precise modification and optimization of biomaterials, leading to advancements in a variety of applications including drug delivery, tissue engineering, and biosensing technologies.^5^

**Figure 24:**
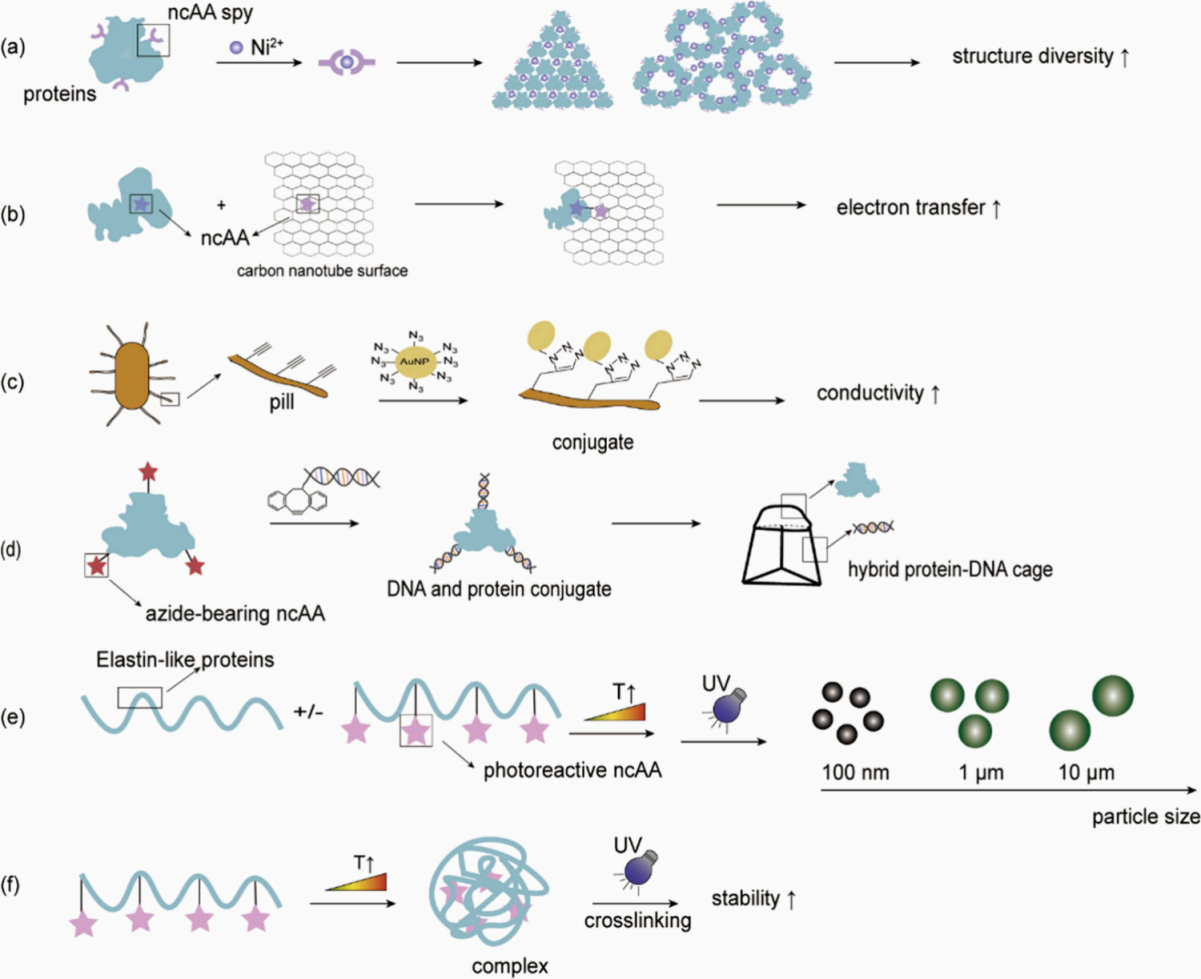
Applications of GCE in biomaterials. (a) The genetically incorporated bidentate bipyridyl-alanine enables controlled protein self-assembly, preserving protein functionality and enhancing thermal stability. (b) The genetically incorporated *p*-thiolphenylalanine facilitates the biosynthesis of protein and carbon nanotube hybrid biomaterials. (c) The genetically incorporated propargyloxy-phenylalanine allows for site-specific cross-linking, creating pili complexes with gold nanoparticles. (d) The genetically incorporated azide-bearing ncAA enables site-specific cross-linking, forming hybrid protein–DNA cages. (e) The genetically incorporated photoreactive ncAA allows tuning the size of elastin-like polypeptides. (f) The genetically incorporated photoreactive ncAA enhances the stability of elastin-like polypeptides.

Recent years have witnessed increased interest in optimization and enrichment of the properties of the protein biomaterials by GCE.^5^ Bar et al. have highlighted the potential of integrating photoswitchable azobenzene-bearing nonstandard amino acids for modulating polymer structures and crafting light-responsive nanostructures.^407^ Similarly, Minwoo et al. demonstrated the controlled protein self-assembly through the incorporation of bidentate bipyridyl-alanine, a process propelled by metals that not only preserve protein functionality but also bolster thermal stability (Figure 24a).^632^ By harnessing the genetical incorporated *p*-thiolphenylalanine, it is possible to wire the enzyme to the electrode through a thiol–chlorine nucleophilic substitution reaction with potential applications in wearable sensing devices (Figure 24b).^633^

In addition to tunning the properties of proteins, the incorporation of ncAAs introduces unprecedented functionalities and complexities, thanks to site-specific cross-linking and conjugation of diverse materials. The most universal combination is GCE with click chemistry reactions such as CuAAC, SPAAC, and IEDDA cycloaddition reaction. Daniel et al. reported the construction of protein nanowires with high electronic conductivity and demonstrated about 170 fold increase in the electronic conductivity facilitated by CuAAC reaction to combine the protein material—pili with gold nanoparticles (Figure 24c).^634^ Hidetoshi et al. incorporated the AzF into silk fiber in vivo and conjugate the silk fiber with fluorescent molecules using SPAAC reaction which enhances the aesthetic appeals and expands their utility into other applications.^635^ SPAAC conjugation also enable the polymerization of DNA and proteins to generate a hybrid DNA–protein cage with tunable dimensions (Figure 24d).^636^

Light-responsive cross-linking is another frequently used method. The integration of photo-cross-linkable AzF into biopolymers aids in generating hydrogel particles whose dimensions span from nanometers to micrometers, effectively stabilizing thermally responsive hydrogels (Figure 24e)^637^ Through the incorporation of multisite photoreactive AzF, the microparticles derived from artificial intrinsically disordered proteins ensures their covalent stabilization and simplifies their collection (Figure 24f).^638^

#### 4.4.2 Biosensors/Sensing

**Figure 25:**
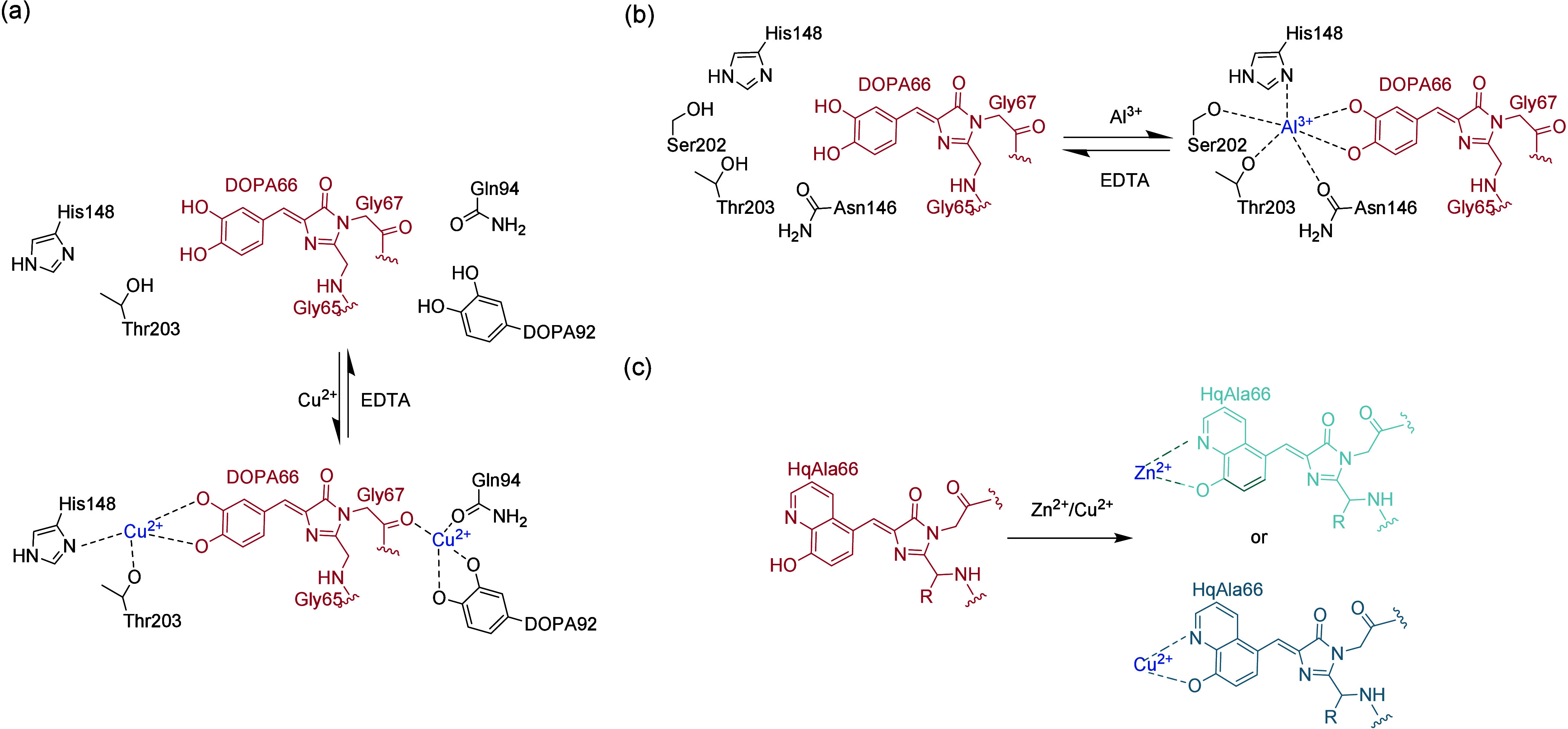
Biosensors for transition metal ion detection. (a) The Cu^2+^ sensor based on L-DOPA. (b) The Al^3+^ sensor based on L-DOPA. (c) The Zn^2+^/Cu^2+^ sensor based on HqAla.

**Figure 26:**
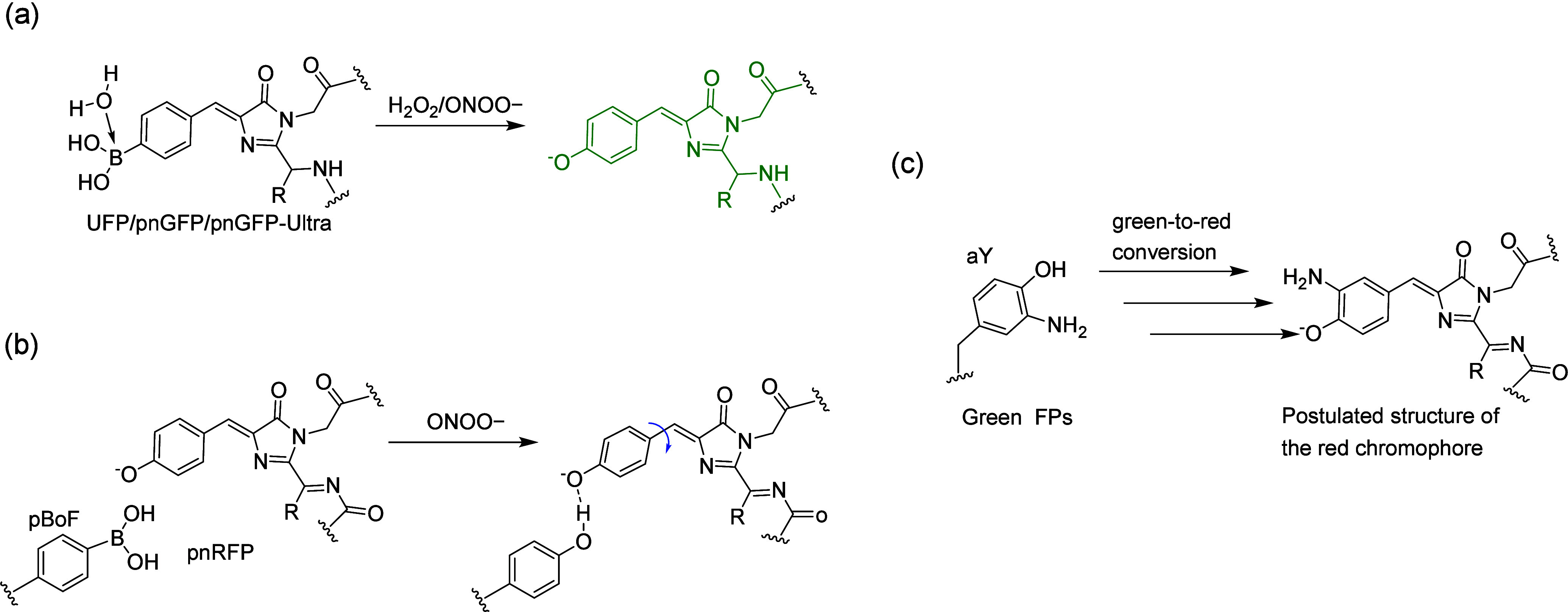
Representative FP-based biosensors for H_2_O_2_/ONOO^–^detection and imaging. (a,b) Biosensor based on pBoF for H_2_O_2_/ONOO^–^ detection. Introduction of pBoF to the chromophore of UFP, pnGFP, or pnGFP-Ultra allowed it to react with peroxynitrite and form a tyrosine-derived chromophore that enhanced fluorescence (a). pBoF is introduced to an amino acid position near the chromophore of pnRFP, where its reaction with peroxynitrite creates a tyrosine residue, which reduces fluorescence by bending the chromophore through hydrogen bonding (b). (c) Biosensor based on aY for imaging. The postulated structure of the chromophore in the a Y-converted red FPs was shown.

Conventional protein biosensors usually require the protein to be labeled through chemical or genetic methods. This process adds an extra molecule to the original protein, which can change its structure and behavior. Although these labeling techniques have been successful in certain cases, they have various limitations in terms of their preparation and application.^639^ An alternative method involves incorporating ncAAs that function as labels into the protein structure through GCE. This approach affects the protein’s immediate surroundings but has a less significant impact on its overall structure, which allows the protein to be used for detecting specific substances or biological events in living cells or organisms, enabling real-time observation and analysis. To date, a novel category of fluorescent protein (FP)-based biosensors incorporating ncAAs as the detection component has emerged. The distinct chemical and physical characteristics of these ncAAs facilitate the creation of sensor designs unachievable with the standard 20 cAAs. For example, metal-binding ncAA (3,4-dihydroxy-l-phenylalanine, Dopa; 2-amino-3-(8-hydroxyquinolin-5-yl)propanoic acid, HqAla) -containing FP biosensors (Figure 25) for detection of metal ions (such as Cu^2+^, Zn^2+^, Al^3+^) were developed previously.^640−642^ Additionally, fluorescent molecules containing boronic acid have been widely used for detecting hydrogen peroxide (H_2_O_2_) and peroxynitrite (ONOO^–^), which are important reactive oxygen and nitrogen species in biological systems. Based on GFP_UV_, a variant of GFP enhanced for maximum fluorescence under standard UV light, Wang et al.^643^ engineered a H_2_O_2_ FP biosensor named UFP-Tyr66pBoPhe by substituting the GFP’s natural chromophore-forming Tyr66 residue with the synthetically incorporated boronic acid-containing ncAA pBoF (Figure 26a). Upon exposure to H_2_O_2_, pBoF can be converted to tyrosine, which generates the fluorophore of GFP_UV_, and the change in fluorescence intensity was directly proportional to H_2_O_2_ concentrations within the micromolar range. The in vitro evaluation of the purified FP sensor revealed its sensitivity to low micromolar levels of H_2_O_2_. Compared to previously reported H_2_O_2_ FP sensors (HyPer, HyPer-2, and HyPer-3),^644−646^ UFP-Tyr66pBoPhe showed at least 20-fold enhancement in dynamic range. Furthermore, the treatment of H_2_O_2_ to *E. coli* harboring the UFP-Tyr66pBoPhe sensor caused nonfluorescent cells to emit bright green fluorescence, demonstrating its effective application in biological environments. Additionally, Chen et al.^647^ developed a genetically encoded ONOO^–^-specific green fluorescent biosensor (pnGFP) by integrating pBoF into the chromophore of a circularly permuted green fluorescent protein (cpGFP) (Figure 26a). Another enhanced variant of pnGFP, pnGFP1.5, was also developed, which exhibits better folding and expression while maintaining its exceptional specificity for ONOO^–^. Utilizing directed evolution, rational design, and targeted reactivity screening guided by the crystal structure of pnGFP1.5 variant pnGFP1.5-Y.Cro, they further engineered another high-performance, genetically encodable biosensor pnGFP-Ultra for selectively and sensitively imaging ONOO^–^ in mammalian cells,^648^ featuring a *p*-boronophenylalanine-modified chromophore with minimal cross-reactivity to other reactive species, and an efficient ncAA expression system for broad mammalian cell application, significantly advancing the molecular tools available for studying reactive nitrogen species biology. Similarly, Pang et al.^649^ extended this concept by introducing pBoF into amino acid residues situated near the chromophore of an enhanced circularly permuted red fluorescent protein (ecpApple), yielding two responsive ecpApple mutants: one exhibiting reactivity toward both ONOO^–^ and H_2_O_2_, while the other, named pnRFP, serves as a highly selective red fluorescent ONOO^–^ biosensor (Figure 26b). Notably, the boron atom in pnRFP adopts a sp^2^-hybridization configuration within a hydrophobic pocket, and pnRFP reacts with ONOO^–^ to generate a product with a twisted chromophore, validating the observed “turn-off” fluorescence response. In spite of the advantages for red fluorescent protein (RFP)-based biosensors over GFP-based ones, such as less phototoxicity, lower autofluorescence, and better tissue penetration, RFP biosensors face challenges like limited dynamic range, mislocalization, and undesired photoconversion, and there are few options available, with each requiring significant effort to develop. Zhang et al.^650^ presented a versatile approach that utilizes a genetically encoded 3-aminotyrosine (aY), to chromophores in green fluorescent protein-like proteins and biosensors containing tyrosine-derived chromophores, enabling a spontaneous and effective green-to-red shift through additional oxidation of aY (Figure 26c). This approach efficiently expands the range of RFP biosensors while maintaining their original properties like brightness, dynamic range, and responsiveness. They also successfully used these modified biosensors for detailed imaging of metabolic activities in pancreatic β-cells.

**Figure 27:**
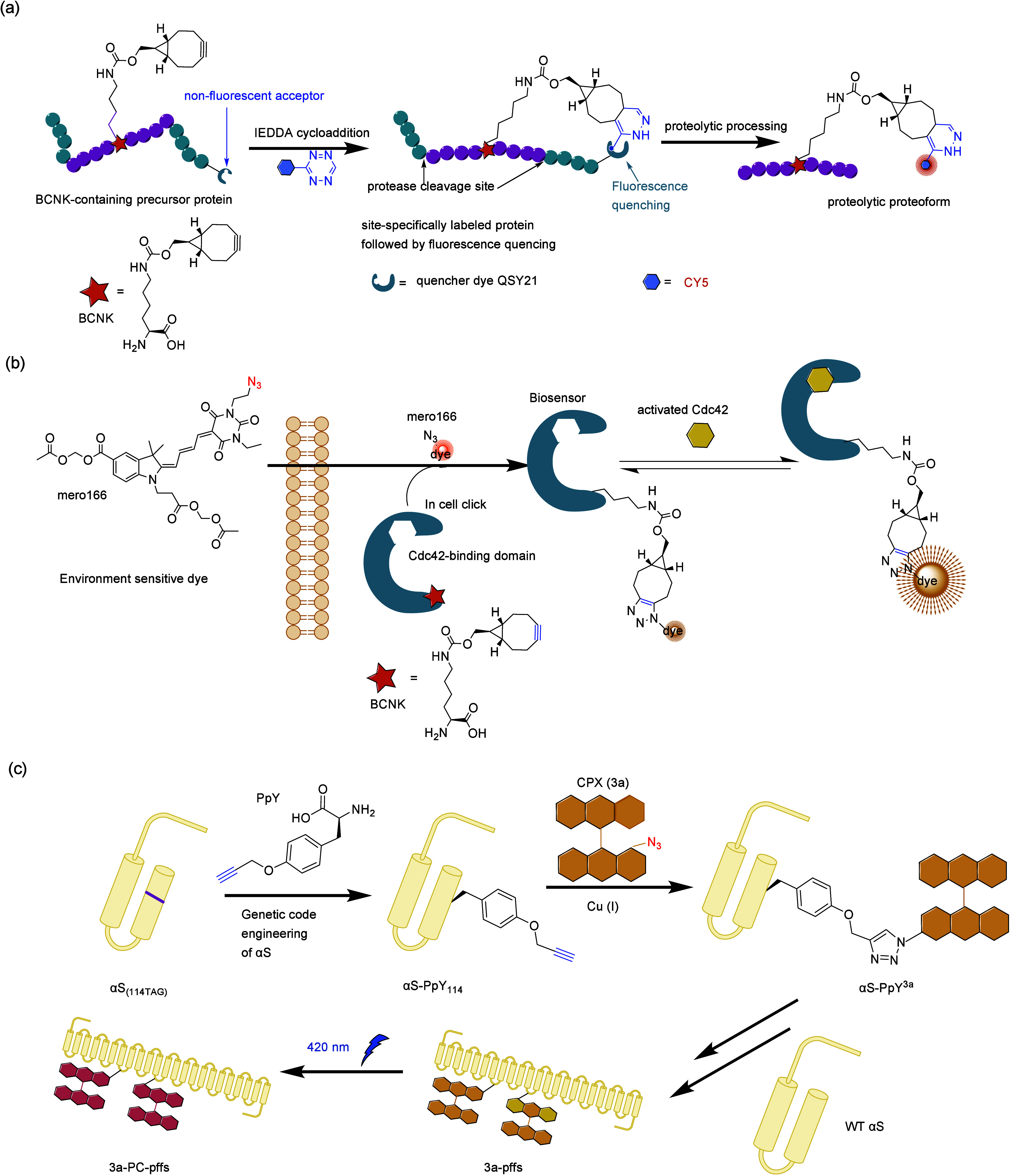
Small-molecule fluorescent probe-based biosensors. (a) Protein labeling and identification of proteolytic proteoform using a GCE-based molecular beacon. Polypeptides containing BCNK are modified with a fluorophore through IEDDA cycloaddition. A corresponding fluorescence quencher connected to the same protein suppresses the fluorescence. The fluorescence signal is produced when the fluorophore and quencher are separated through proteolysis. (b) Membrane-permeant, environment-sensitive dye mero166 generates a biosensor for the small GTPase Cdc42 within living cells. (c) Overview of producing 3a-tagged αS-PpY114 preformed fibrils (3a-pffs) and their subsequent photoconversion to fibrils (3a-PC-pffs).

In addition to the development of FP-based biosensors for analyte detection and imaging, GCE also has been widely used for small-molecule fluorescent probe-based biosensor construction to study the temporal and spatial dynamics of proteins within living cells. Sappakhaw et al.^651^ designed a doubly labeled fluorescent reporter APP(H609BCNK)-HaloTag-(QSY21), in which the amyloid-β (Aβ) portion of amyloid precursor protein (APP) is labeled with tetrazine-cy5 via IEDDA cycloaddition with the ncAA BCNK genetically incorporated at site 609, and the C-terminus of APP tagged with quencher dye QSY21 (a nonfluorescent acceptor with optimal absorbance for quenching donor dye Cy5)^652^ conjugated to a HaloTag chloroalkane ligand (Figure 27a). The molecular beacon design situates the Förster resonance energy transfer (FRET) donor, cy5, on the extracellular facet of APP, while the FRET acceptor, QSY21, is positioned on the cytosolic side. In view of beacon arrangement, cy5 located on the Aβ portion of APP is expected to be quenched by an intramolecular HaloTag-linked QSY21, resulting in fluorescence only when Aβ is completely liberated from APP. This prototype reporter serves as a valuable tool for monitoring amyloid precursor protein processing and intracellular Aβ production, offering a straightforward means to track Aβ generation within cells, particularly in neurons, and can be seamlessly integrated with high-content imaging platforms for the screening of compounds capable of modulating Aβ production. Additionally, introducing environment-sensitive dyes into living cells is usually challenging in order to study the temporal and spatial dynamics of proteins, MacNevin et al.^653^ addressed this issue by improving the environment-sensitive fluorescent moiety of dyes to create azide-containing merocyanine (mero166) which can attach to ncAA BCNK with incorporation by a variant of the *M. barkeri* (Mb) RS/tRNA^654^ at residue 271 of the small GTPase Cdc42 binding domain derived from the Wiskott–Aldrich syndrome protein^655,656^ through a copper-free click reaction between BCN and azides (Figure 27b). Attaching the environment-sensitive fluorophore at this location led to the creation of a biosensor that exhibited increased fluorescence intensity upon binding to activated, GTP-bound Cdc42 within cells. Jun et al.^657^ made a clickable and photoconvertible diazaxanthilidene (CPX) probe by introducing an orthogonal “clickable” linker to a diazaxanthilidene fluorophore, which can be linked to any target through a diazirine cycloaddition reaction. To demonstrate the utility of CPX probe (3a), αS were conjugated with 3a at position 114 which was site-specifically replaced and incorporated by propargyl tyrosine (PpY) via GCE, yielding αS-PpY3a. After purification, the monomeric αS-PpY3a protein constructs were assessed for photoconversion and aggregated to demonstrate that these fibrils labeled with 3a could undergo photoconversion by in vitro irradiation without degradation (Figure 27c). Since the attachment of other fluorescent dyes (e.g., BODIPY) at site 114 did not disrupt the structure, they employed 3a-labeled αS-PpY114 preformed fibrils (3a-pffs) to investigate the internalization and endolysosomal trafficking of αS, suggesting that CPX can serve as a minimally perturbing probe for tracking the dynamics of biomacromolecules.

**Figure 28:**
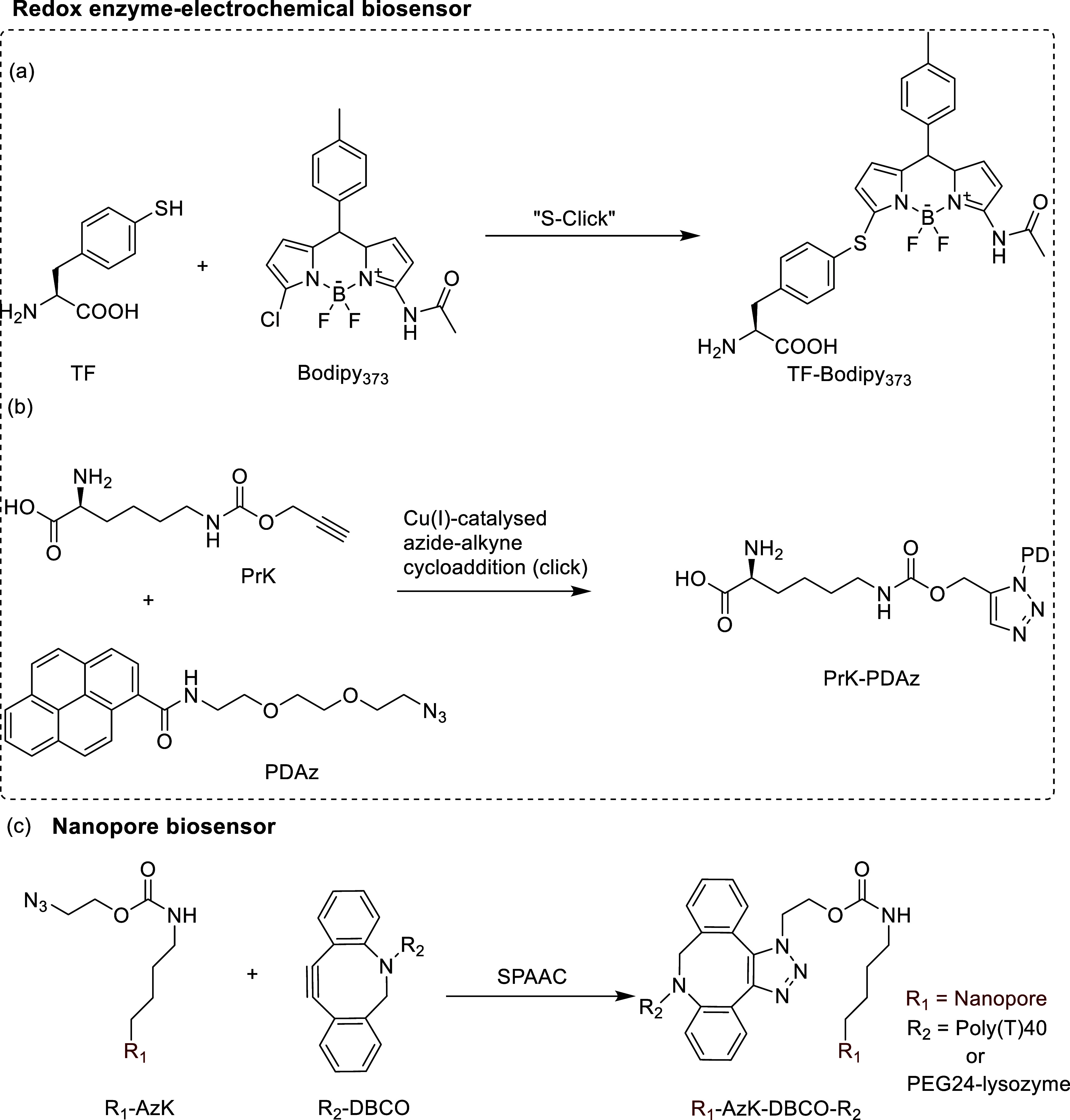
Redox enzyme-electrochemical biosensor and nanopore biosensor. (a) A schematic illustration of thiol-chlorine nucleophilic substitution reaction (S-click reaction): sulfur atom in TF (S), chlorine atom in Bodipy373 (Cl). (b) A schematic illustration of PrK and pyrene-diethylene glycol-azide (PDAz) with copper(I)-catalyzed azide–alkyne cycloaddition (click) reaction to a pyrene-diethylene glycol-azide (PDAz). (c) DBCO was covalently attached to AzK via a strain-promoted alkyne–azide cycloaddition reaction (SPAAC).

On the other hand, redox enzyme-electrochemical biosensors are also attractive because of their typically fast response, high specificity and sensitivity, affordability, and portable dimensions.^658^ Thus, establishing an effective electron transfer (ET) pathway between redox enzymes and electrodes is crucial for converting enzyme-catalyzed reactions into electrochemical signals and developing robust, sensitive, and selective biosensors. To this end, Xia et al.^633^ incorporated 2-amino-3-(4-mercaptophenyl)propanoic acid (or *p*-thiolphenylalanine, TF) specifically into amino acid oxidase L-tryptophan oxidase (TrpOx) by a newly evolved TFRS/tRNA. The thiol group of TF is specifically coupled with a chlorine group on enzyme/electrode wiring linker boron-dipyrromethene (Bodipy373) through a thiol-chlorine nucleophilic substitution reaction (S-click reaction) (Figure 28a), thus facilitating electron transfer. The resulting biosensor TrpOx-395TFBodipy373/CNT/GCE can provide real-time and selective monitoring of tryptophan (Trp) in blood and sweat samples, with a linear range of 0.02–0.8 mM. Similarly, based on the previous construction of highly efficient flavin–adenine dinucleotide (FAD) glucose dehydrogenase (GDH) fused to a minimal cytochrome c domain (MCD) (FGM) for glucose detection,^659^ Algov et al.^660^ engineered the fusion enzyme FGM or GDH with site-specific incorporation of propargyl-l-lysine (PrK) into GDH, generating three FGM/GDH mutants FGM-S247PrK, GDH-S247PrK and FGM-T558PrK, in combination with copper(I)-catalyzed azide–alkyne cycloaddition (click) reaction to a pyrene-diethyleneglycol-azide (PDAz) to enable control over its orientation toward a glassy-carbon electrode (Figure 28b). Wiring these mutant enzymes at different sites positively affects their ET characteristics and ability to communicate with the electrode. These site-specifically wired enzyme variants were showed had a significantly faster rate of electron transfer, exhibiting an *i*_max_ over 15 times higher and a sensitivity 10 times greater than their nonspecifically wired counterparts. This approach allows for highly flexible ncAA site-specific incorporation, making it a powerful tool for advanced protein engineering in enzyme-based biosensors. When positioning glucose oxidase on the electrode surface, improved sensitivity (down to a concentration of 10 μM glucose) was observed. This makes the enzyme more suitable for noninvasive biosensors used to detect body fluids other than blood, such as sweat, tears, and urine. Furthermore, they^661^ also designed another enzyme-based biosensor using a copper efflux oxidase CueO with PrK at specific sites. Purified CueO with PrK (CueOD411PrK) was subsequently linked specifically to an electrode by employing a click reaction between the alkyne residue of PrK and the azide residue of the electrode-binding linker PDAz. This process enables high-resolution and highly sensitive detection of dopamine (DA) and epinephrine (EN). They presented an enzyme-based biosensor capable of detecting these molecules in artificial sweat samples with a resolution as low as 10 nM and a linear range of up to 100 nM. This biosensor can be used for the determination of catecholamines in different bodily fluids.

Moreover, the intriguing field of nanoscience also offers a plethora of opportunities and challenges in creating various biosensing systems at the nanoscale. The protein nanopore is a promising biosensor for rapid and cost-effective single biomolecule detection, with its sensitivity and accuracy dependent on its structure and chemical environment.^662−664^ However, despite advancements in customizing these nanopores, challenges in sensitivity and reproducibility persist, leading to increased focus on modifying naturally occurring protein nanopores like α-hemolysin (α-HL),^665^*Mycobacterium smegmatis* porin A (MspA),^666^ and aerolysin (AeL).^667^ NcAAs feature a variety of chemical functional groups absent in cAAs, providing a means to customize the chemical environment of the sensing interface. For example, Yang et al.^668^ successfully introduced bio-orthogonal reaction groups into the pore rim of the M2MspA mutant nanopore^669^ by respectively incorporating ncAAs *N*^6^-(2-azidoethoxy)carbonyl-l-lysine (AzK) containing an azide group and *N*^6^-((pent-4-yn-1-yloxy)carbonyl)-l-lysine (AlkK) containing an alkyne group, into a specific site (D56) of M2MspA via different orthogonal RS/tRNA_CUA_ pairs. To validate the reactivity of azido-containing M2MspA-D56AzK, dibenzocyclooctyne (DBCO)-functionalized single-stranded DNA or lysozyme was attached to the nanopore rim using the SPAAC click reaction (Figure 28c) and observed the real-time motion of these biomacromolecules at the single-molecule level. Importantly, the chimeric nanopore containing lysozyme was able to sense and differentiate between *N*-acetylglucosamine trimer (NAG3) and *N*-acetylglucosamine hexamer (NAG6), a feat not achieved by any other nanopores before. This enzyme-nanopore coupling strategy allows for the real-time monitoring of enzyme-catalyzed reactions, providing more dynamic information and measurement continuity. In contrast to Yang’s work,^668^ Wu et al.^670^ provided a new framework for more precision and flexibility in tuning the sensing attributes of nanopore by integrating *N*^6^-carbobenzoxy-l-lysine (CbzK) into the position K238 within AeL nanopore’s inner sensing area. CbzK features an extended side chain incorporating a hydrophilic peptide bond at its center, enabling a flexible geometric alignment within the hydrophilic channel of AeL, which markedly boosts its capacity to detect a broad spectrum of analytes, including peptides. This work presents a simpler, more universal strategy for adjusting nanopore sensing abilities, with potential uses in sectors like biotechnology and diagnostics. Significantly, GCE technology enables the incorporation of ncAAs in both prokaryotic and eukaryotic cells, which can be further developed as a key component in the toolbox for creating highly sensitive nanopore biosensors or versatile molecular machines with precision and adaptability in future applications.

## 5 Conclusion and Perspective

In summary, we have provided a comprehensive review of the recent developments, optimizations, and applications of GCE technology. In the past two decades, a wide variety of ncAAs have been crafted, offering distinctive features such as light-responsive elements, metal ion interaction, groups with unique chemical reactivity, advanced spectroscopic markers, electron transfer capabilities and innovative spatial attributes. Incorporating ncAAs with these varied structures and functions into proteins offers novel ways to explore, visualize, manipulate, and develop proteins, alongside creating innovative therapeutic agents. However, significantly modifying structure of amino acids to be recognized by the translation machinery and overcoming constraints in incorporating novel elements for protein studies are still major hurdles. In other words, the structure of amino acids cannot be changed arbitrarily, or they will not be properly used in the protein synthesis process. For example, in biophysical studies of proteins, the use of powerful fluorophores has its constraints, since fluorophores usually have significant structural differences from cAAs, which makes it difficult for these amino acids to be recognized and accepted as substrates by the translation machinery. Also, in the synthesis of unnatural oligomers, there are limitations on the types of backbone analogues that can be incorporated. Even so, significant advances have been made in optimization methods such as aaRS/tRNA engineering and modifications to the translation systems including orthogonal ribosomes for encoding and marking ncAAs with specific groups are particularly useful for adding diverse functionalities to specific protein sites. Efforts has also been made for developing a broad set of orthogonal codons that can be efficiently and accurately decoded, creating compatible aaRS/tRNA systems for these codons, and widening the chemical variety that cellular translation can handle. Currently, we are equipped to modify translation processes and ribosome structures to extents unimaginable before, thanks to GROs, in vivo tethered ribosomes, and a diverse repertoire of ncAAs. These advances facilitate the specific selection of orthogonal ribosomes compatible with an enlarged repertoire of ncAAs and codons. In addition, the dearth of attempts for developing ncAA biosynthetic pathways can be attributed to the scarcity of verified metabolic pathway and challenges of the potential inhibitory effects of ncAAs on host strains during biosynthesis. Future research is needed to elucidate metabolic pathways and engineer more resilient or metabolically balanced chassis strains.

Moreover, the GCE field is currently in a thrilling phase, thanks to the capacity for generating a wide variety of proteins containing ncAAs in *E. coli* with high efficiency, coupled with advancements in bio-orthogonal methods, and the expansion of GCE applications across various organisms, from bacteria to mammals, and even to animals was also achieved, highlighting the vast potential of GCE to revolutionize synthetic biology, which opens up new avenues for studying protein function, designing novel proteins, and developing therapeutic interventions. As the GCE technology further advances, ncAA incorporation is set to become a key technique for probing fundamental queries in systems biology, and it will increasingly be utilized as a novel approach in drug development. Therefore, the use of this technology in the creation of antibody–drug conjugates and vaccine development is probably just the beginning of its therapeutic applications. The roles of ncAAs in synthetic biology could also extend to creating synthetic cells with novel functions, requiring engineered pathways, compatibility, and expanded coding sets for ncAA incorporation. The mining of natural diversity for new orthogonal systems, the need for rapid validation methods for orthogonality, and the promising avenue of creating orthogonal ribosomes for in vivo selection are also in full swing. Furthermore, advancing biochemical and machine learning techniques will enhance the application and design of artificial enzymes with ncAAs, broadening the scope of functional macromolecules beyond natural counterparts. The future challenges include improving the efficiency and orthogonality of the components involved in GCE, expanding the range of usable ncAAs, and further integrating GCE technology with other synthetic biology tools to create complex biological systems with novel functions.
